# Base-Promoted Cascade Reactions for the Synthesis of 3,3-Dialkylated Isoindolin-1-ones and 3-Methyleneisoindolin-1-ones

**DOI:** 10.1021/acs.joc.1c01794

**Published:** 2021-10-06

**Authors:** Antonio Macchia, Francesco F. Summa, Antonia Di Mola, Consiglia Tedesco, Giovanni Pierri, Armin R. Ofial, Guglielmo Monaco, Antonio Massa

**Affiliations:** † Dipartimento di Chimica e Biologia “A. Zambelli”, Università degli Studi di Salerno, Via Giovanni Paolo II, 84084 Fisciano, Salerno, Italy; ‡ Department Chemie, Ludwig-Maximilians-Universität München, 81377 München, Germany

## Abstract

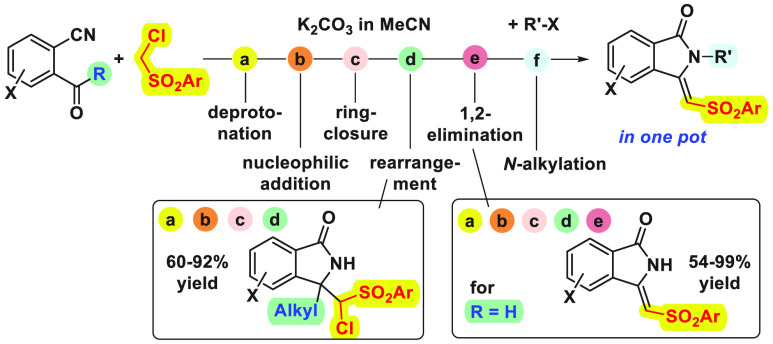

Cascade reactions of *ortho*-carbonyl-substituted benzonitriles with ((chloromethyl)sulfonyl)benzenes as pronucleophiles led to new isoindolin-1-ones with a tetrasubstituted C-3 position or to (*Z*)-3-(sulfonyl-methylene)isoindolin-1-ones. The reactions start from readily available materials, are carried out under mild conditions, and do not require metal catalysis. Promoted only by the cheap and environmentally benign K_2_CO_3_ as the base, up to six elemental steps can be combined in a single pot. Hence, a sequential one-pot cascade/β-elimination/alkylation furnished useful intermediates for the synthesis of aristolactam natural products. The observed selectivity and the mechanism were investigated by DFT studies.

## 1 Introduction

**Figure 1:**
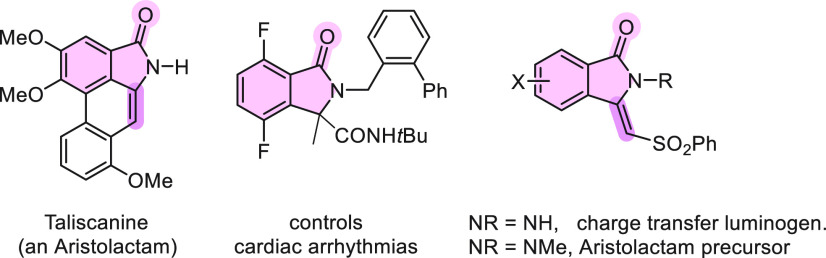
Isoindolin-1-one core motifs in bioactive and functional materials.

Recently, heterocyclic compounds bearing isoindolin-1-one and 3-methyleneisoindolin-1-one motifs have received increased interest owing to both their biological activities and their properties as functional materials.^1−6^ For example, taliscanine, a natural product isolated from *Aristolochia taliscana*, shows a range of promising activities on CNS, such as in the treatment of Parkinson’s disease and Alzheimer’s disease.^1a,1c^ A difluoro-substituted isoindolinonecarboxamide with a tetrasubstituted C-3 was developed as a drug for the treatment of cardiac arrhythmias because of its potassium channel-inhibiting activity.^1d^ Finally, sulfonyl-substituted 3-methyleneisoindolin-1-ones are synthetic precursors of aristolactams.^2a^ Moreover, exo-methylene-substituted isoindolinones show unique mechanochromic properties as luminogens (Figure 1).^2b^

However, access to these materials is often rather challenging because of the necessity to use transition metals as catalysts, expensive additives, or harsh reaction conditions.^1−3^ In this context, one-pot cross-aldol-initiated cascade reactions of 2-formylbenzonitriles (2-cyanobenzaldehydes) with C–H-active compounds under mild basic conditions have been proven to provide reliable access to several classes of heterocycles, including a wide range of 3-substituted isoindolinones.^1b,4^ In addition, despite the well-known lower electrophilicities of ketones and the possibility of competitive enolization, we have recently found that 2-acylbenzonitriles also react with a range of pronucleophiles under similarly mild conditions to yield 3,3-disubstituted isoindolin-1-ones.^5^ These products could easily be related to bioactive analogues bearing a tetrasubstituted carbon, whose syntheses have been reported to be particularly challenging.^1b,1d,6^ Quantification of the electrophilicity of such *ortho*-carbonyl-substituted benzonitriles would avail the prediction of the scope and selectivities of these cascade reactions. However, our attempts to determine the electrophilicity of 2-acetylbenzonitrile by studying the kinetics of its reactions with carbanions of known Mayr nucleophilicity^7a^ were not conclusive. Nevertheless, these kinetic experiments indicated that the carbonyl group in 2-acetylbenzonitrile may well be accessible for reactions with α-halo-stabilized carbanions.^7b^ This type of carbanions carries a leaving group (LG) in the α-position, enabling them to undergo cyclopropanations with electrophilic C=C double bonds.^7b,7c^ Furthermore, deprotonated ((chloromethyl)sulfonyl)benzene (PhSO_2_CH_2_Cl) is the prototypical reagent for vicarious nucleophilic substitutions (VNS reactions) at electron-deficient arenes.^8^ When α-halo-stabilized carbanions are combined with ketones, the formation of oxiranes is expected (Darzens condensation).^9^ In only a few cases the corresponding halohydrins were isolated, which were obtained upon the protonation of the intermediate β-haloalkoxides formed in the carbon–carbon bond-forming step.^10^

As part of our interest in the synthesis and reactivity of heterocyclic compounds,^4e−4g,5,6b^ herein we describe the facile and straightforward access to novel 3,3-disubstituted isoindolin-3-ones and 3-methyleneisoindolin-1-ones by reactions of 2-carbonylbenzonitriles and ((chloromethyl)sulfonyl)benzenes. Even though an array of different competitive reactions could stem from the combination of such electrophiles and pronucleophiles bearing multiple functional groups, the proper selection of the reaction conditions allowed us to develop a common cascade route that led to different products. A mechanism of the developed processes is proposed based on DFT calculations, experimental outcomes, and previous works in the field.

## 2 Results and Discussion

The possibility of using ((chloromethyl)sulfonyl)benzene-derived carbanions carrying a leaving group (LG) in the α-position in reactions with 2-acylbenzonitriles **1** attracted our interest because the alkoxide intermediates, such as **4a**, generated upon nucleophilic attack at the carbonyl group have two options to form stable products: they may undergo either cyclization with the formation of epoxides (Darzens reaction, path a) or cyclization via nucleophilic attack at the cyano group (path b). Intrigued by this bifurcation in the mechanistic track, we investigated the reaction of 2-acetylbenzonitrile **1** with ((chloromethyl)sulfonyl)benzene (**2H**) more deeply under different reaction conditions (Table 1).

**Table 1:**
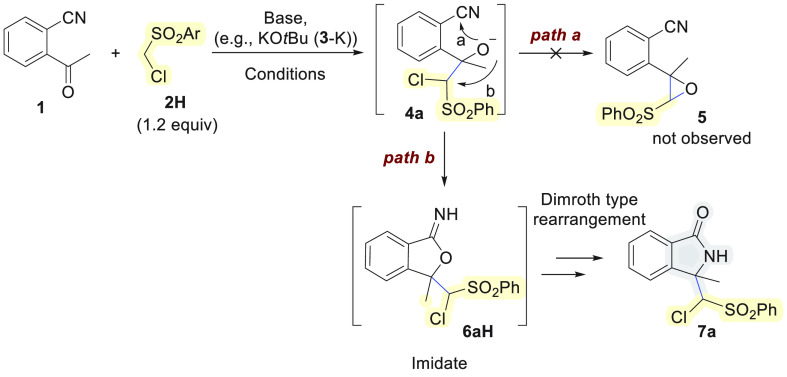
Cascade Reactions of 2-Acetylbenzonitrile (**1**) with ((Chloromethyl)sulfonyl)benzene (**2H**): Preliminary Screening

entry	base (1 equiv)	*T* (°C)	*t* (h)	yield (%)	d.r.
1^a^^,^^b^	KO^*t*^Bu	r.t.	24	dec	
2^b^^,^^c^	KO^*t*^Bu	r.t.	24	24%	2:1
3^c^^,^^d^	K_2_CO_3_	r.t.	24	n.r.	
4^c^^,^^d^	K_2_CO_3_	50	60	37%	1.7:1
5^c^^,^^d^	KO^*t*^Bu	r.t.	18	86%	2:1
6^c^^,^^d^	Et_3_N	50	60	n.r.	

a DMSO was used.

b [ketone] = 0.15 M.

c MeCN was used.

d [ketone] = 0.45 M.

Optimum results that led to clean reactions were obtained using KO^*t*^Bu (**3**-K) as base in a minimum amount of acetonitrile as the solvent (Table 1, entry 5), while in the presence of DMSO we observed the formation of a complex mixture of products (Table 1, entry 1). The use of weaker bases like K_2_CO_3_ did not guarantee good conversion (Table 1, entries 3 and 4), while Et_3_N was not effective (Table 1, entry 6). This is the first important outcome of the present study because to our knowledge only either weak bases like K_2_CO_3_ or tertiary amines or transition metals as catalysts have been used in the past to promote cascade reactions of 2-carbonyl benzonitriles.^4,5^ This may open new synthetic opportunities for less acidic pronucleophiles despite the possibility of the competitive enolization of such ketones. ^1^H NMR analysis on the crude revealed the formation of two diastereomers, which were purified by chromatography and then separated by fractional crystallization. The resulting crystals were suitable to the determine the product structure by X-ray analysis,^11^ which clearly highlighted the formation of an isoindolin-1-one with a quaternary carbon in the 3-position (*R*/*S* or *S*/*R* relative configuration for the major diastereomer) carrying a chloromethinephenylsulfonyl side chain (see the Supporting Information for further details). Therefore, the initial carbonyl addition reaction is presumably followed by cyclization at the cyano group instead of chloride displacement since we did not detect the epoxide formation corresponding to the Darzens reaction. Subsequently, the iminophthalan intermediate **6aH** rearranges to the isoindolinone structure **7a** via a Dimroth-type process (Table 1).^5^ This course of the reaction is in accordance with a report by Kobayashi and co-workers in which they showed that epoxide formation failed when they combined 2-formylbenzonitrile with dimethyloxosulfonium methylide.^4d^ The formation of the oxirane was outcompeted by the attack of the intermediately formed alkoxide oxygen at the nitrile group to generate a less-strained five-membered ring, which finally led to the isolation of 3-methyleneisoindolinones.^4d^

**Scheme 1:**
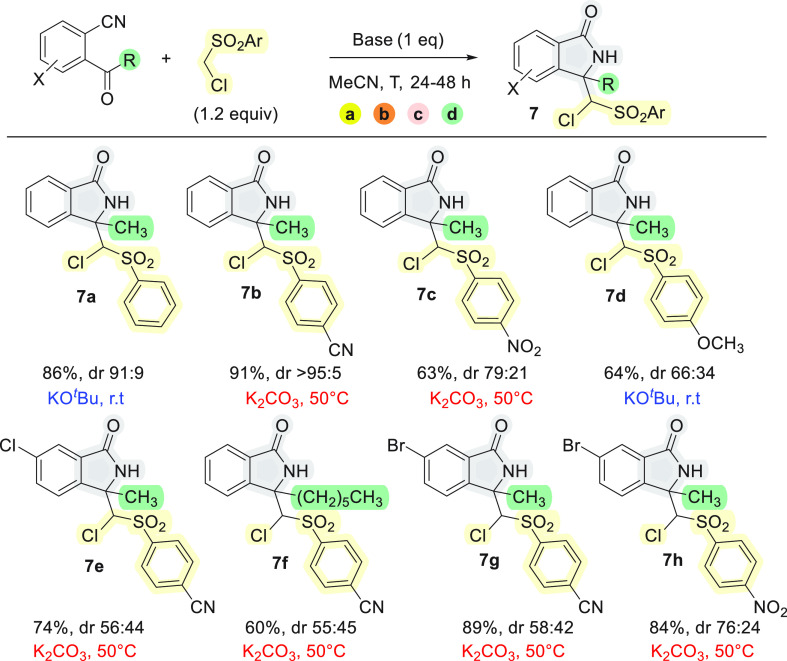
Scope of Cascade Reactions of 2-Acylbenzonitriles with ((Chloromethyl)sulfonylbenzenes

Next, the scope of the cascade reaction, which proceeds through (a) activation of the pronucleophile by deprotonation, (b) nucleophilic addition to the carbonyl group, (c) ring closure, and (d) Dimroth rearrangement of the heterocycle, was briefly analyzed in the presence of readily available 2-acylbenzonitriles substituted on the aromatic ring and different ((chloromethyl)sulfonyl)benzenes^12^ (Scheme 1). Pleasingly, all the tested combinations led to the isolation of the final products **7** in good to high yields, and the more acidic cyano- and nitro-substituted pronucleophiles gave better results in the presence of K_2_CO_3_ at 50 °C. Compounds **7b** and **7c** were obtained almost as single diastereomers. For crystallized **7a**, however, we observed slow epimerization when it was dissolved in either DMSO-*d*_6_ or CDCl_3_. The reaction is probably highly diastereoselective, but in only a few cases were the initial mixtures of diastereomers stable enough to be isolated and spectroscopically characterized. Finally, 2-heptanoylbenzonitrile also showed a useful reactivity, leading to a 3,3-substituted isoindolinone **7f** (60% yield) bearing a longer alkyl chain at C-3 and enlarging the synthetic perspectives of the cascade reaction developed in this work. In all the cases we attributed the *R*/*S* or *S*/*R* relative configuration for the analogy of the spectroscopy data to **7a**.

Additionally taking advantage of the work by Kobayashi,^4d^ we next investigated the possibility of synthesizing valuable arylsulfonyl-substituted 3-methyleneisoindolin-1-ones by the reaction of ((chloromethyl)sulfonyl)benzenes with 2-formylbenzonitriles. In fact, if a 3-monosubstituted isoindolin-1-one is formed via the (a) → (b) → (c) → (d) cascade, then the eventual β-elimination of HCl (step e) may lead to the desired unsaturated compounds. Nicely, the epoxide was never detected under the range of conditions described in Table 2. The respective 3-methyleneisoindolin-1-one **8a** was isolated in an almost quantitative yield when K_2_CO_3_ was used at 50 °C, while KO^*t*^Bu led to lower yield (Table 2, entry 2) and Et_3_N was not effective at all (Table 2, entry 4). The product **8a** was characterized by comparing its spectroscopic data with those reported in the literature,^3b^ and a (*Z*)-configuration was attributed to **8a**.

**Table 2:**
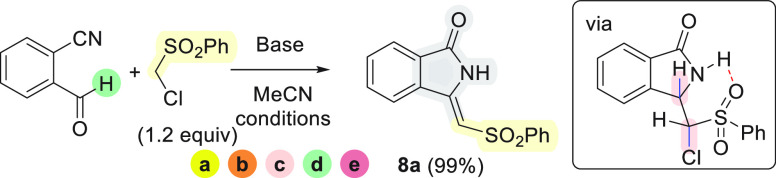
Cascade Reactions of 2-Formylbenzonitrile with ((Chloromethyl)sulfonyl)benzene: Preliminary Screening

entry	base (1 equiv)	*T* (°C)	*t* (h)	yield (%)
1	K_2_CO_3_	r.t.	48	41%
2	KO^*t*^Bu	r.t.	24	65%
3	K_2_CO_3_	50	48	99%
4	Et_3_N	50	48	n.r.

**Scheme 2:**
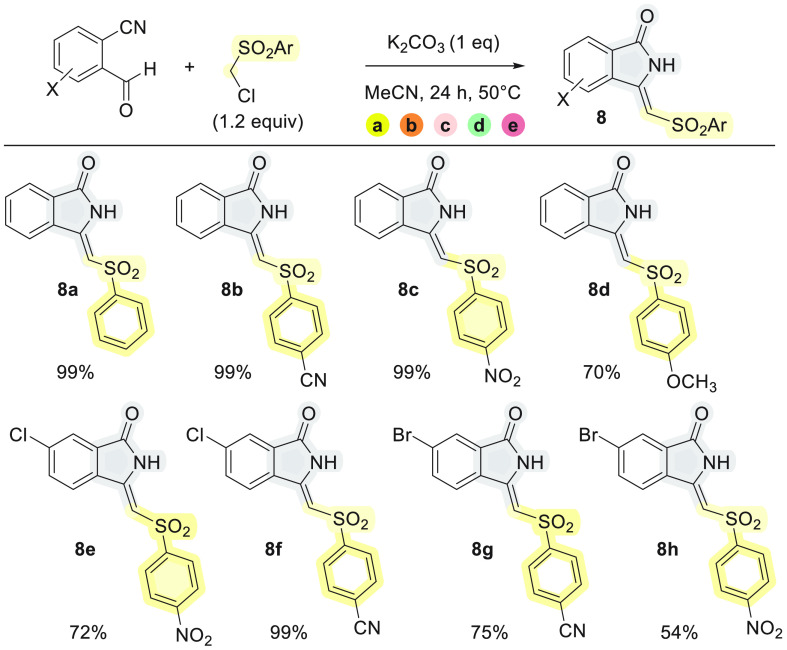
Scope of Cascade Reactions of 2-Formylbenzonitrile with ((Chloromethyl)sulfonylbenzenes

Obtaining 3-methyleneisoindolin-1-ones is a particularly attractive field, and in recent years several synthetic procedures have been published.^3^ To our knowledge, however, only one generally applicable protocol has been reported for the synthesis of **8**.^3b^ That work uses an elegant cyclization of aromatic nitriles with phenylvinylsulfone, which is promoted by a combined Ru(II)/Ag(I) catalysis and an excess of Cu(II) necessary for the oxidative cyclization. However, besides the necessity of two metal catalysts and a stoichiometric amount of oxidant under very harsh conditions, the use of only phenylvinylsulfone narrows this protocol to the products exclusively substituted on the isoindolinone ring.^3b^ Therefore, we analyzed the scope of our method, which uses readily available substituted 2-cyanobenzaldehydes and ((chloromethyl)sulfonyl)benzenes and may flexibly give rise to a series of 3-(sulfonyl-methylene)isoindolin-1-ones with electron-withdrawing groups (EWGs) and electron-donating groups (EDGs) on both aromatic moieties (Scheme 2).

**Scheme 3:**
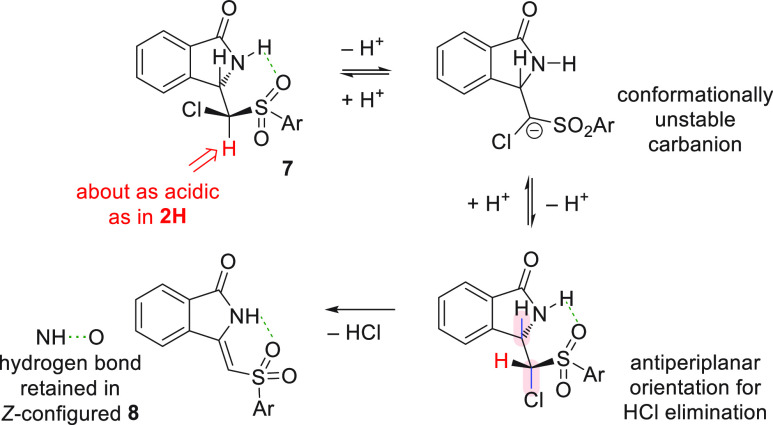
Epimerization Favors (*Z*)-Alkene Formation

With all the tested combinations of nucleophiles and electrophiles, we observed good to almost quantitative yields and (*Z*)-selectivity (Scheme 2). The (*Z*)-selective formation of methyleneisoindolinones **8** is rationalized by formation of an intramolecular H-bond between the NH and the SO_2_ groups in the intermediates that precede the final HCl elimination step. After step (d) of the cascade reaction, these intermediates are structural analogues of the isolated products **7** in which the H–C3–C–Cl bonds are presumed to be antiperiplanar in accordance with the −179° dihedral angle for H_3_C–C3–C–Cl observed in the solid-state structure^11^ of **7a**. This preorientation for HCl elimination provides stereoselective access to the (*Z*)-configured alkenes **8** in step (e) of the reaction cascade. Though **7** were isolated as mixtures of diastereomers (see Scheme 1), the labile C–H bond in the (chloromethyl)sulfonyl moiety facilitates epimerization with subsequent β-elimination under the basic reaction conditions (Scheme 3).

**Scheme 4:**
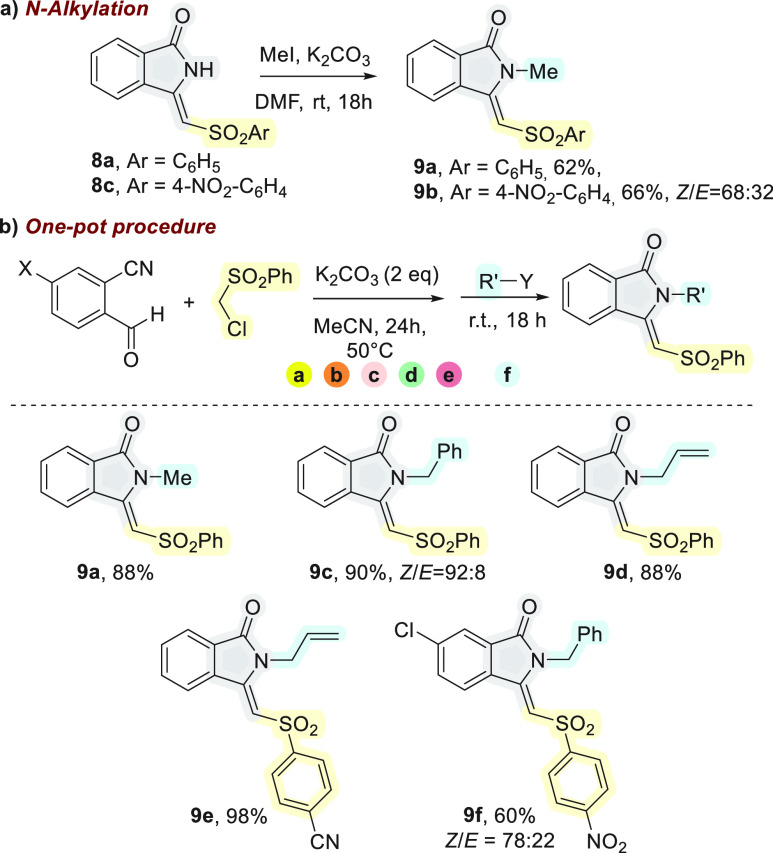
One-Pot Cascade Reaction/β-Elimination/*N*-Alkylation

The (*Z*)-configuration of **8** is a crucial prerequisite for the π–π stacking required to exert mechanochromic properties, as demonstrated by Hazra and co-workers.^2b^ In our case, access to differently substituted ((chloromethyl)sulfonyl)benzenes^12^ enables the synthesis of diverse (*Z*)-3-methyleneisoindolinones **8** and permits handles for fine-tuning the electronic properties of the target compounds. The fact that the use of metal catalysts and further additives can be avoided makes our procedure particularly appealing for larger-scale synthesis in which the products, after filtering off K_2_CO_3_, are easily purified by crystallization in a high yield (see the Experimental section). Since *N*-methylated derivatives of **8**, which are also prepared by Ru(II)/Ag(I) catalysis with an excess of Cu(II) salts, are of high interest for their use in the synthesis of aristolactams,^2b^ we also investigated the transformation of **8** to **9** under the conditions of Scheme 4a.

Nicely, the target compounds were both isolated in good yields. Despite the complete conversion, the necessity of removing DMF by extraction caused a partial loss of **9** in water. To further improve the atom and step economy,^13^ a sequential one-pot cascade/β-elimination/*N*-alkylation, that is, an (a) → (b) → (c) → (d) → (e) → (f) cascade, was attempted only with the aid of K_2_CO_3_ (2 equiv) in acetonitrile. Notably, the treatment of the reaction mixture with MeI, BnBr, or allylbromide after the end of the (a) → (e) cascade process, checked by TLC, afforded **9** in excellent yields when calculated for the consecutive steps and purified directly by chromatography (Scheme 4b). In the case of **9a**, **9d**, and **9e**, only the (*Z*)-isomer was obtained, while the partial isomerization of the double bond was observed with **9b** and only to a lower extent with **9c** and **9f**. After the deprotonation of the amide, the presence of the *p*-nitro group in the sulfonyl part of **8c** probably tends to stabilize the intermediate with a single-bond character. This intermediate will be *N*-alkylated to afford the enamide with the observed *E*/*Z* ratio (Scheme 4a). The corresponding C-alkylation product was not observed. Notably, Reddy and Jeganmohan reported that the *E*/*Z* ratio of the 3-methyleneindolin-1-ones did not affect the efficiency of the subsequent Diels–Alder reaction with benzynes, which yielded aristolactams (Figure 1).^2a^

### Mechanistic Studies

The nucleophilic attack of the **2H**-derived α-halo-stabilized carbanions at the 2-acylbenzonitriles could potentially give epoxides, as discussed in Table 1. However, we have not detected any such epoxides. Instead, all isolated products can be derived from a mechanism that involves a nucleophilic attack at the nitrile group with the formation of the five-membered heterocycle in the finally obtained isoindolinone scaffolds. A computational study on the formation of 3-substituted isoindolinones in triethylamine-catalyzed reactions of nitroalkanes with *o*-cyanobenzaldehyde, which is similar to the reactions in this work, has been reported in ref (4c). Therefore, we set out to rationalize our results by DFT computations using the Gaussian 16 program^14^ at the APFD/aug-ccPVDZ level. The PCM model was used to describe the solvent (acetonitrile). The attack at the carbonyl group of 2-acetylbenzonitrile **1** by the α-halo-stabilized carbanion **2** yields diastereomeric alkoxide anions and requires the consideration of several conformers of the (*R,S*)- and (*R,R*)-configured halohydrinates **4a** (see the Supporting Information for details).^15^

**Figure 2:**
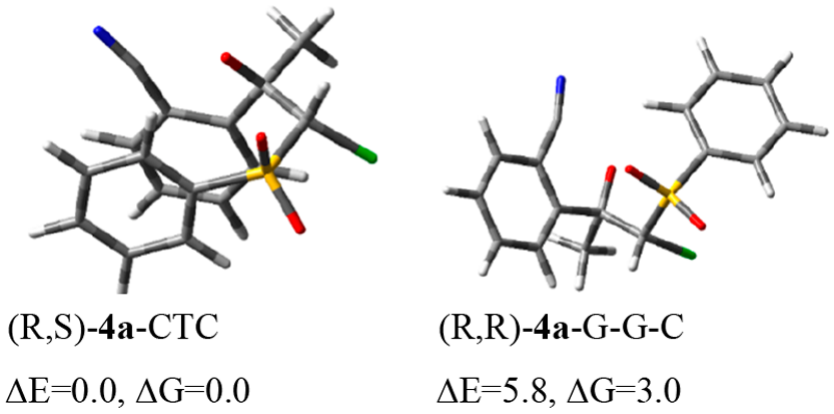
Minimum energy conformers of (*R*,*S*)- and (*R*,*R*)-configured **4a**.

**Figure 3:**
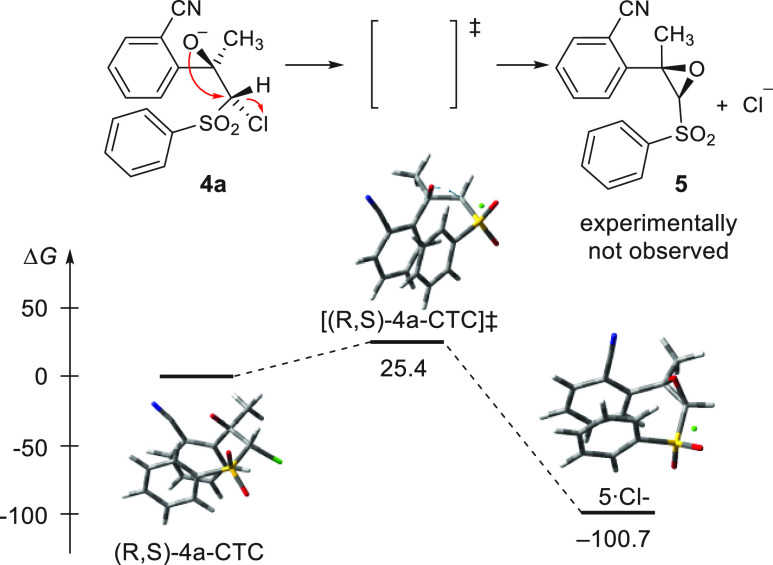
Gibbs energy profile (Δ*G*, kJ mol^–1^) for the formation of the epoxide **5**.

The lowest-energy conformer (*R*,*R*)-**4a**-G^–^G^–^C cannot lead to epoxidation. In contrast, the lowest-energy conformer (*R*,*S*)-**4a**-CTC is in a conformation that is able to form both three- and five-membered cycles (Figure 2). We thus focused on the (*R*,*S*)-**4a**-CTC conformation. The transition structure for the epoxidation from (*R*,S*)*-**4a**-CTC was readily found and could produce the epoxide at room temperature. The product would be greatly stabilized, and the reaction would be irreversible (Figure 3).

**Figure 4:**
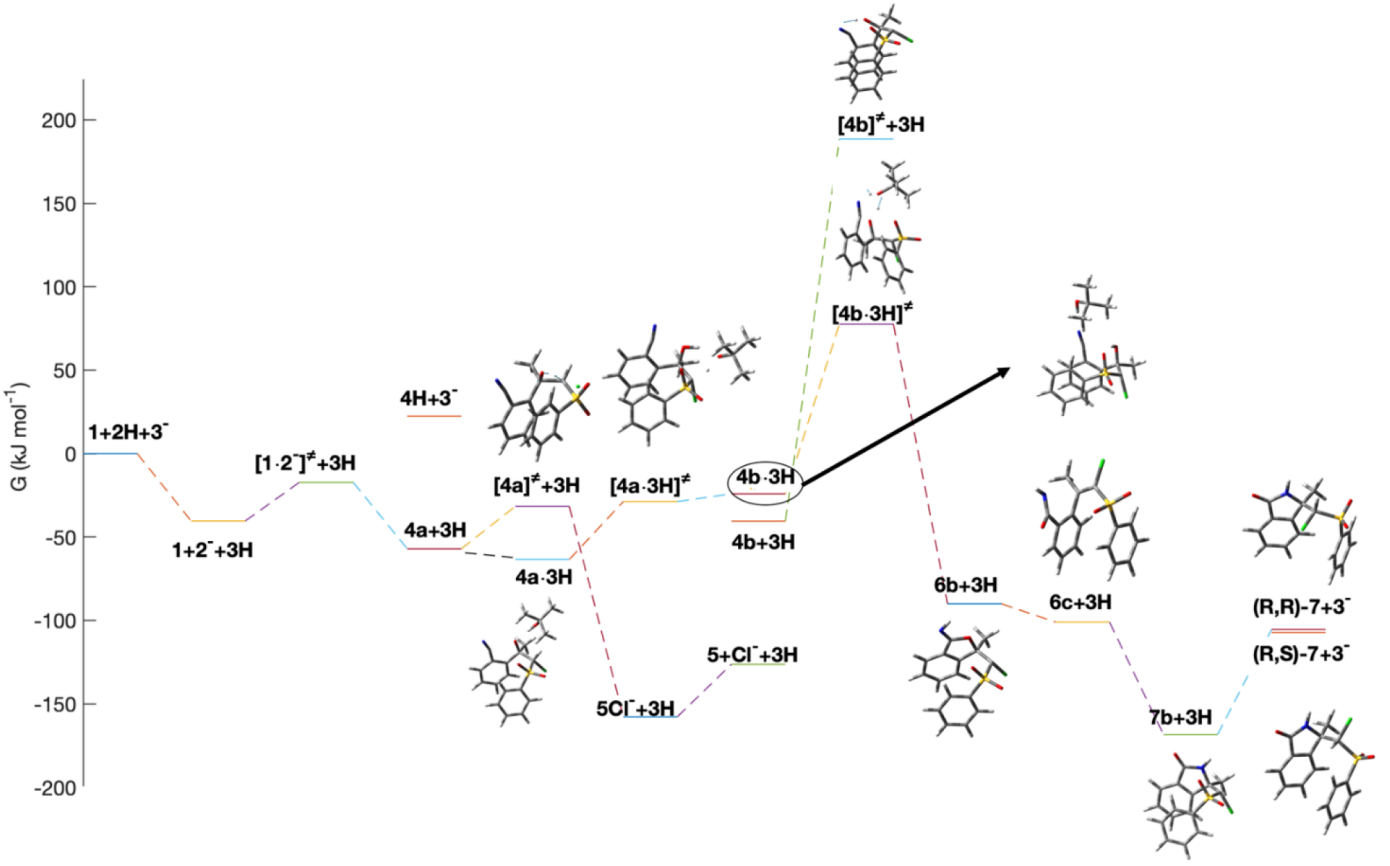
Profile of free energies of relevant species in the studied system computed at the APFD/aug-cc-pVDZ level using the PCM to describe the acetonitrile at 298 K. Species **X·3H** refer to calculated free energies for the relative complexes. Species **X + 3H** refer to free energies calculated for separated compounds.

On the other hand, a long list of trials on (*R*,*S*)-**4a**-CTC did not lead to any direct five-membered cyclization products, and the iminophthalan anion **6a** itself opens in this conformation to yield the (*R*,*S*)-**4a**-CTC conformer (see the Supporting Information for exemplary cases). We then focused on alternative routes to cyclize the lowest-energy species (*R,S*)-**4a**-CTC (Figure 4) by considering the acidic methine C–H in **4a**. *tert*-Butanol-assisted proton shuttling (Δ*G*^‡^ = 34.7 kJ mol^–1^) generates the carbanion **4b** (Δ*G* = 16.6 kJ mol^–1^), which is unable to form an epoxide and thus slows the epoxidation reaction. Notably, the further deprotonation of **4b** leads directly to the formation of the O–C bond. This reaction path, however, may only be relevant at early stages of the reaction with high base concentrations relative to the concentrations of the starting materials (Supporting Information, Table S2). More likely, another *t*BuOH-assisted proton shuttle enables ring formation and converts the halohydrinate tautomer **4b** via a thermally accessible barrier (Δ*G*^‡^ = 101.6 kJ mol^–1^) to the carbanion-substituted iminophthalan **6b** (Δ*G* = −49.5 kJ mol^–1^). Once **6b** is formed by either the monoanionic or dianionic pathway, it can rearrange to **7b** by ring-opening to the 1-chloro-1-sulfonyl-substituted alkene **6c** and a subsequent intramolecular aza-Michael reaction. The protonation of carbanion **7b** yields the isolated products **7** (Figure 4). However, no attempts to isolate the salt **7b**-K were effective since the reaction mixture appeared to be heterogeneous and the products **7** themselves were scarcely soluble in acetonitrile. Consequently, the NMR experiments performed in CD_3_CN were not indicative, while for those performed in DMSO-*d*_6_ we observed the formation of a series of unknown products as detected in entry 1 of Table 1. The complete Gibbs energy profile is reported in Figure 4.

**Scheme 5:**
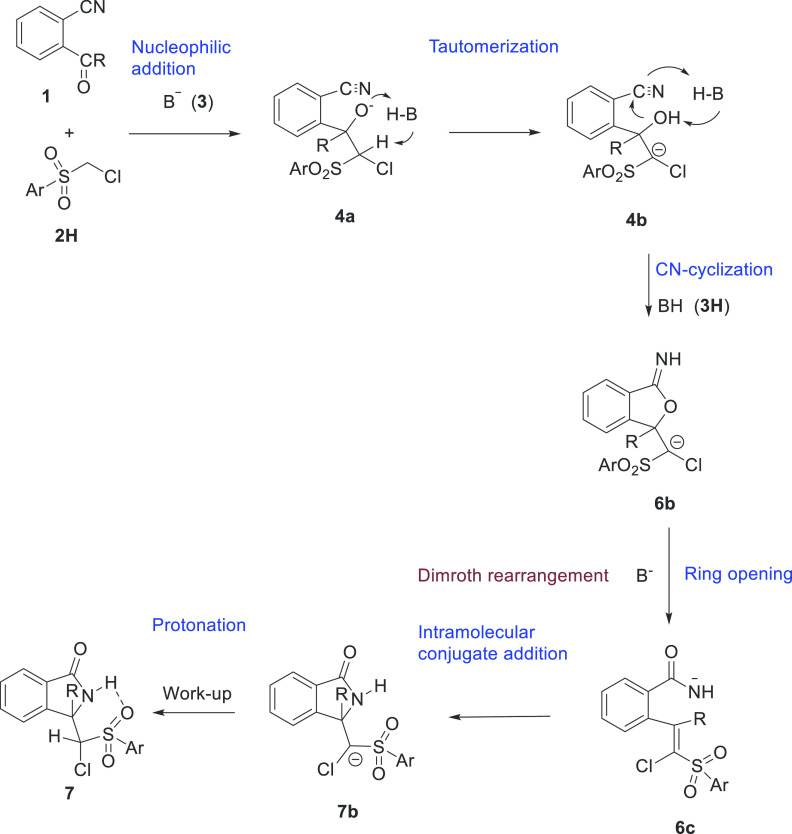
Proposed Mechanistic Pathway for the Formation of **7**

Previous reports^4,5,10b^ and DFT investigations performed herein strongly suggest a mechanism that proceeds through the carbonyl addition step of the formed chloromethylarylsulfonyl anion **2**, followed by cyclization at the cyano group of the halohydrin carbanion **4b** after a tautomeric equilibrium. Both steps, namely, tautomerization and cyclization, are favored by the proton source present as the conjugated species HB, leading to iminophthalan anion **6b** (Scheme 5). Then, the iminophthalan anion **6b** rearranges via a Dimroth-type rearrangement^4c^ to the isoindolinone motif **7**. All the steps of the mechanism are characterized by complex proton exchange equilibria; the chlorine substituent, however, is never affected until 1,2-elimination is possible, leading to stable 3-methylene-isoindolin-1-ones (Scheme 3).

## 3 Conclusions

In conclusion, herein we describe a cascade process for the synthesis of new isoindolinones bearing a tetrasubstituted carbon or (*Z*)-3-(sulfonyl-methylene)isoindolin-1-ones, which are useful luminogens materials, in good to high yields. In addition, an efficient sequential one-pot cascade/β-elimination/alkylation process was developed that was mediated only by the cheap and environmentally benign K_2_CO_3_, exclusively furnishing *N*-alkylated derivatives of (*Z*)-3-(sulfonyl-methylene)isoindolin-1-ones. These compounds represent useful intermediates in the synthesis of aristolactams. On the other hand, the possibility of utilizing strong bases like KO*t*Bu opens new synthetic opportunities for these cascade reactions since, to our knowledge, only weak bases have been used in the past as K_2_CO_3_ or Et_3_N. The mechanism and the selectivity of the described processes were analyzed and corroborated by DFT calculations.

## 4 Experimental Section

### General Methods

Unless otherwise noted, all chemicals, reagents, and solvents for the performed reactions are commercially available and were used without further purification. In particular, 2-acetylbenzonitrile, 2-formylbenzonitrile, and ((chloromethyl)sulfonyl)benzene are commercially available; all the other 2-acetylbenzonitriles, 2-formylbenzonitriles, and ((chloromethyl)sulfonyl)benzenes were prepared according to refs (5), (4h), and (7b), respectively. All the reactions were monitored by thin layer chromatography (TLC) on precoated silica gel plates (0.25 mm) and visualized by fluorescence quenching at 254 nm. Flash chromatography was carried out using silica gel 60 (70–230 mesh, Merck, Darmstadt, Germany). Yields are given for isolated products showing one spot on a TLC plate, and NMR spectra without detectable impurities.

The NMR spectra were recorded on Bruker DRX 600, 400, and 300 MHz spectrometers (^1^H 600 MHz and ^13^C 125 MHz; ^1^H 400 MHz, ^13^C 100.6 MHz, ^1^H 300 MHz, and ^13^C 75.5 MHz; ^1^H 250 MHz and ^13^C 63 MHz). The internal reference was set to the residual solvent signals (δ_H_ 7.26 ppm and δ_C_ 77.16 ppm for CDCl_3_ and δ_H_ 2.50 ppm and δ_C_ 39.52 ppm for DMSO-*d*_6_).^19^ The ^13^C NMR spectra were recorded under broad-band proton-decoupling. The following abbreviations are used to indicate the multiplicity in NMR spectra: s = singlet, d = doublet, t = triplet, q = quartet, dd = doublet of doublets, m = multiplet, brs = broad signal. Coupling constants (*J*) are given in Hertz.

High-resolution mass spectra (HRMS) were acquired using a Bruker SolariX XR Fourier transform ion cyclotron resonance mass spectrometer (Bruker Daltonik GmbH, Bremen, Germany) equipped with a 7T refrigerated actively shielded superconducting magnet. At LMU München, high-resolution mass spectra (HRMS) were recorded on a Finnigan MAT 90 system, a Finnigan MAT 95 system, a Thermo Finnigan LTQ FT Ultra Fourier Transform ion cyclotron resonance system, or a Q Exactive GC Orbitrap GC/MS. For ionization of the samples, either electron-impact ionization (EI) or electrospray ionization (ESI) was applied.

### General Procedure for the Synthesis of 3,3-Disubstituted Isoindolinones with Substituted ((Chloromethyl)sulfonyl)benzene

### Procedure with Potassium Carbonate

2-Acetylbenzonitriles **1** (0.137 mmol, 1.0 equiv) were added to a solution of substituted ((chloromethyl)sulfonyl)benzenes **2H** (0.164 mmol, 1.2 equiv) and potassium carbonate (0.137 mmol, 19 mg, 1.0 equiv) in anhydrous CH_3_CN (0.45 M, 0.30 mL) at 50 °C in an oil bath. The reaction mixture was stirred at the same temperature for 24 h, then diluted with DCM, and the solids filtered off. The solution was evaporated to afford the crude product as white solid, which was purified by column chromatography (hexane/ethyl acetate = 80:20) to provide **7b**, **7c**, and **7e**–**7h** (60–92%).

### Procedure with Potassium *tert*-Butoxide

2-Acetylbenzonitriles **1** (0.137 mmol, 1.0 equiv) were added to a solution of substituted ((chloromethyl)sulfonyl)benzenes **2H** (0.164 mmol, 1.2 equiv) and potassium *tert*-butoxide (0.137 mmol, 15 mg, 1.0 equiv) in anhydrous CH_3_CN (0.45 M, 0.30 mL) at r.t. The reaction mixture was directly purified by column chromatography (hexane/ethyl acetate = 80:20) to provide **7a** and **7d** (64–86%).

#### 3-(Chloro(phenylsulfonyl)methyl)-3-methylisoindolin-1-one (**7a**)

White solid (86%, 40 mg). Mixture of diastereomers, d.r. = 91:9. Recrystallization of **4a** (20 mg) from a hexane/EtOAc (2/1) mixture at −20 °C yielded crystals that were suitable for X-ray single-crystal structure determination.^11^

^1^H NMR (400 MHz, CDCl_3_) δ 7.94 (d, *J* = 8.6 Hz, 2H), 7.85 (d, *J* = 7.5 Hz, 1H), 7.71 (t, *J* = 7.1 Hz, 1H), 7.59 (t, *J* = 7.3 Hz, 3H), 7.50 (t, *J* = 7.5 Hz, 1H), 7.39 (d, *J* = 7.6 Hz, 1H), 7.02 (brs, 1H), 5.10 (s, 1H, *major*), 4.49 (s, 1H, *minor*), 2.09 (s, 3H, *major*), 1.97 (s, 3H, *minor*). ^13^C{^1^H} NMR (75.5 MHz, DMSO-*d*_6_) δ 168.8, 149.4, 137.6, 134.8, 132.3, 131.1, 129.3, 129.1, 129.0, 123.0, 122.3, 75.9, 63.2, 24.8. ESI-HRMS: found *m*/*z* 358.0273 Calcd for C_16_H_14_^35^ClNNaO_3_S^+^: (M + Na)^+^ 358.0275.

#### 4-((Chloro(1-methyl-3-oxoisoindolin-1-yl)methyl)sulfonyl)benzonitrile (**7b**)

Yellow solid (91%, 45 mg). Single diastereoisomer. Mp 196–197 °C (from hexane/ethyl acetate).

^1^H NMR (300 MHz, DMSO-*d*_6_): δ 8.57 (s, 1H), 8.18 (d, *J* = 8.5 Hz, 2H), 8.10 (d, *J* = 8.5 Hz, 2H), 7.73–7.60 (m, 3H), 7.51 (t, *J* = 7.2 Hz, 1H), 6.39 (s, 1H), 1.87 (s, 3H). ^13^C{^1^H} NMR (75.5 MHz, DMSO-*d*_6_) δ 168.8, 148.9, 141.6, 133.4, 132.3, 131.2, 129.6, 129.2, 123.0, 122.3, 117.4, 116.9, 76.0, 63.1, 24.8. EI-HRMS: found *m*/*z* 361.0397. Calcd for C_17_H_14_^35^ClN_2_O_3_S^+^: (M + H)^+^ 361.0408.

#### 3-(Chloro((4-nitrophenyl)sulfonyl)methyl)-3-methylisoindolin-1-one (**7c**)

White solid (63%, 33 mg). Mixture of diastereoisomers, d.r. = 79:21.

^1^H NMR (400 MHz, CDCl_3_) δ 8.43 (d, *J* = 8.8 Hz, 2H, *major*), 8.15 (d, *J* = 8.8 Hz, 2H, *major + minor*), 7.87 (d, *J* = 7.5 Hz, 2H, *minor*), 7.73 (d, *J* = 7.5 Hz, 1H), 7.63–7.59 (m, 1H), 7.55–7.52 (m, 1H), 7.40 (d, *J* = 7.6 Hz, 1H), 6.93 (s, 1H, *major + minor*), 5.15 (s, 1H, *major*), 4.56 (s, 1H, *minor*), 2.11 (s, 3H, *major*), 1.97 (s, 3H, *minor*). ^13^C{^1^H} NMR (100.6 MHz, DMSO-*d*_6_) δ 168.8, 150.9, 148.9, 142.9, 132.3, 131.2, 130.7, 129.2, 124.6, 123.1, 122.4, 76.0, 63.1, 24.8. ESI-HRMS: found *m*/*z* 381.0308. Calcd for C_16_H_14_^35^ClN_2_O_5_S^+^: (M + H)^+^ 381.0306.

#### 3-(Chloro((4-methoxyphenyl)sulfonyl)methyl)-3-methylisoindolin-1-one (**7d**)

White solid (64%, 32 mg). Mixture of diastereoisomers, d.r. = 66:34.

^1^H NMR (300 MHz, CDCl_3_) δ 7.90–7.83 (m, 5H, *major + minor*), 7.74 (d, *J* = 7.1 Hz, 1H), 7.61–7.47 (m, 5H, *major + minor*), 7.38 (d, *J* = 7.6 Hz, 1H, *major*), 7.06–6.99 (m, 5H), 5.06 (s, 1H, *major*), 4.44 (s, 1H, *minor*), 3.89 (s, 3H, *major*), 3.88 (s, 3H, *minor*), 2.07 (s, 3H, *major*), 1.95 (s, 3H, *minor*). ^13^C{^1^H} NMR (75.5 MHz, CDCl_3_) δ 169.6, 168.5, 164.9 (2C), 149.2, 148.3, 132.7, 132.2, 132.0, 131.3, 130.9, 129.6, 129.5, 128.3, 127.9, 124.9, 124.5, 124.3, 120.6, 114.7, 114.5, 79.0, 63.7, 55.9, 29.8, 29.5, 24.8, 20.7, 14.3. ESI-HRMS: found *m*/*z* 366.0563. Calcd for C_17_H_17_^35^ClNO_4_S^+^: (M + H)^+^ 366.0561.

#### 4-((Chloro(5-chloro-1-methyl-3-oxoisoindolin-1-yl)methyl)sulfonyl)benzonitrile (**7e**)

White solid (74%, 40 mg). Mixture of diastereoisomers, d.r. = 56:44.

^1^H NMR (400 MHz, CDCl_3_) δ 8.11–8.05 (m, 3H, *major + minor*), 7.91–7.88 (m, 3H, *major + minor*), 7.83–7.82 (m, 1H, *minor*),δ 7.66 (d, *J* = 7.8 Hz, 1H), 7.58–7.53 (m, 2H, *major + minor*), 7.39(s, 1H, *minor*), δ 7.34 (d, *J* = 7.7 Hz, 1H, *major*), 6.98 (s, 1H, *major*), 5.09 (s, 1H, *major*), 4.52 (s, 1H, *minor*), 2.09 (s, 3H, *major*), 1.96 (s, 3H, *minor*). ^13^C{^1^H} NMR (125 MHz, CDCl_3_) δ 167.9, 166.9, 146.4, 145.8, 140.7, 136.6, 136.3, 133.2, 133.1, 133.1, 133.1, 132.7, 132.6, 130.4, 130.4, 126.1, 124.9, 124.6, 122.0, 119.0, 118.9, 116.9, 116.8, 115.0, 78.3, 63.7, 63.5, 24.6, 21.0. ESI-HRMS: found *m*/*z* 392.9874. Calcd for C_17_H_11_^35^Cl_2_N_2_O_3_S^–^: (M)^−^ 392.9873.

#### 4-((Chloro(1-hexyl-3-oxoisoindolin-1-yl)methyl)sulfonyl)benzonitrile (**7f**)

Yellow solid (60%, 35.3 mg). Mixture of diastereoisomers, d.r. = 55:45.

^1^H NMR (400 MHz, CDCl_3_) δ 8.06–8.03 (m, 3H, *major + minor*), 7.88–7.85 (m, 4H, *major + minor*), 7.67 (d, *J* = 7.5 Hz, 1H, *minor*), 7.59- 7.52 (m, 3H, *major + minor*), 7.35 (d, *J* = 7.5 Hz, 1H), 7.16 (s, 1H, *minor*), 6.81 (s, 1H, *major*), 5.15 (s, 1H, *major*), 4.57 (s, 1H, *minor*), 2.70–2.63 (m, 1H), 2.49–2.43 (m, 1H), 2.38–2.31 (m, 1H), 1.19–1.14 (m, 9H, *major + minor*), 0.84–0.80 (m, 5H, *major + minor*). ^13^C{^1^H} NMR (75.5 MHz, CDCl_3_) δ 169.9, 169.0, 146.2, 145.3, 141.2, 133.1, 132.9, 132.8, 132.5, 132.3, 131.9, 130.4, 130.3, 129.9, 129.7, 124.9, 124.5, 120.9, 118.8, 118.6, 116.9, 117.0, 79.3, 67.5, 36.1, 32.1, 29.8, 29.2, 29.1, 24.0, 22.8, 22.6, 14.1. ESI-HRMS: found *m*/*z* 431.1196. Calcd for C_22_H_24_^35^ClN_2_O_3_S^+^: (M + H)^+^ 431.1191.

#### 4-(((5-Bromo-1-methyl-3-oxoisoindolin-1-yl)chloromethyl)sulfonyl)benzonitrile (**7g**)

Yellow solid (89%, 53 mg). Mixture of diastereoisomers, d.r. = 58:42.

^1^H NMR (400 MHz, CDCl_3_) δ 8.10–8.05 (m, 2H, *major + minor*), 7.99 (s, 1H, *major*), 7.90–7.88 (m, 2H, *major + minor*), 7.73–7.69 (m, 1H), 7.60 (d, *J* = 8.1 Hz, 1H, *minor*),7.50–7.45 (m, 1H), 7.37 (s, 1H), 7.29 (s, 1H), 6.96 (s, 1H, *major*), 5.08 (s, 1H, *major*), 4.52 (s, 1H, *minor*), 2.08 (s, 3H, *major*), 1.95 (s, 3H, *minor*). ^13^C{^1^H} NMR (100.6 MHz, DMSO-*d*_6_) δ 167.2 (*major*), 166.8 (*minor*), 147.8 (*major*), 146.4 (*minor)*, 141.5 (*major*), 141.1 (*minor*) 135.02 (*major*),134.6 (*minor*), 133.7, 133.5 (*major*), 133.3 (*minor*), 129.6, 125.7, 124.7, 122.5, 117.4, 117.0, 77.4, 75.6, 63.1 (*major*), 62.8 (*minor*), 26.4 (*minor)*, 24.5 (*major*). ESI-HRMS: found *m*/*z* 438.9517. Calcd for C_17_H_13_^79^Br^35^ClN_2_O_3_S^+^: (M + H)^+^ 438.9513.

#### 6-Bromo-3-(chloro((4-nitrophenyl)sulfonyl)methyl)-3-methylisoindolin-1-one (**7h**)

White solid (84%, 53 mg). Mixture of diastereoisomers, d.r. = 76:24

^1^H NMR (400 MHz, DMSO-*d*_6_) δ 9.21 (s, 1H, *minor*), 8.89 (s, 1H, *major*), 8.49 (d, *J* = 9.0 Hz, 2H, *major*), 8.39 (d, *J* = 8.5 Hz, 2H, *minor*), 8.19 (d, *J* = 9.2 Hz, 2H, *major*), 7.89–7.84 (m, 2H), 7.80–7.76 (m, 1H), 7.71–7.69 (m, 1H, *major*), 7.61 (d, *J* = 8.6 Hz, 1H, *minor*), 6.62 (s, 1H,, *minor*), 6.46 (s, 1H,*major*), 1.87 (s, 3H, *major*), 1.61 (s, 3H, *minor*). ^13^C{^1^H} NMR (100.6 MHz, DMSO-*d*_6_) δ 167.2 (*major*), 166.8 (*minor*), 150.9 (*major*), 150.6 (*minor*), 147.8, 142.7 (*major)*, 142.4 (*minor*), 135.1, 133.7, 130.6, 125.8, 124.8, 124.6, 122.5, 77.4 (*minor*), 75.6 (*major*), 63.1 (*major*), 62.8 (*minor*), 26.4 (*minor*), 24.5 (*major*). ESI-HRMS: found *m*/*z* 492.9026. Calcd for C_16_H_12_^79^Br^35^Cl_2_N_2_O_5_S^–^: (M + Cl)^−^ 492.9035.

### General Procedure for the Synthesis of 3-Methylene-isoindolin-1-ones (**8**)

2-Formylbenzonitriles (0.137 mmol, 1.0 equiv) were added to a solution of ((chloromethyl)sulfonyl)benzenes **2H** (0.164 mmol, 1.2 equiv) and potassium carbonate (0.137 mmol, 19 mg, 1.0 equiv) in anhydrous CH_3_CN (0.45 M, 0.30 mL) at 50 °C in an oil bath. The reaction mixture was stirred at the same temperature for 24 h, diluted with DCM, then filtered off. The filtrate was evaporated to afford the crude product as white solid, which was purified by column chromatography (hexane/ethyl acetate = 80/20) to provide **8a**–**h** (54–99%).

The reaction was scaled up to 1.37 mmol (180 mg) of 2-formyl benzonitrile according to the above procedure. After 24 h, the reaction mixture was diluted with DCM and filtered off. After evaporation of the solvent, the title compound was purified by crystallization (13 mL, CHCl_3_/hexane = 1:1 at −20 °C) to obtain **8a** as pure solid in a 99% yield (387 mg).

#### (*Z*)-3-((Phenylsulfonyl)methylene)isoindolin-1-one (**8a**)

White solid (99%, 39 mg). Mp 181–182 °C (from hexane/ethyl acetate).

^1^H NMR (400 MHz, DMSO-*d*_6_) δ 10.43 (s, 1H), 8.07 (d, *J* = 7.7 Hz, 3H), 7.83–7.64 (m, 6H), 6.95 (s, 1H). Data were found to be in agreement with literature.^3b^

#### (*Z*)-4-(((3-Oxoisoindolin-1-ylidene)methyl)sulfonyl)benzonitrile (**8b**)

White solid (99%, 48.9 mg). Mp 229–230 °C (from hexane/ethyl acetate).

^1^H NMR (400 MHz, CDCl_3_) δ 9.39 (s, 1H), 8.09 (d, *J* = 8.5 Hz, 2H), 7.92–7.90 (m, 1H), 7.87 (d, *J* = 8.4 Hz, 2H), 7.70–7.65 (m, 2H), 7.61–7.59 (m, 1H), 6.03 (s, 1H). ^13^C{^1^H} NMR (75.5 MHz, DMSO-*d*_6_) δ 167.9, 145.8, 145.1, 135.9, 133.8, 133.5, 132.6, 128.2, 127.7, 123.5, 122.6, 117.64, 115.9, 99.9. EI-HRMS: found *m*/*z* 361.0397. Calcd for C_16_H_10_N_2_O_3_S^**•**+^: (M)^•+^ 361.0408.

#### (*Z*)-3-(((4-Nitrophenyl)sulfonyl)methylene)isoindolin-1-one (**8c**)

White solid (99%, 44 mg). Mp 198–199 °C (from hexane/ethyl acetate).

^1^H NMR (400 MHz, CDCl_3_) δ 9.39 (s, 1H), 8.41 (d, *J* = 8.6 Hz, 2H), 8.17 (d, *J* = 8.6 Hz, 2H), 7.93–7.91 (m, 1H), 7.70–7.67 (m, 2H), 7.62–7.59 (m, 1H), 6.05 (s, 1H). ^13^C{^1^H} NMR (75.5 MHz, DMSO-*d*_6_) δ 167.9, 150.3, 147.2, 145.3, 135.9, 133.5, 132.7, 128.5, 128.3, 124.9, 123.5, 122.6, 99.7. EI-HRMS: found *m*/*z* 330.0301. Calcd for C_15_H_10_N_2_O_5_S^•+^: (M)^•+^ 330.0305.

#### (*Z*)-3-(((4-Methoxyphenyl)sulfonyl)methylene)isoindolin-1-one (**8d**)

White solid (70%, 30 mg). Mp 200–201 °C (from hexane/ethyl acetate).

^1^H NMR (400 MHz, CDCl_3_) δ 9.43 (s, 1H), 7.88 (d, *J* = 8.8 Hz, 3H), 7.63 (dd, *J* = 5.5, 3.2 Hz, 2H), 7.59–7.56 (m, 1H), 7.01 (d, *J* = 8.9 Hz, 2H), 6.08 (s, 1H), 3.87 (s, 3H). ^13^C{^1^H} NMR (75.5 MHz, CDCl_3_) δ 167.5, 164.0, 143.0, 136.0, 133.2 132.3, 129.5, 129.1, 124.4, 121.4, 114.8, 101.2, 55.9. EI-HRMS: found *m*/*z* 315.0560. Calcd for C_16_H_13_NO_4_S^•+^: (M)^•+^ 315.0560.

#### (*Z*)-6-Chloro-3-(((4-nitrophenyl)sulfonyl)methylene)isoindolin-1-one (**8e**)

White solid (72%, 36 mg). Mp 234–235 °C (from hexane/ethyl acetate).

^1^H NMR (300 MHz, CDCl_3_) δ 9.44 (s, 1H), 8.42 (d, *J* = 8.9 Hz, 2H), 8.16 (d, *J* = 8.9 Hz, 2H), 7.88 (s, 1H), 7.66–7.62 (m, 1H), 7.53 (d, *J* = 8.0 Hz, 1H), 6.03 (s, 1H). ^13^C{^1^H} NMR (75.5 MHz, DMSO-*d*_6_) δ 166.8, 150.3, 147.0, 144.4, 137.4, 134.6, 133.4, 130.3, 128.6, 124.9, 124.4, 123.4, 100.7. EI-HRMS: found *m*/*z* 363.9914. Calcd for C_15_H_9_^35^ClN_2_O_5_S^•+^: (M)^•+^ 363.9915.

#### (*Z*)-4-(((5-Chloro-3-oxoisoindolin-1-ylidene)methyl)sulfonyl)benzonitrile (**8f**)

White solid (99%, 46 mg). Mp 219–220 °C (from hexane/ethyl acetate).

^1^H NMR (400 MHz, CDCl_3_) δ 9.43 (s, 1H), 8.08 (d, *J* = 8.2 Hz, 2H), 7.87 (d, *J* = 8.2 Hz, 1H), 7.64 (d, *J* = 7.9 Hz, 1H), 7.53 (d, *J* = 7.9 Hz, 1H), 6.01 (s, 1H). ^13^C{^1^H} NMR (63 MHz, DMSO-*d*_6_) δ 166.7, 145.6, 144.2, 137.4, 134.6, 133.8, 133.4, 130.3, 127.7, 124.4, 123.4, 117.6, 116.0, 100.8. EI-HRMS: found *m*/*z* 344.0021. Calcd for C_16_H_9_^35^ClN_2_O_3_S^•+^: (M)^•+^ 344.0017.

#### (*Z*)-4-(((5-Bromo-3-oxoisoindolin-1-ylidene)methyl)sulfonyl)benzonitrile (**8g**)

White solid (75%, 40 mg). Mp 194–195 °C (from hexane/ethyl acetate).

^1^H NMR (300 MHzDMSO-*d*_6_) δ 10.75 (s, 1H), 8.25 (d, *J* = 8.2 Hz, 2H), 8.17 (d, *J* = 8.2 Hz, 2H), 8.00–7.95 (m, 3H), 7.05 (s, 1H). ^13^C{^1^H} NMR (100.6 MHz, DMSO-*d*_6_) δ 166.7, 145.7, 144.4, 136.2, 135.1, 133.8, 130.4, 127.7, 126.29, 125.9, 124.6, 117.7, 116.0, 100.8. EI-HRMS: found *m*/*z* 387.9515. Calcd for C_16_H_9_^79^BrN_2_O_3_S^•+^: (M)^•+^ 387.9512.

#### (*Z*)-6-Bromo-3-(((4-nitrophenyl)sulfonyl)methylene)isoindolin-1-one (**8h**)

Yellow solid (54%, 30 mg). Mp 227–228 °C (from hexane/ethyl acetate).

^1^H NMR (400 MHz, DMSO-*d*_6_) δ 10.78 (s, 1H), 8.47 (d, *J* = 8.5 Hz, 2H), 8.33 (d, *J* = 8.5 Hz, 2H), 8.03–7.96 (m, 3H), 7.08 (s, 1H). ^13^C{^1^H} NMR (75.5 MHz, DMSO-*d*_6_) δ 166.7, 150.3, 147.0, 144.5, 136.3, 135.0, 130.4, 128.6, 126.3, 126.0, 125.0, 124.6, 100.7. EI-HRMS: found *m*/*z* 407.9402. Calcd for C_15_H_9_^79^BrN_2_O_5_S^•+^: (M)^•+^ 407.9410.

### General Procedure for the *N*-Methylation of (*Z*)-3-((Phenylsulfonyl)methylene)isoindolin-1-ones

To a solution of **8a** or **8c** (0.14 mmol, 1.0 equiv) in anhydrous DMF (0.30 M, 0.47 mL) was added potassium carbonate (0.21 mmol, 29.0 mg, 1.5 equiv) and CH_3_I (0.21 mmol, 0.013 mL, 1.5 equiv). The reaction mixture was allowed to stir at room temperature for 18 h, then diluted with ethyl acetate and washed with water (3 × 5 mL) to obtain the crude product as white solid, which was purified by flash column chromatography (hexane/ethyl acetate = 80:20) to provide **9a** (62%) and **9b** (66%, *Z*/*E* = 68:32).

#### (*Z*)-2-Methyl-3-((phenylsulfonyl)methylene)isoindolin-1-one (**9a**)

White solid (62%, 26 mg), Mp 154–155 °C (from hexane/ethyl acetate).

^1^H NMR (400 MHz, CDCl_3_) δ 8.03 (d, *J* = 7.6 Hz, 2H), 7.84–7.82 (m, 1H), 7.67–7.65 (m, 1H) 7.61–7.56 (m, 5H), 6.35 (s, 1H), 3.66 (s, 3H). ^13^C{^1^H} NMR (100.6 MHz, CDCl_3_) δ 168.3, 143.8, 143.0, 136.7, 133.7, 133.1, 131.7, 129.6, 128.0, 127.2, 124.2, 120.4, 104.0, 30.5. ESI-HRMS: found *m*/*z* 300.0690 Calcd for C_16_H_14_N_3_OS^+^: (M + H)^+^ 300.0689.

#### 2-Methyl-3-(((4-nitrophenyl)sulfonyl)methylene)isoindolin-1-one (**9b**)

White solid (66%, 32 mg), mixture of isomers, *Z*/*E* = 68:32

^1^H NMR (300 MHz, CDCl_3_) δ 8.78 (d, *J* = 7.3 Hz, 1H, *(E)-isomer*), 8.45–8.38 (m, 3H, *(Z)- and (E)-isomers*), 8.24–8.21 (m, 2H, *(Z)- and (E)-isomers*), 7.88–7.86 (m, 2H, *(E)-isomer*), 7.70–7.58 (m, 4H, *(Z)- and (E)-isomers*), 6.27 (s, 1H, *(Z)-isomer*), 6.08 (s, 1H, *(E)-isomer*), 3.64 (s, 3H, *(Z)-isomer*), 3.22 (s, 3H, *(E)-isomer*). ^13^C{^1^H} NMR (75.5 MHz, CDCl_3_) δ 168.2 (*(Z)-isomer*), 166.6 (*(E)-isomer*), 150.7, 149.1(*(E)-isomer*), 148.7 (*(Z)-isomer*), 148.2, 145.5, 136.4, 133.7(*(E)-isomer*), 133.4 (*(Z)-isomer*), 132.5 (*(E)-isomer*), 132.2 (*(Z)-isomer*), 131.9 (*(Z)-isomer*), 130.1, 128.7 (*(Z)-isomer*), 128.3 (*(E)-isomer*), 127.8, 124.9 (*(Z)-isomer*), 124.8, 124.5 (*(E)-isomer*), 124.1, 120.5, 105.9, 101.6, 30.6 (*(Z)-isomer*), 26.7 (*(E)-isomer*). ESI-HRMS: found *m*/*z* 345.0541. Calcd for C_16_H_13_N_2_O_5_S^+^: (M + H)^+^ 345.0531.

### One-Pot *N*-Alkylation of (*Z*)-3-((Phenylsulfonyl)methylene)isoindolin-1-one

2-Formylbenzonitrile (0.14 mmol, 1.0 equiv) was added to a solution of **2H** (0.14 mmol, 1.0 equiv) and potassium carbonate (0.28 mmol, 2.0 equiv) in anhydrous CH_3_CN (0.45 M) at 50 °C in an oil bath. The reaction mixture was allowed to stir at the same temperature for 24 h, cooled at room temperature, and treated with CH_3_I or BnBr (0.21 mmol, 1.5 equiv). The reaction was monitored by TLC until the maximum conversion was reached. After 18 h, the crude reaction was diluted with DCM, the solids were filtered off, and the solution was evaporated, affording the crude product as a white solid. Purification by flash column chromatography (hexane/ethyl acetate = 70:30) provided **9a** (88%) and **9c**–**9f**.

#### (*Z*)-2-Benzyl-3-((phenylsulfonyl)methylene)isoindolin-1-one (**9c**)

White solid (90%, 47 mg). Mixture of isomers, *Z/E* = 92:8

^1^H NMR (400 MHz, DMSO-*d*_6_) δ 8.25 (d, *J* = 7.7 Hz, 1H), 7.85 (d, *J* = 7.4 Hz, 1H), 7.78 (t, *J* = 7.5 Hz, 1H), 7.71 (t, *J* = 7.4 Hz, 1H), 7.67 (d, *J* = 7.1 Hz, 2H), 7.62–7.59 (m, 1H), 7.46 (t, *J* = 7.7 Hz, 2H), 7.21–7.19 (m, 3H), 7.05 (s, 1H), 6.98 (dd, *J* = 7.4, 2.2 Hz, 1H), 5.57 (s, 1H, *(E)-isomer*), 5.54 (s, 2H, *(Z)-isomer*). ^13^C{^1^H} NMR (100.6 MHz, DMSO-*d*_6_) δ 168.3, 141.9, 141.8, 137.4, 137.2, 133.9, 133.7, 132.3, 129.5, 128.5, 126.9, 126.8, 126.7, 125.8, 123.8, 122.2, 104.8, 45.8. ESI-HRMS: found *m*/*z* 376.1024 Calcd for C_22_H_18_NO_3_S^+^: (M + H)^+^ 376.1002.

#### (*Z*)-2-Allyl-3-((phenylsulfonyl)methylene)isoindolin-1-one (**9d**)

White solid (88%, 40 mg). Mp 139–141 °C (petroleum ether/ethyl acetate).

^1^H NMR (300 MHz, CDCl_3_) δ 8.00 (d, *J* = 7.4 Hz, 2H), 7.85–7.83 (m, 1H), 7.60–7.54 (m, 6H), 6.30 (s, 1H), 5.90–5.78 (m, 1H), 5.04–4.93 (m, 4H). ^13^C{^1^H} NMR (75.5 MHz, CDCl_3_) δ 168.3, 142.5, 142.3, 137.1, 133.6, 133.2, 132.4, 131.8, 129.5, 127.8, 127.3, 124.2, 120.5, 116.2, 103.7, 45.1. ESI-HRMS: found *m*/*z* 326.0845 Calcd for C_18_H_16_NO_3_S^+^: (M+ H)^+^ 326.0846.

#### (*Z*)-4-(((2-Allyl-3-oxoisoindolin-1-ylidene)methyl)sulfonyl)benzonitrile (**9e**)

White solid (98%, 48 mg). Mp 156–158 °C (petroleum ether/ethyl acetate).

^1^H NMR (400 MHz, CDCl_3_) δ 8.11 (d, *J* = 8.4 Hz, 2H), 7.85 (d, *J* = 8.3 Hz, 3H), 7.66–7.59 (m, 3H), 6.22 (s, 1H), 5.86–5.76 (m, 1H), 5.01–4.88 (m, 4H). ^13^C{^1^H} NMR (100.6 MHz, CDCl_3_) δ 168.2, 146.6, 143.9, 136.9, 133.4, 133.2 (×2), 132.4, 132.2, 128.0, 127.6, 124.5, 120.6, 117.2, 116.1, 101.5, 45.0. MALDI-HRMS: found *m*/*z* 351.0803. Calcd for C_19_H_15_N_2_O_3_S^+^: (M + H)^+^ 351.0798.

#### (*Z*)-2-Benzyl-6-chloro-3-(((4-nitrophenyl)sulfonyl)methylene)isoindolin-1-one (**9f**)

White solid (60%, 38 mg). mixture of isomers, *Z*/*E* = 78:22

^1^H NMR (400 MHz, CDCl_3_) δ 8.42 (d, *J* = 8.8 Hz, 1H, *minor*), 8.16 (d, *J* = 8.9 Hz, 1H, *minor*), 8.03 (d, *J* = 8.9 Hz, 2H, *major + minor*), 7.90 (s, 1H), 7.68 (d, *J* = 1.9 Hz, 1H, *minor*), 7.66 (d, *J* = 1.8 Hz, 1H, *major*), 7.61 (t, *J* = 8.8 Hz, 3H, *major + minor*), 7.53 (d, *J* = 8.3 Hz, 1H, *minor)*, 7.17 (d, *J* = 7.4 Hz, 2H), 6.90 (d, *J* = 6.6 Hz, 2H), 6.25 (s, 1H, *major*), 6.03 (s, 1H, *minor*), 5.65 (s, 2H, *major + minor*). ^13^C{^1^H} NMR (100.6 MHz, DMSO-*d*_6_) δ 166.8, 149.8, 146.8, 142.7, 137.2, 136.7, 135.6, 133.7, 128.6, 128.3, 128.0, 126.5, 125.3, 124.4, 124.3, 123.6, 103.7, 45.5. MALDI-HRMS: found *m*/*z* 477.0295 Calcd for C_22_H_15_ClN_2_NaO_5_S^+^: (M + Na)^+^ 477.0282.

## Supporting Information Available

^1^H and ^13^C{^1^H} NMR spectra, computational details, Gibbs free energies of transition states, Cartesian coordinates, electronic energies, and X-ray diffraction data (PDF)
FAIR data, including the primary NMR FID files, for compounds **7a**–**7h**, **8a**–**8h**, and **9a**–**9f** (ZIP)

## Accession Codes

CCDC 2087404 contains the supplementary crystallographic data for this paper. These data can be obtained free of charge via www.ccdc.cam.ac.uk/data_request/cif, or by emailing data_request@ccdc.cam.ac.uk, or by contacting The Cambridge Crystallographic Data Centre, 12 Union Road, Cambridge CB2 1EZ, UK; fax: +44 1223 336033.

## References

[ref1] aSpeck K.; Magauer T. The chemistry of isoindole natural products. Beilstein J. Org. Chem. 2013, 9, 2048–2078. 10.3762/bjoc.9.243. 24204418 PMC3817534

[ref2] aReddy M. C.; Jeganmohan M. Total synthesis of aristolactam alkaloids via synergistic C–H bond activation and dehydro-Diels–Alder reactions. Chem. Sci. 2017, 8, 4130–4135. 10.1039/C7SC00161D. 30155216 PMC6100235

[ref3] aSavela R.; Mendez-Galvez C. Isoindolinone Synthesis via One-Pot Type Transition Metal Catalyzed C–C Bond Forming Reactions. Chem. - Eur. J. 2021, 27, 5344–5378. 10.1002/chem.202004375. 33125790 PMC8048987

[ref4] aSong Y. S.; Lee C. H.; Lee K.-J. Application of Baylis-Hillman Methodology in a New Synthesis of 3-Oxo-2,3-dihydro-1*H*-isoindoles. J. Heterocycl. Chem. 2003, 40, 939–941. 10.1002/jhet.5570400532.

[ref5] Di Mola A.; Di Martino M.; Capaccio V.; Pierri G.; Palombi L.; Tedesco C.; Massa A. Synthesis of 2-Acetylbenzonitriles and Their Reactivity in Tandem Reactions with Carbon and Hetero Nucleophiles: Easy Access to 3,3-Disubstituted Isoindolinones. Eur. J. Org. Chem. 2018, 1699–1708. 10.1002/ejoc.201800240.

[ref6] aNishimura T.; Noishiki A.; Ebe Y.; Hayashi T. Hydroxorhodium/Chiral Diene Complexes as Effective Catalysts for the Asymmetric Arylation of 3-Aryl-3-hydroxyisoindolin-1-ones. Angew. Chem., Int. Ed. 2013, 52, 1777–1780. 10.1002/anie.201208593. 23307761

[ref7] aMayr H., Ofial A. R. https://www.cup.uni-muenchen.de/oc/mayr/DBintro.html (accessed on 2021-06-30).

[ref8] aMakosza M. Nucleophilic substitution of hydrogen in electron-deficient arenes, a general process of great practical value. Chem. Soc. Rev. 2010, 39, 2855–2868. 10.1039/b822559c. 20505851

[ref9] aDarzens G. Méthode générale de synthèse des aldéhydes à l’aide des acides glydiciques substitués. Compt. Rend. 1904, 139, 1214–1217.

[ref10] aReutrakul V.; Jarussophon S.; Pohmakotr M.; Chaiyasut Y.; U-Thet S.; Tuchinda P. Samarium(II) iodide-mediated deoxygenative debromination of α-bromo-β-hydroxy (acetoxy) phenyl sulfones: Synthesis of α,β-unsaturated sulfones. Tetrahedron Lett. 2002, 43, 2285–2288. 10.1016/S0040-4039(02)00224-1.

[ref11] CCDC 2087404 contains the supplementary crystallographic data for this paper, which can be accessed available free of charge from The Cambridge Crystallographic Data Centre via www.ccdc.cam.ac.uk/structures

[ref12] Morgan K. F.; Doran R.; Croft R. A.; Hollingsworth I. A.; Bull J. A. 2-Sulfinyl Oxetanes: Synthesis, Stability and Reactivity. Synlett 2015, 27, 106–110. 10.1055/s-0035-1560588.

[ref13] Hayashi Y. Pot economy and one-pot synthesis. Chem. Sci. 2016, 7, 866–880. 10.1039/C5SC02913A. 28791118 PMC5529999

[ref14] Frisch M. J.; Trucks G. W.; Schlegel H. B.; Scuseria G. E.; Robb M. A.; Cheeseman J. R.; Scalmani G.; Barone V.; Petersson G. A.; Nakatsuji H.; Li X.; Caricato M.; Marenich A. V.; Bloino J.; Janesko B. G.; Gomperts R.; Mennucci B.; Hratchian H. P.; Ortiz J. V.; Izmaylov A. F.; Sonnenberg J. L.; Williams-Young D.; Ding F.; Lipparini F.; Egidi F.; Goings J.; Peng B.; Petrone A.; Henderson T.; Ranasinghe D.; Zakrzewski V. G.; Gao J.; Rega N.; Zheng G.; Liang W.; Hada M.; Ehara M.; Toyota K.; Fukuda R.; Hasegawa J.; Ishida M.; Nakajima T.; Honda Y.; Kitao O.; Nakai H.; Vreven T.; Throssell K.; Montgomery J. A. Jr.; Peralta J. E.; Ogliaro F.; Bearpark M. J.; Heyd J. J.; Brothers E. N.; Kudin K. N.; Staroverov V. N.; Keith T. A.; Kobayashi R.; Normand J.; Raghavachari K.; Rendell A. P.; Burant J. C.; Iyengar S. S.; Tomasi J.; Cossi M.; Millam J. M.; Klene M.; Adamo C.; Cammi R.; Ochterski J. W.; Martin R. L.; Morokuma K.; Farkas O.; Foresman J. B.; Fox D. J. Gaussian16, rev. C.01; Gaussian, Inc.: Wallingford, CT, 2016.

[ref15] The conformation of **4a** can be identified by the following three dihedral angles: θ_1_ ≡ C_ar_–SO_2_–CHCl–(C = O), θ_2_ ≡ Cl–C–C–O, and θ_3_ ≡ O–C–C_ar_–C_ar_(C≡N), which can be described by the IUPAC one-letter notation^16^ C, G^±^, A^±^, T (which is shorter than the corresponding Klyne–Prelog notation^17^*sp, sc*^±^, *ac*^±^, *ap*). Dihedral angle θ_2_ must be antiperiplanar (T) for epoxide formation, while θ_3_ must by synperiplanar (C) for the closure of the five-membered ring. Starting from a set of conformers obtained by Confab,^18^ we have generated new conformers by changing the values of angles θ_1_ and θ_2_. The exclusion of duplicates and high energy candidates led to 9 conformers for (*R*,*R*)-**4a** and 11 conformers for (*R*,*S*)-**4a**. Geometries were optimized in the gas phase, and energies of species in solution were obtained by a single-point PCM calculation on the gas-phase-optimized energies. Minimum energy conformers for both configurations were then reoptimized by PCM..

[ref16] International Union of Pure and Applied Chemistry; International Union of Pure and Applied Chemistry. Compendium of Polymer Terminology and Nomenclature: IUPAC Recommendations, 2008; Jones R. G., Ed.; Royal Society of Chemistry Publishing: Cambridge, U.K., 2009.

[ref17] Klyne W.; Prelog V. Description of Steric Relationships across Single Bonds. Experientia 1960, 16 (12), 521–523. 10.1007/BF02158433.

[ref18] O’Boyle N. M.; Vandermeersch T.; Flynn C. J.; Maguire A. R.; Hutchison G. R. Confab - Systematic Generation of Diverse Low-Energy Conformers. J. Cheminf. 2011, 3 (1), 8 10.1186/1758-2946-3-8. PMC3073927 21410983

[ref19] Fulmer G. R.; Miller A. J. M.; Sherden N. H.; Gottlieb H. E.; Nudelman A.; Stoltz B. M.; Bercaw J. E.; Goldberg K. I. NMR Chemical Shifts of Trace Impurities: Common Laboratory Solvents, Organics, and Gases in Deuterated Solvents Relevant to the Organometallic Chemist. Organometallics 2010, 29, 2176–2179. 10.1021/om100106e.

