# Supplementary material for: Base-Promoted Cascade Reactions for the Synthesis of 3,3-Dialkylated Isoindolin-1-ones and 3-Methyleneisoindolin-1-ones
Source: J Org Chem. 2021 Oct 6;86(21):15128–38. doi: 10.1021/acs.joc.1c01794 (PMC8576826; doi:10.1021/acs.joc.1c01794)
Supplement: Supplementary file 2 — jo1c01794_si_002.pdf [file jo1c01794_si_002.pdf]

SUPPORTING INFORMATION

Base-Promoted Cascade Reactions for the Synthesis of 3,3-Dialkylated Isoindolin-1-ones and 3-Methyleneisoindolin-1-ones

Antonio Macchia,[a] Francesco F. Summa,[a] Antonia Di Mola,[a] Consiglia Tedesco,[a] Giovanni Pierri,[a] Armin R. Ofial,[b,*] Guglielmo Monaco,[a,*] Antonio Massa[a,*]

[a] Dipartimento di Chimica e Biologia "A. Zambelli", Università degli studi di Salerno, Via Giovanni Paolo II, 84084-Fisciano (SA), Italy.
[b] Department Chemie, Ludwig-Maximilians-Universität München, 81377 München, Germany

Table of Contents

1. Copies of ^{1}H and ^{13}C{^{1}H} NMR S2

1.1. Copies of ^{1}H and ^{13}C{^{1}H} NMR of 3,3-Disubstituted Isoindolinones with Substituted ((Chloromethyl) sulfonyl)benzene S2

1.2. Copies of ^{1}H and ^{13}C{^{1}H} NMR of substituted (*Z*)-3-((Phenylsulfonyl)methylene)isoindolin-1-ones S10

1.3. Copies of ^{1}H and ^{13}C{^{1}H} NMR of N-Alkylated (*Z, E)*-3 ((Phenylsulfonyl) methylene)isoindolin-1-ones S18

2. X-ray crystallography S24

3. DFT Investigation S26

4. References S51

1. Copies of ^{1}H and $^{13}C\{^{1}H\}$ NMR

1.1. Copies of ^{1}H and $^{13}C\{^{1}H\}$ NMR of 3,3-Disubstituted Isoindolinones with Substituted ((Chloromethyl) sulfonyl)benzene

^{1}H NMR (400 MHz, $CDCl_3$)

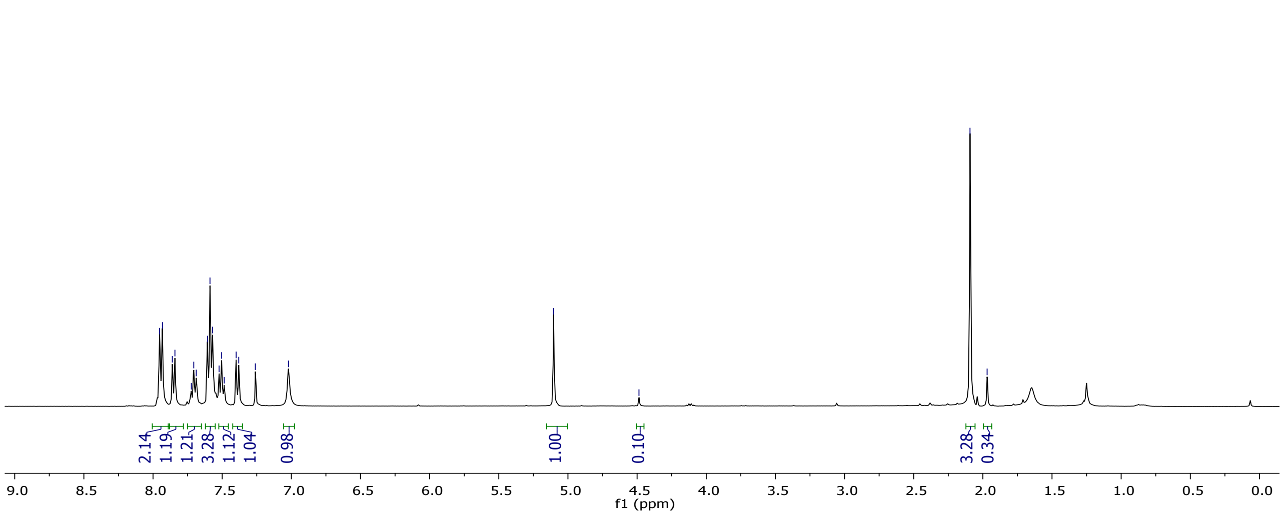

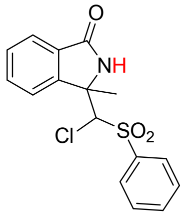

7a

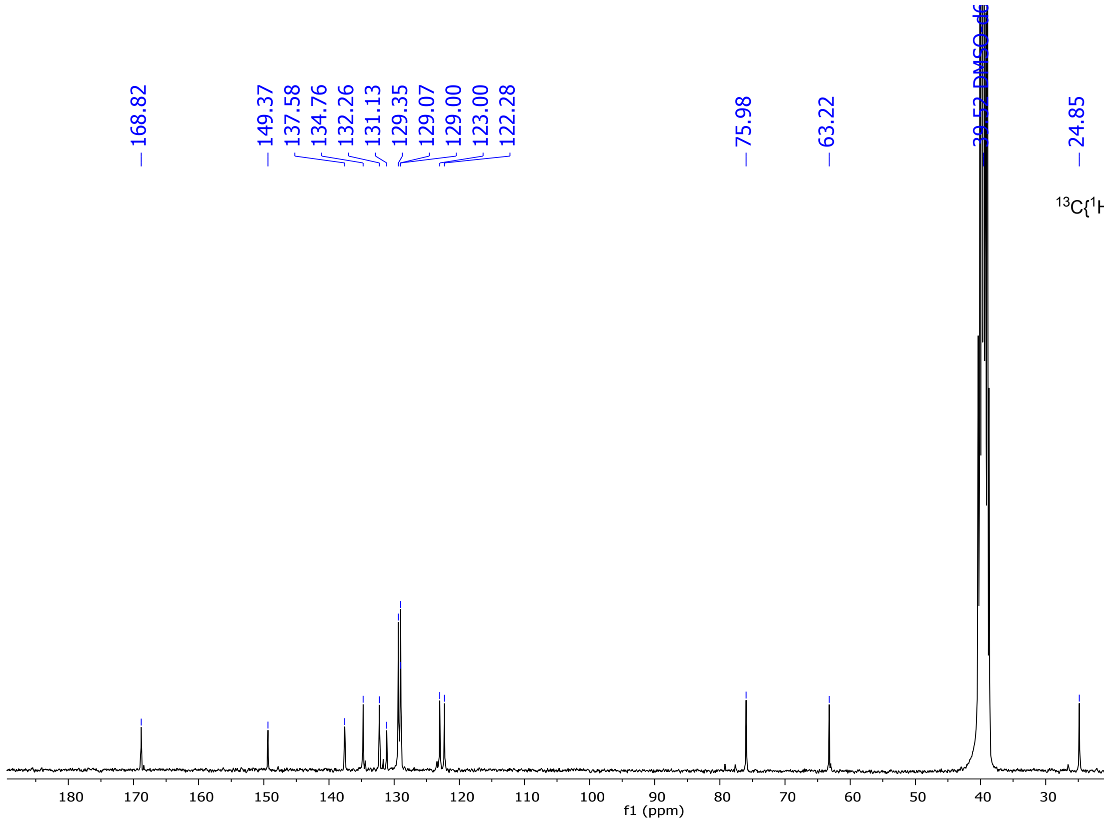

$^{13}C\{^{1}H\}$ NMR (75.5 MHz, DMSO-d_6)

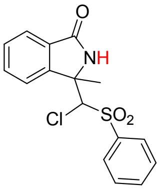

7a

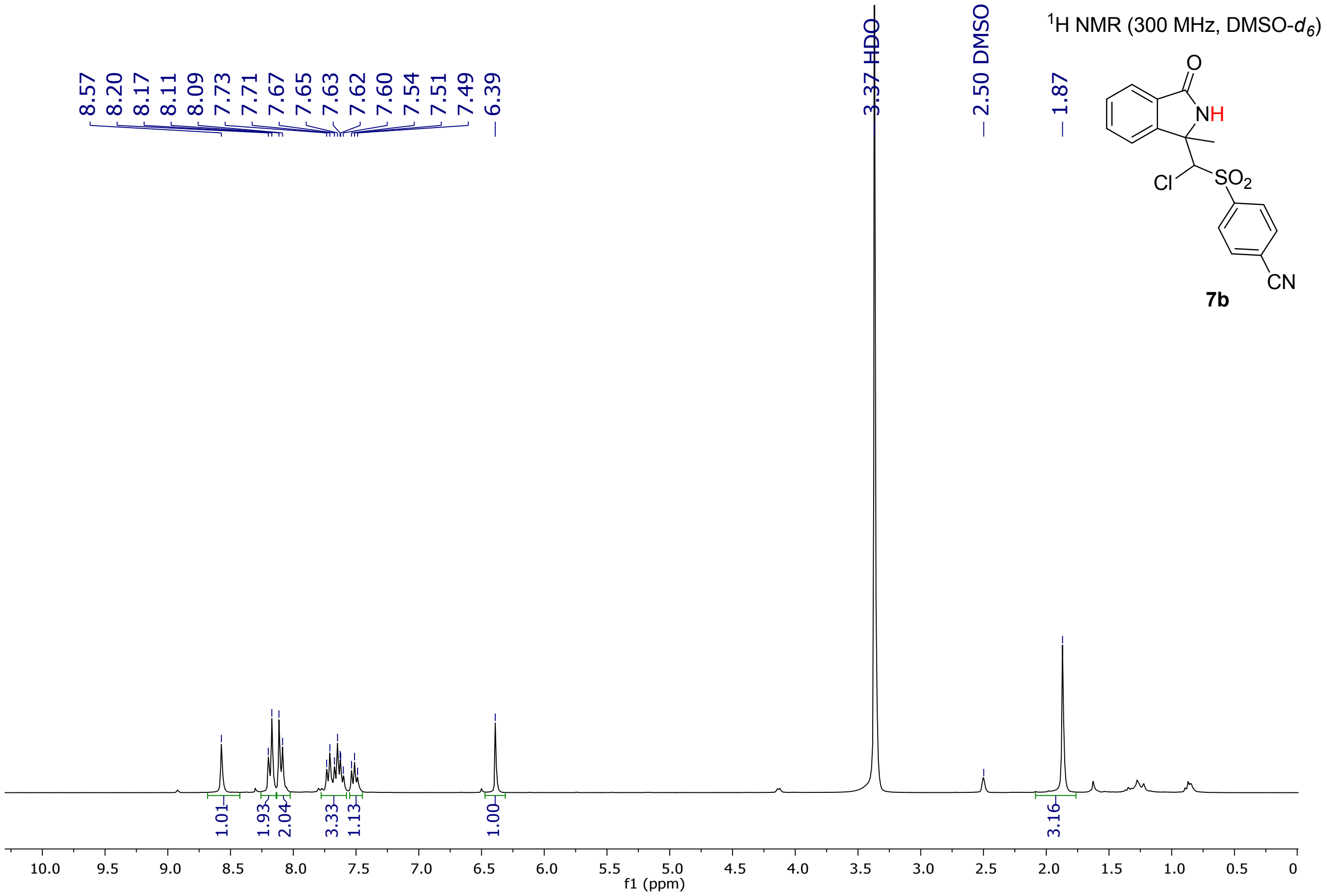
^{1}H NMR (300 MHz, DMSO-d_6)
8.57 8.20 8.17 8.11 8.09 7.73 7.71 7.67 7.65 7.63 7.62 7.60 7.54 7.51 7.49 6.39
3.37 HDO
2.50 DMSO
1.87
1.01 1.93 2.04 3.33 1.13 1.00 3.16
f1 (ppm)
O
NH
Cl
SO_2
CN
7b

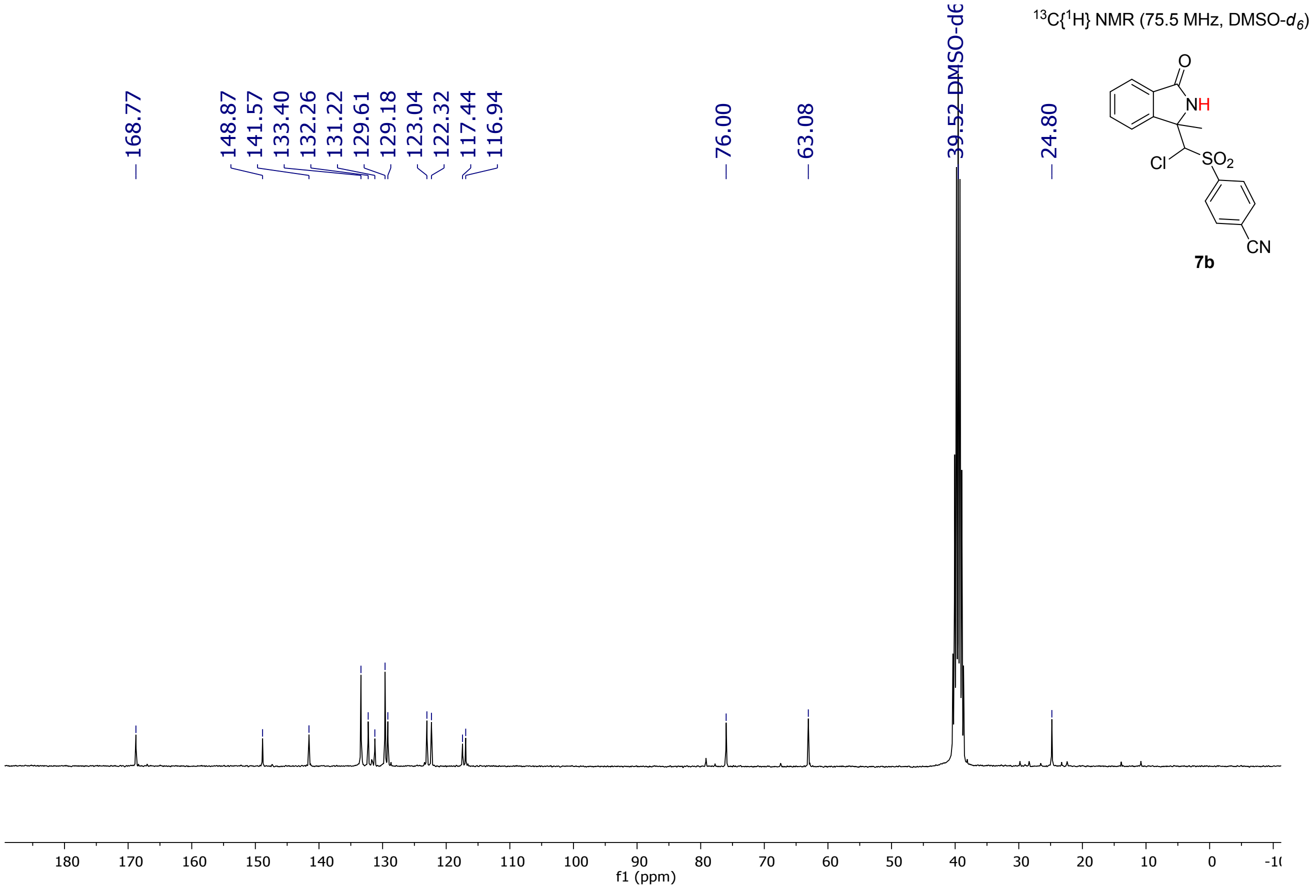
^{13}C{^{1}H} NMR (75.5 MHz, DMSO-d_6)
168.77
148.87 141.57 133.40 132.26 131.22 129.61 129.18 123.04 122.32 117.44 116.94
76.00
63.08
39.52 DMSO-d6
24.80
f1 (ppm)
O
NH
Cl
SO_2
CN
7b

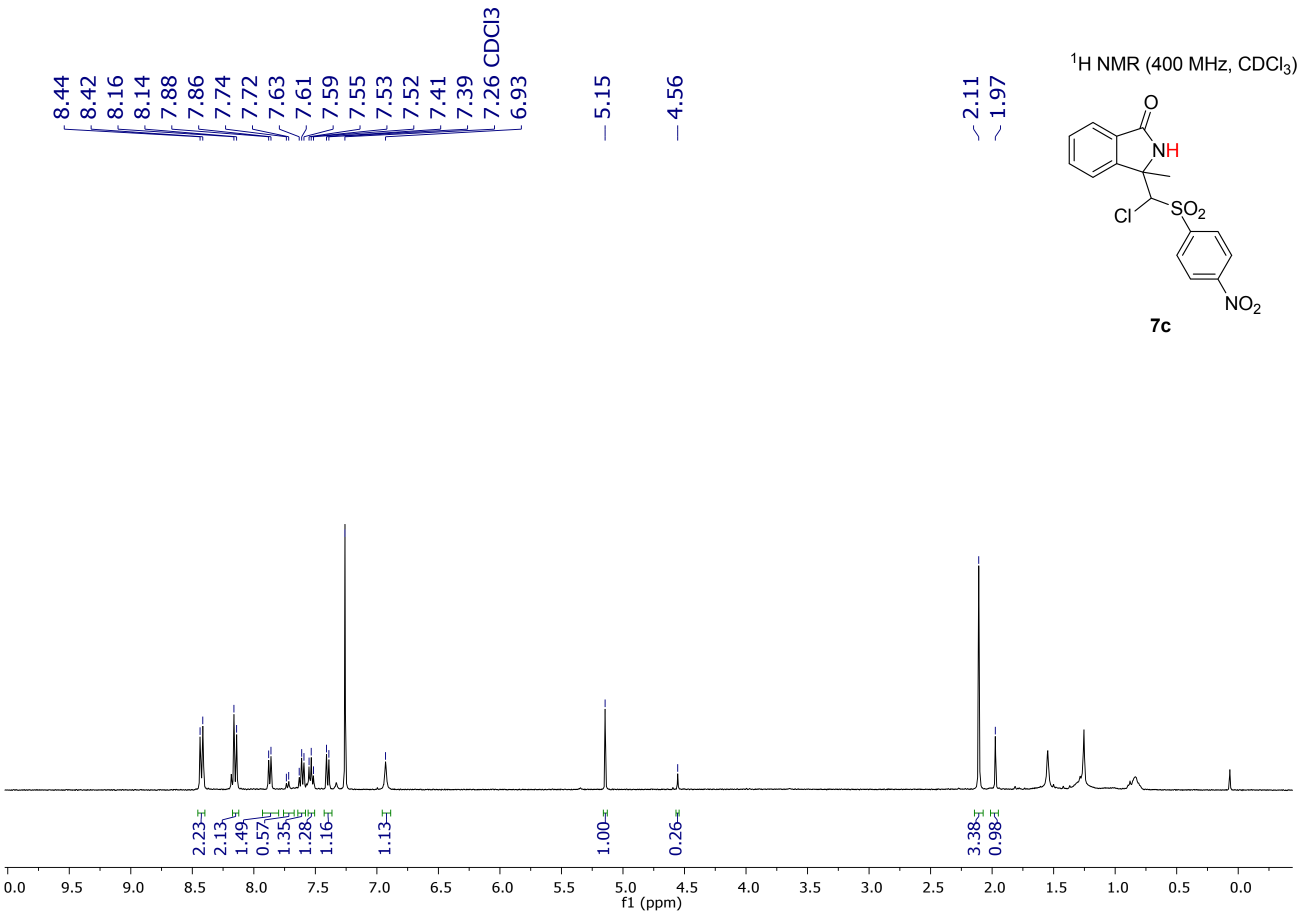

8.44 8.42 8.16 8.14 7.88 7.86 7.74 7.72 7.63 7.61 7.59 7.55 7.53 7.52 7.41 7.39 7.26 CDCl3 6.93
5.15
4.56
2.11 1.97
^{1}H NMR (400 MHz, $CDCl_3$)
O
NH
Cl
SO_2
NO_2
7c
2.23 2.13 1.49 0.57 1.35 1.28 1.16 1.13 1.00 0.26 3.38 0.98
f1 (ppm)

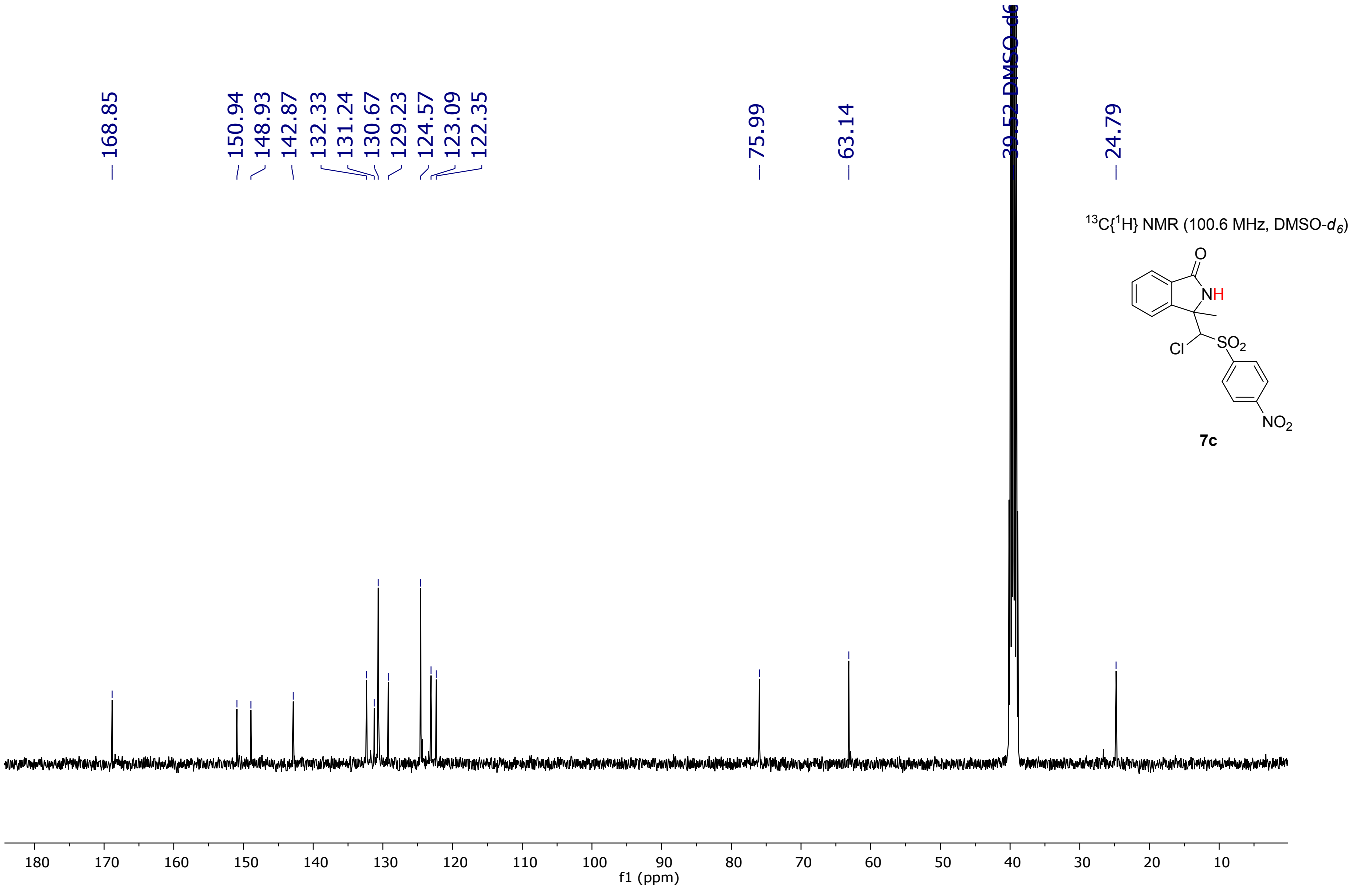

168.85
150.94 148.93 142.87 132.33 131.24 130.67 129.23 124.57 123.09 122.35
75.99
63.14
24.79
$^{13}C\{^{1}H\}$ NMR (100.6 MHz, DMSO-d_6)
O
NH
Cl
SO_2
NO_2
7c
f1 (ppm)

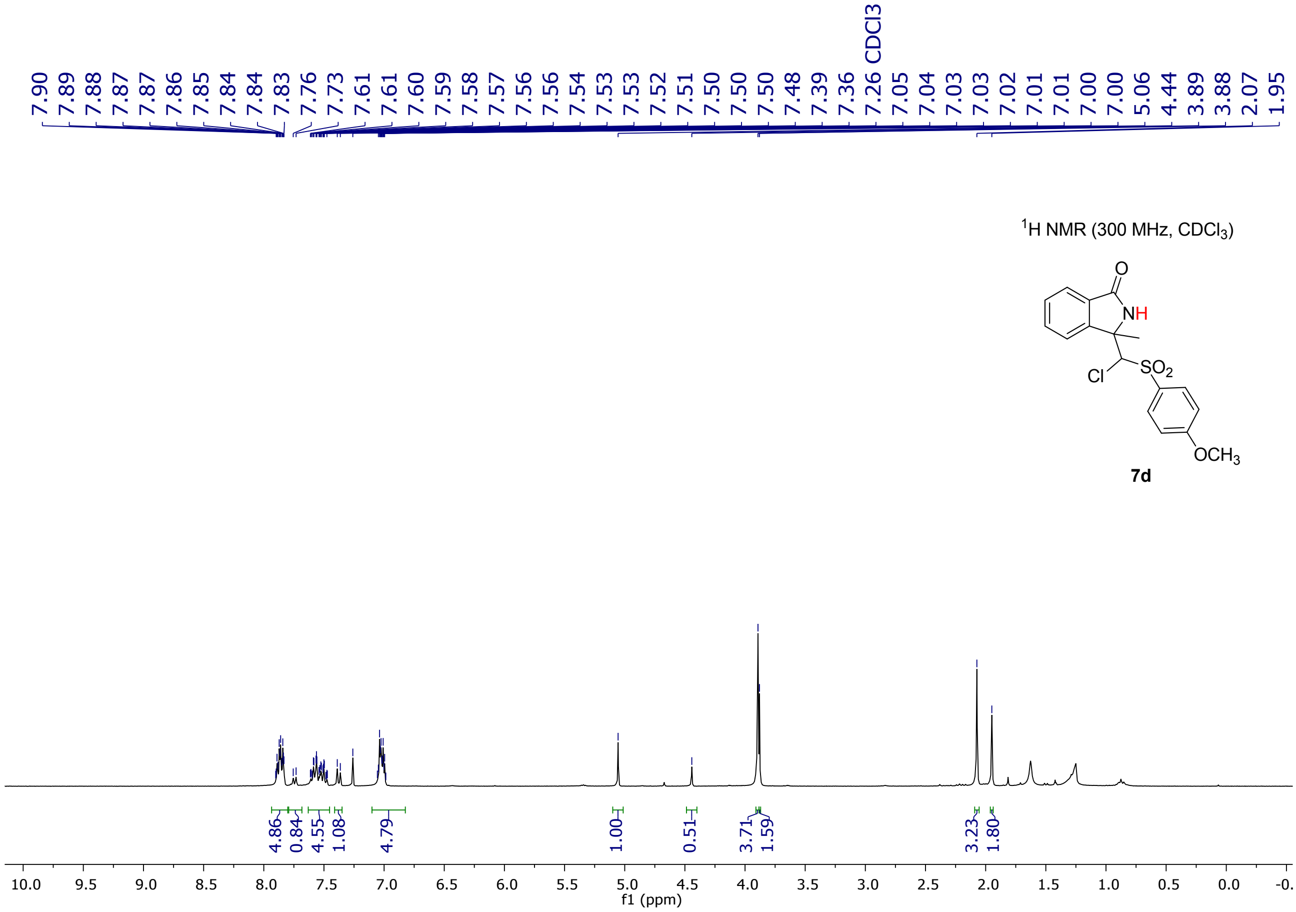
^{1}H NMR (300 MHz, CDCl$_3$)
7d

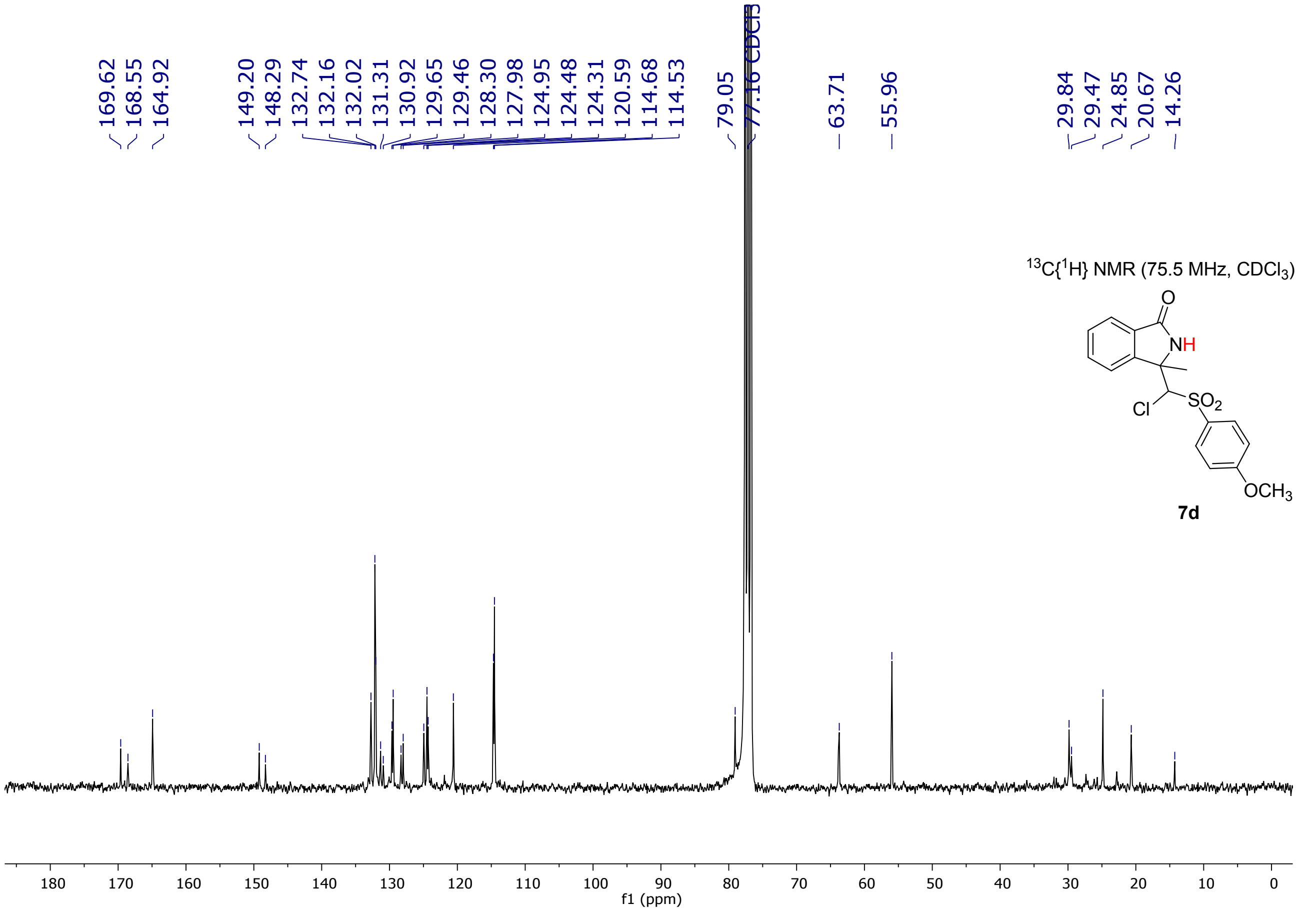
^{13}C{^{1}H} NMR (75.5 MHz, CDCl$_3$)
7d

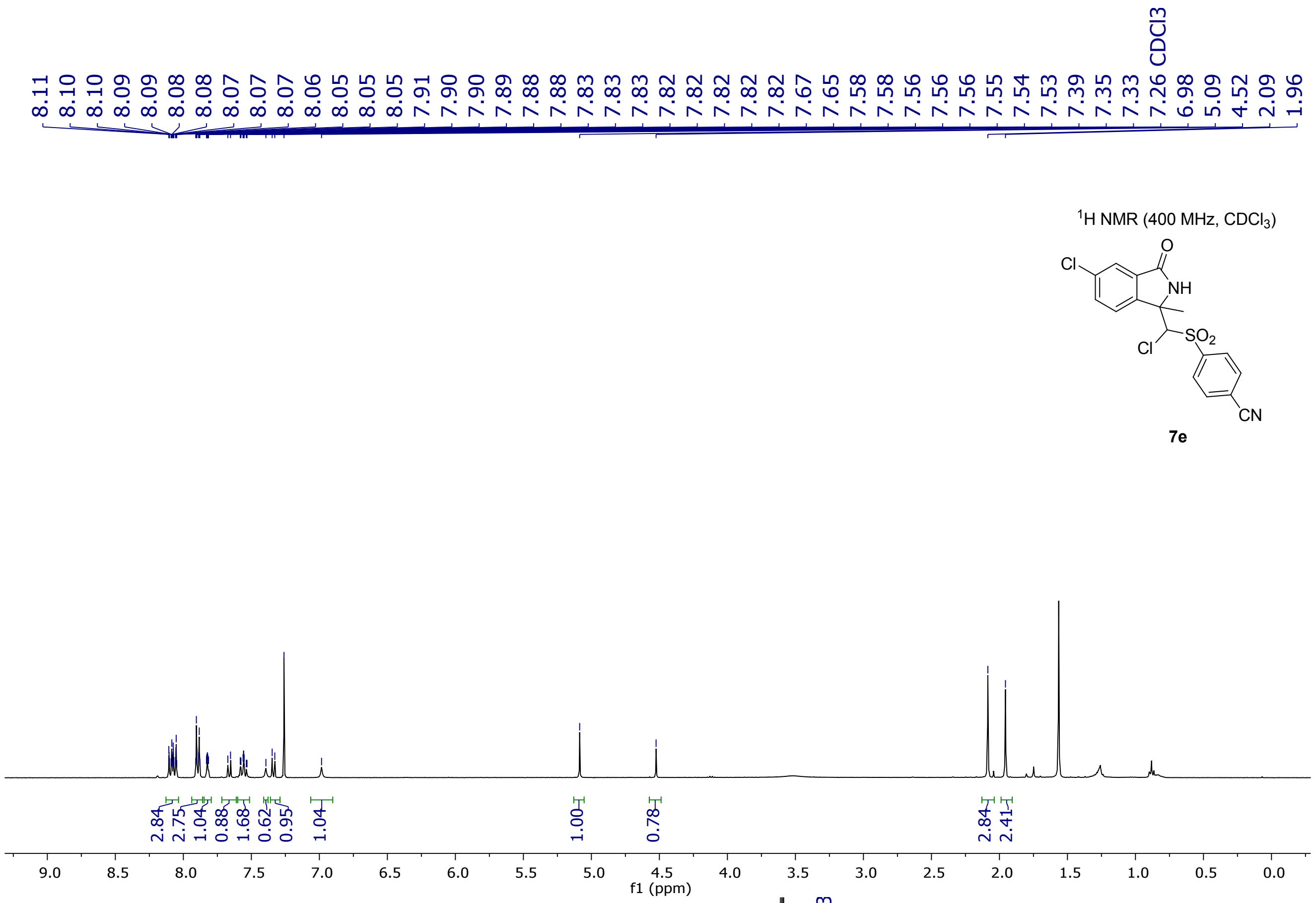

8.11 8.10 8.10 8.09 8.09 8.08 8.08 8.07 8.07 8.07 8.06 8.05 8.05 8.05 7.91 7.90 7.90 7.89 7.88 7.88 7.83 7.83 7.83 7.82 7.82 7.82 7.82 7.82 7.67 7.65 7.58 7.58 7.56 7.56 7.56 7.55 7.54 7.53 7.39 7.35 7.33 7.26 CDCl3 6.98 5.09 4.52 2.09 1.96
1H NMR (400 MHz, CDCl3)
Cl
O
NH
SO2
Cl
CN
7e
2.84 2.75 1.04 0.88 1.68 0.62 0.95 1.04 1.00 0.78 2.84 2.41
f1 (ppm)

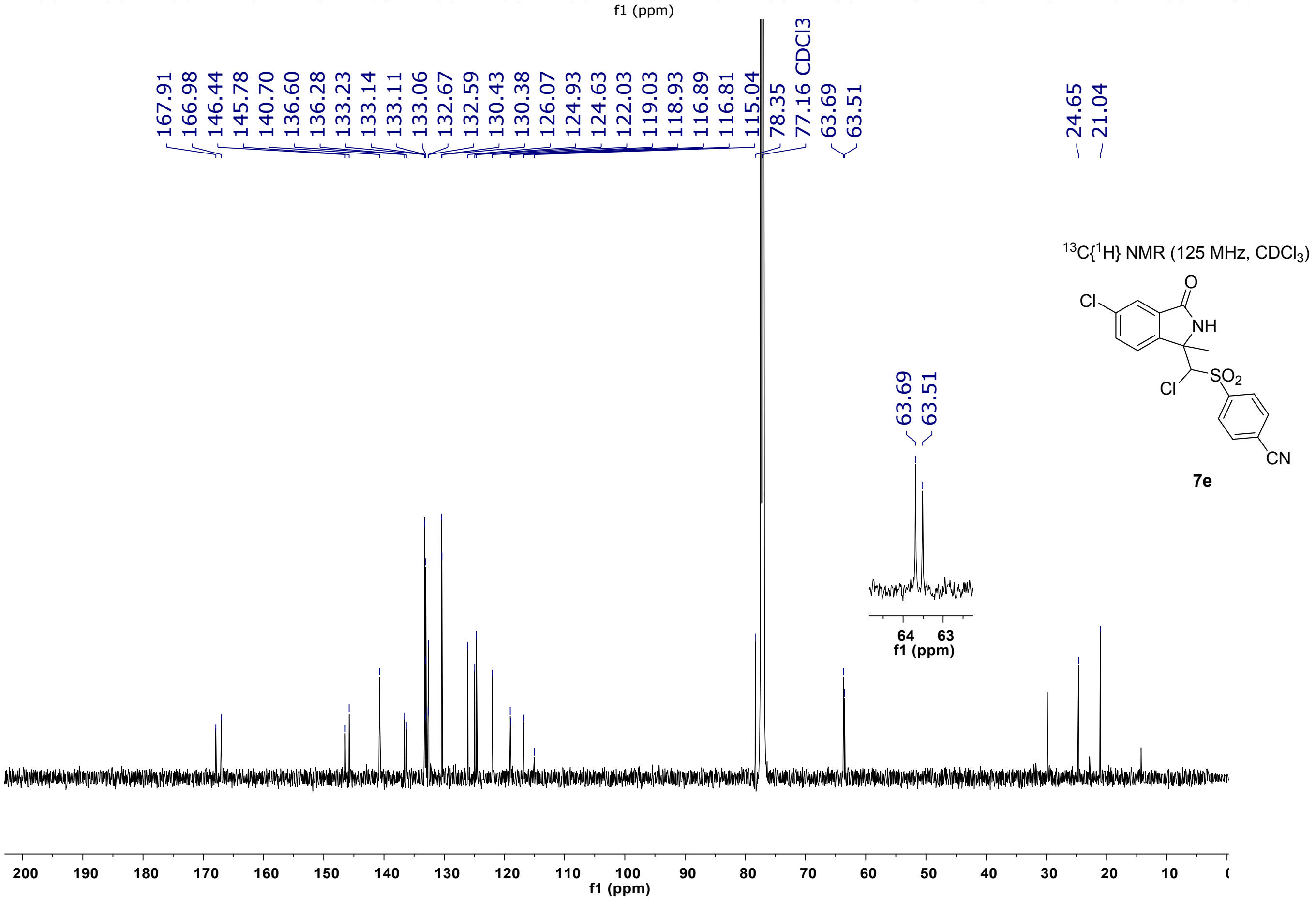

167.91 166.98 146.44 145.78 140.70 136.60 136.28 133.23 133.14 133.11 133.06 132.67 132.59 130.43 130.38 126.07 124.93 124.63 122.03 119.03 118.93 116.89 116.81 115.04 78.35 77.16 CDCl3 63.69 63.51 24.65 21.04
13C{1H} NMR (125 MHz, CDCl3)
Cl
O
NH
SO2
Cl
CN
7e
63.69 63.51
f1 (ppm)
f1 (ppm)

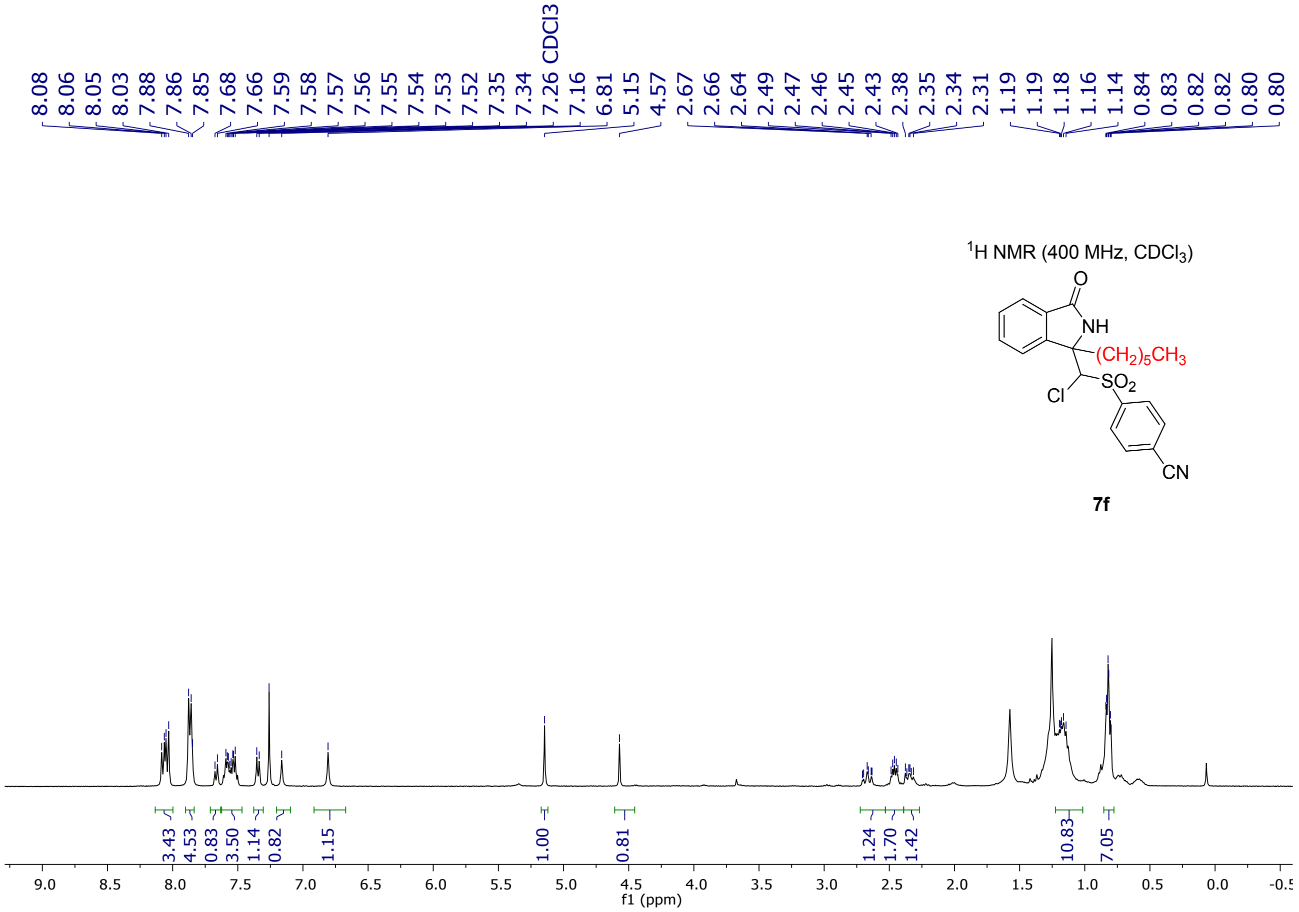
8.08 8.06 8.05 8.03 7.88 7.86 7.85 7.68 7.66 7.59 7.58 7.57 7.56 7.55 7.54 7.53 7.52 7.35 7.34 7.26 CDCl3 7.16 6.81 5.15 4.57 2.67 2.66 2.64 2.49 2.47 2.46 2.45 2.43 2.38 2.35 2.34 2.31 1.19 1.19 1.18 1.16 1.14 0.84 0.83 0.82 0.82 0.80 0.80
1H NMR (400 MHz, CDCl3)
O
NH
(CH2)5CH3
SO2
Cl
CN
7f
3.43 4.53 0.83 3.50 1.14 0.82 1.15 1.00 0.81 1.24 1.70 1.42 10.83 7.05
9.0 8.5 8.0 7.5 7.0 6.5 6.0 5.5 5.0 4.5 4.0 3.5 3.0 2.5 2.0 1.5 1.0 0.5 0.0 -0.5
f1 (ppm)

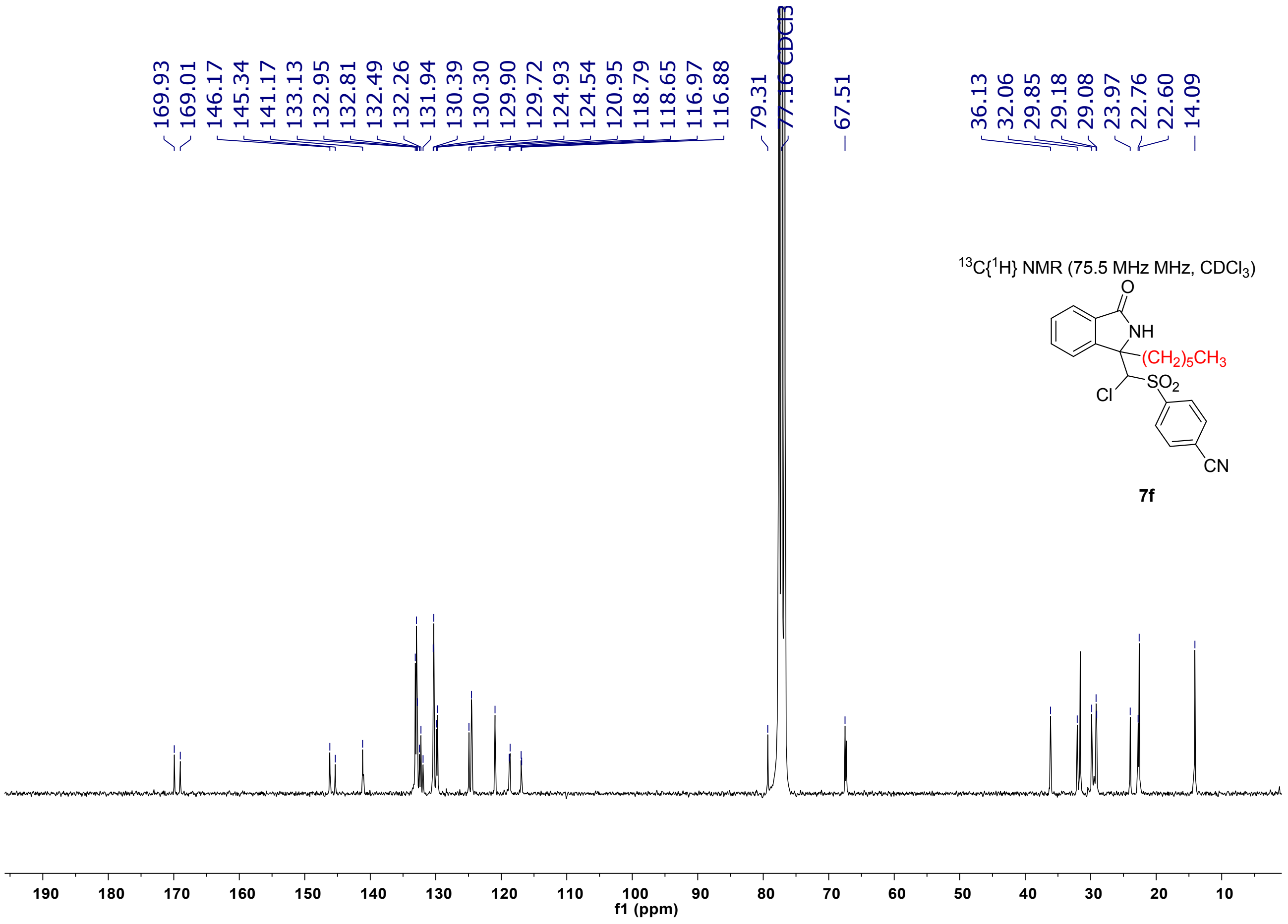
169.93 169.01 146.17 145.34 141.17 133.13 132.95 132.81 132.49 132.26 131.94 130.39 130.30 129.90 129.72 124.93 124.54 120.95 118.79 118.65 116.97 116.88 79.31 77.16 CDCl3 67.51 36.13 32.06 29.85 29.18 29.08 23.97 22.76 22.60 14.09
13C{1H} NMR (75.5 MHz MHz, CDCl3)
O
NH
(CH2)5CH3
SO2
Cl
CN
7f
190 180 170 160 150 140 130 120 110 100 90 80 70 60 50 40 30 20 10
f1 (ppm)

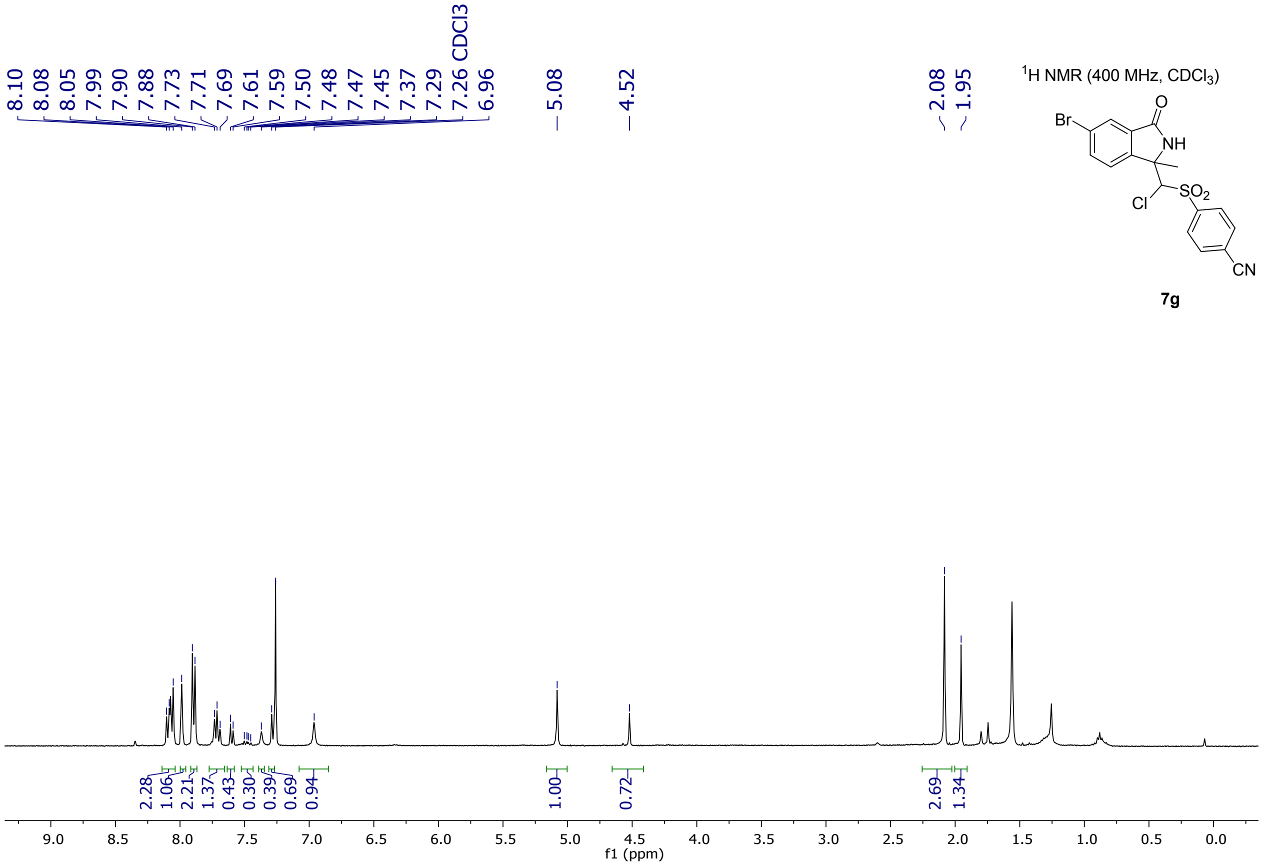
8.10 8.08 8.05 7.99 7.90 7.88 7.73 7.71 7.69 7.61 7.59 7.50 7.48 7.47 7.45 7.37 7.29 7.26 CDCl3 6.96
5.08
4.52
2.08 1.95
^{1}H NMR (400 MHz, $CDCl_3$)
Br
O
NH
SO$_2$
Cl
CN
7g
2.28 1.06 2.21 1.37 0.43 0.30 0.39 0.69 0.94 1.00 0.72 2.69 1.34
f1 (ppm)

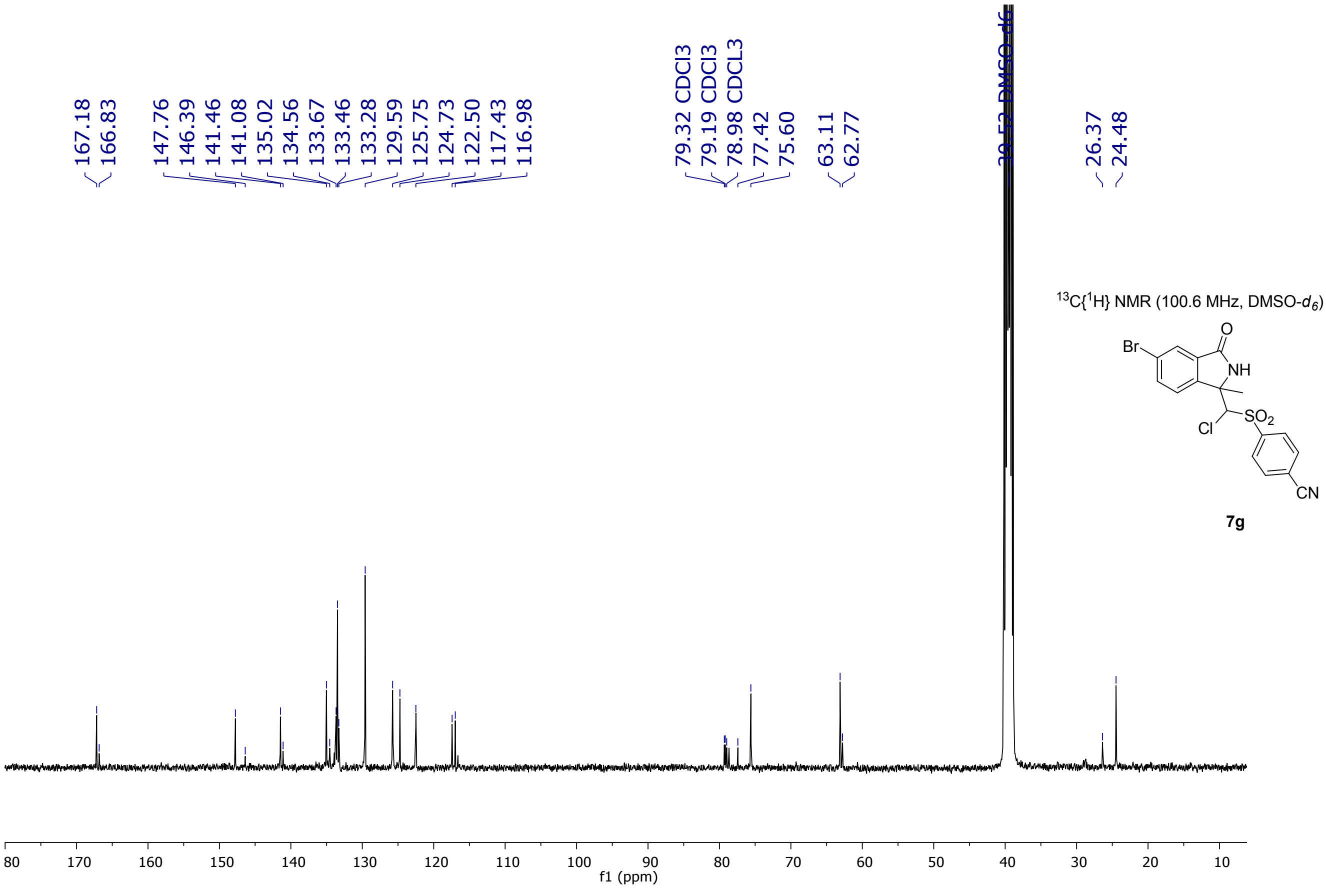
167.18 166.83
147.76 146.39 141.46 141.08 135.02 134.56 133.67 133.46 133.28 129.59 125.75 124.73 122.50 117.43 116.98
79.32 CDCl3 79.19 CDCl3 78.98 CDCL3 77.42 75.60
63.11 62.77
26.37 24.48
^{13}C{^{1}H} NMR (100.6 MHz, DMSO-d_6)
Br
O
NH
SO$_2$
Cl
CN
7g
f1 (ppm)

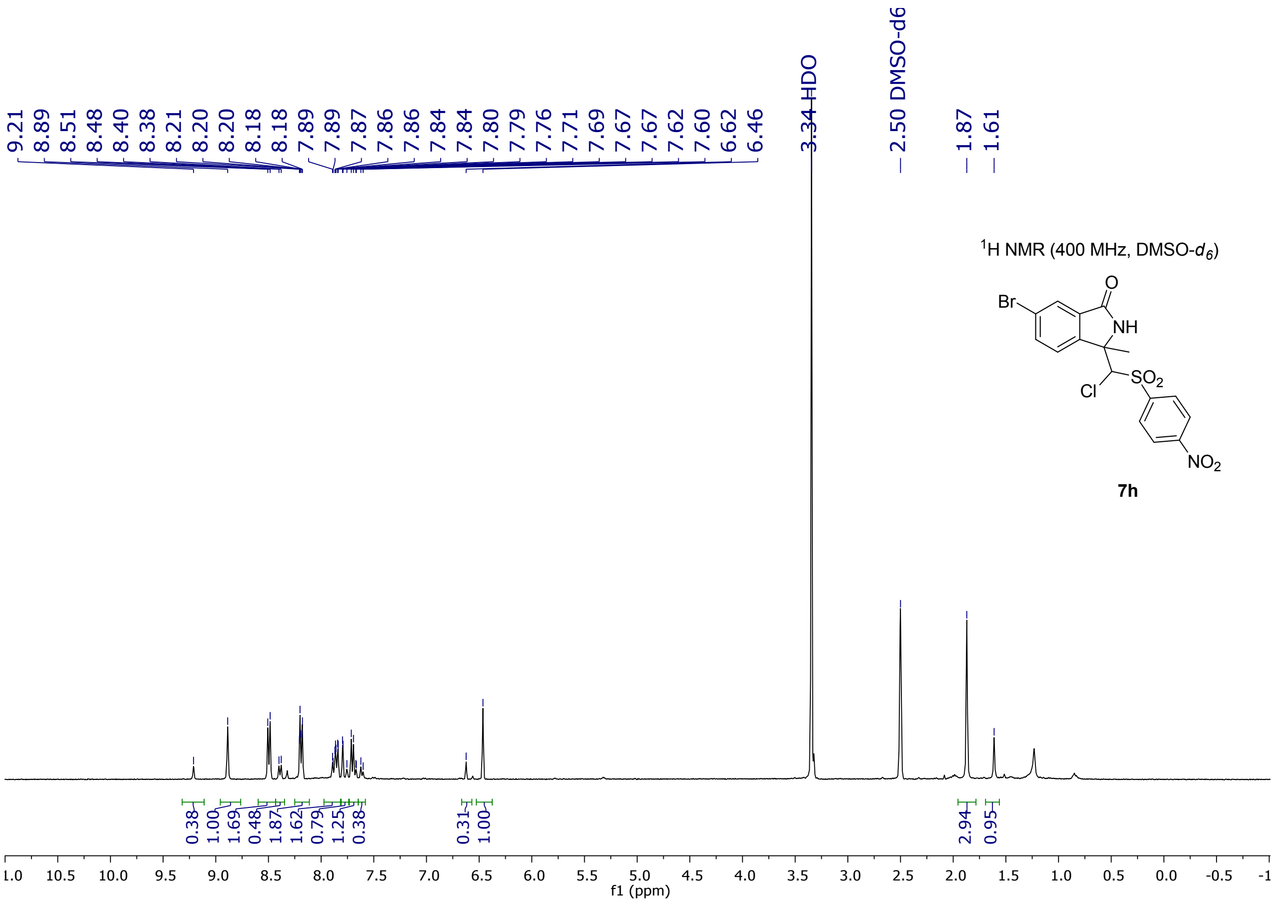
9.21 8.89 8.51 8.48 8.40 8.38 8.21 8.20 8.20 8.18 8.18 7.89 7.89 7.87 7.86 7.86 7.84 7.84 7.80 7.79 7.76 7.71 7.69 7.67 7.67 7.62 7.60 6.62 6.46
3.34 HDO
2.50 DMSO-d6
1.87
1.61
^{1}H NMR (400 MHz, DMSO-d_6)
Br
O
NH
SO$_2$
Cl
NO$_2$
7h
0.38 1.00 1.69 0.48 1.87 1.62 0.79 1.25 0.38 0.31 1.00 2.94 0.95
f1 (ppm)

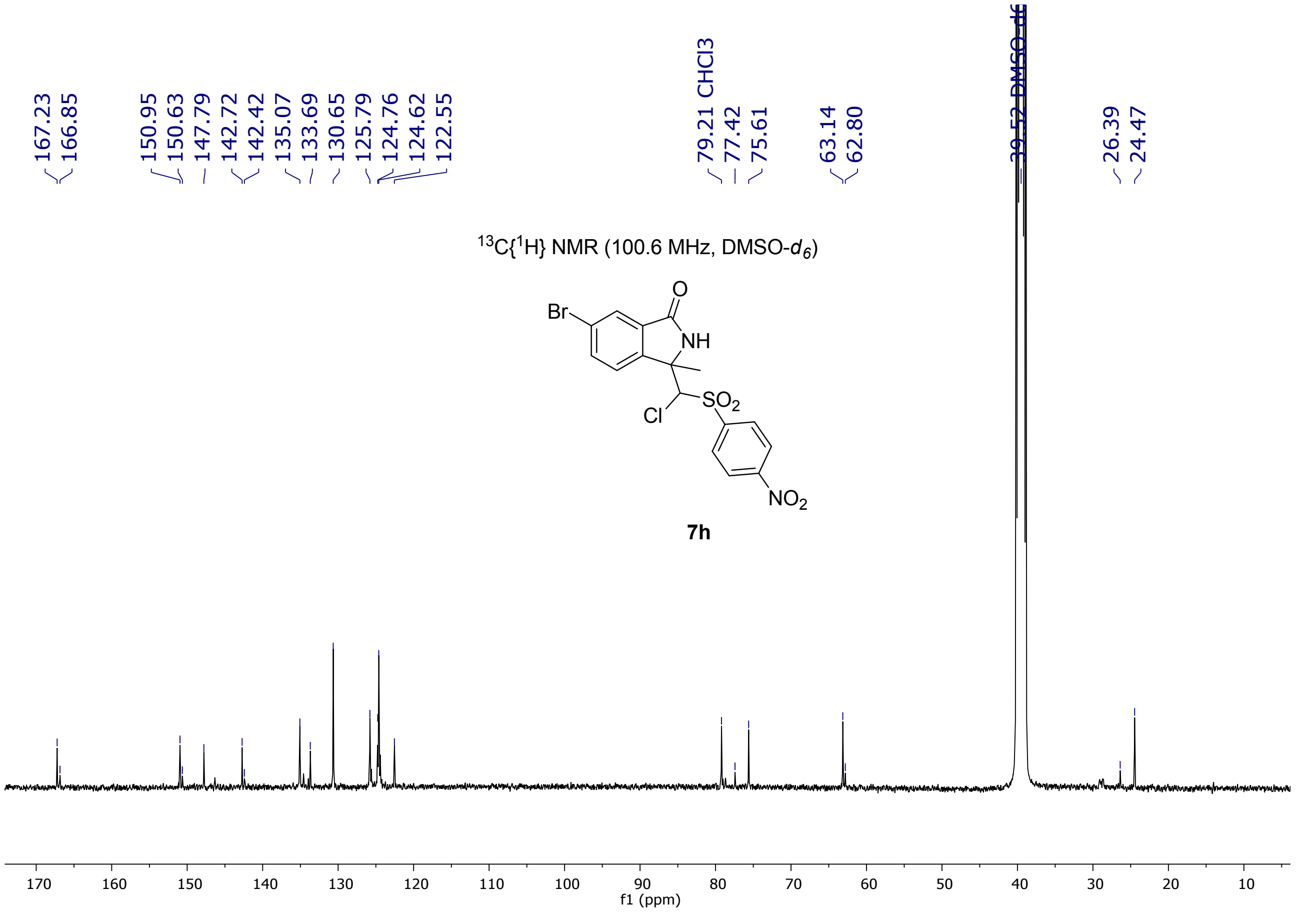
167.23 166.85 150.95 150.63 147.79 142.72 142.42 135.07 133.69 130.65 125.79 124.76 124.62 122.55
79.21 CHCl3
77.42
75.61
63.14
62.80
39.52 DMSO-d6
26.39
24.47
^{13}C{^{1}H} NMR (100.6 MHz, DMSO-d_6)
Br
O
NH
SO$_2$
Cl
NO$_2$
7h
f1 (ppm)

1.2. Copies of ^{1}H and ^{13}C{^{1}H} NMR of substituted (*Z*)-3-((Phenylsulfonyl)methylene)isoindolin-1-ones

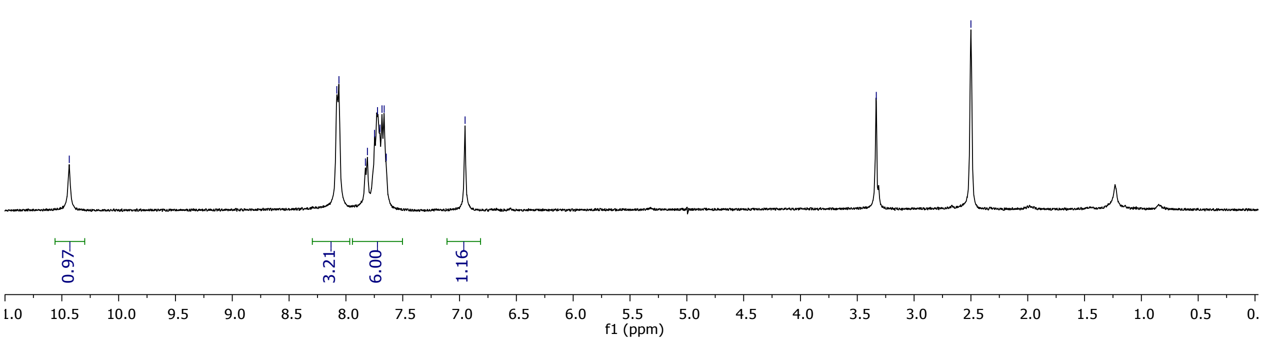

^{1}H NMR (400 MHz, DMSO-d_6)

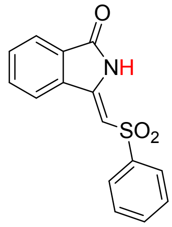

8a

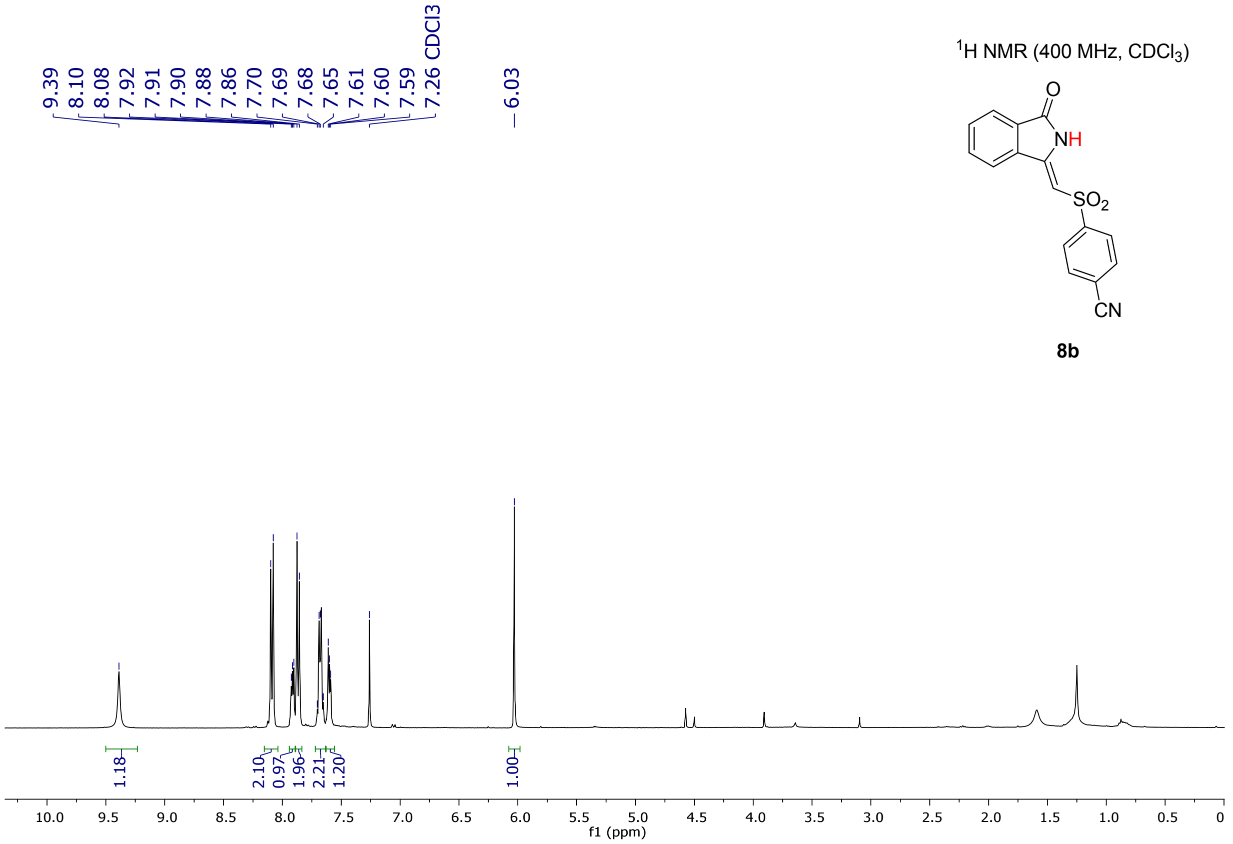

9.39
8.10
8.08
7.92
7.91
7.90
7.88
7.86
7.70
7.69
7.68
7.65
7.61
7.60
7.59
7.26 CDCl3
6.03
^{1}H NMR (400 MHz, $CDCl_3$)
O
NH
SO_2
CN
8b
1.18
2.10
0.97
1.96
2.21
1.20
1.00
10.0
9.5
9.0
8.5
8.0
7.5
7.0
6.5
6.0
5.5
5.0
4.5
4.0
3.5
3.0
2.5
2.0
1.5
1.0
0.5
0
f1 (ppm)

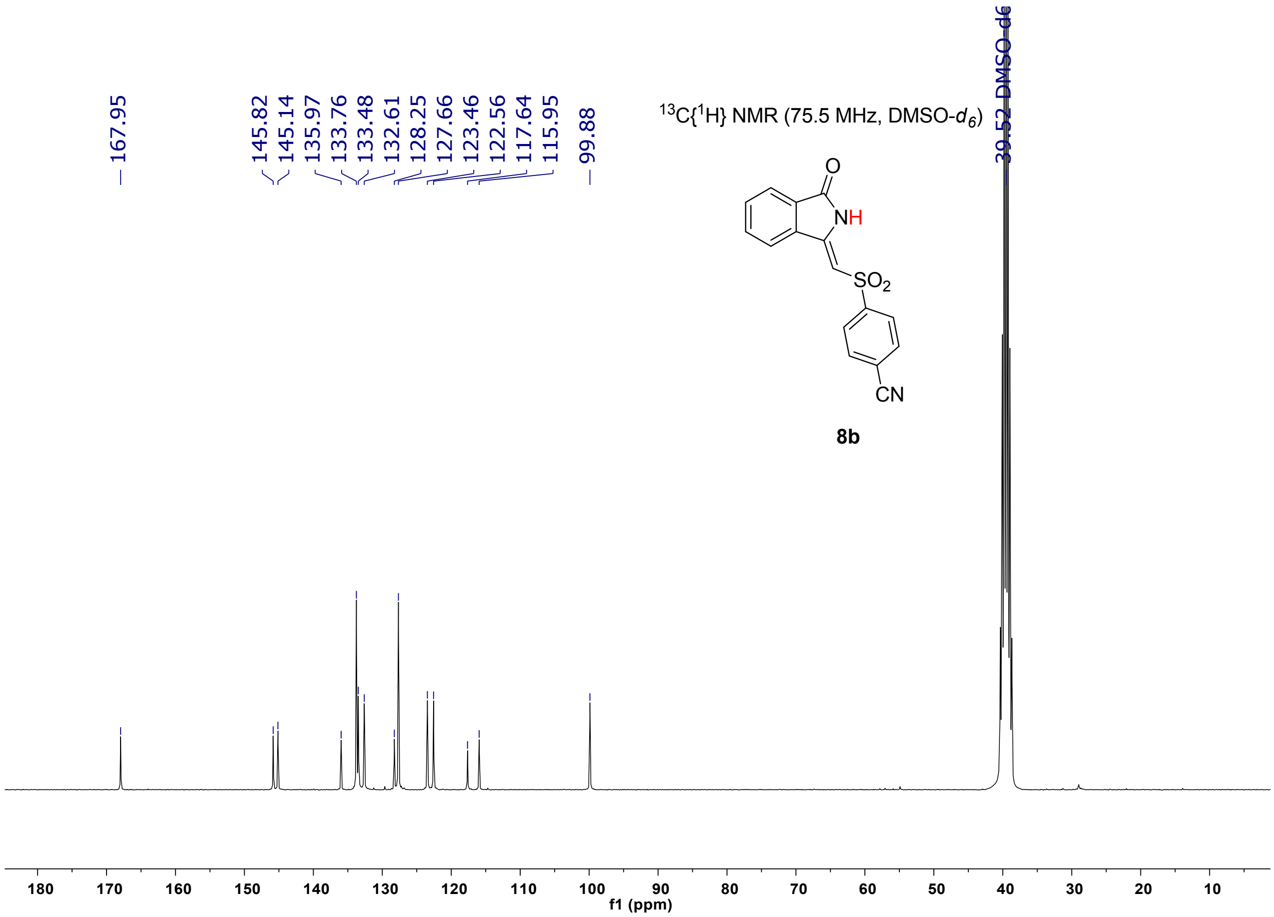

167.95
145.82
145.14
135.97
133.76
133.48
132.61
128.25
127.66
123.46
122.56
117.64
115.95
99.88
39.52 DMSO-d6
$^{13}C\{^{1}H\}$ NMR (75.5 MHz, DMSO-d_6)
O
NH
SO_2
CN
8b
180
170
160
150
140
130
120
110
100
90
80
70
60
50
40
30
20
10
f1 (ppm)

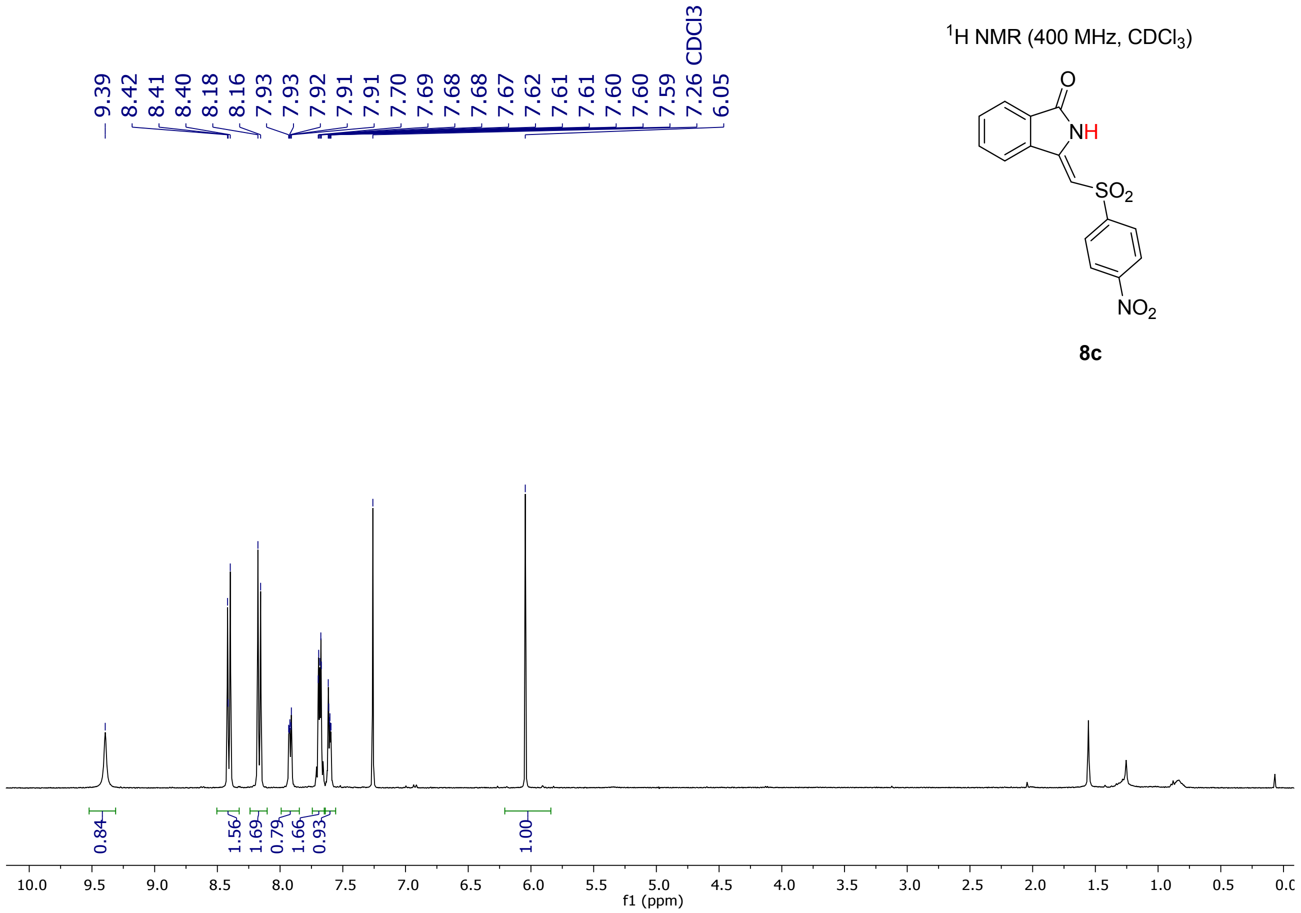

^{1}H NMR (400 MHz, $CDCl_3$)
NH
SO2
NO2
8c
9.39 8.42 8.41 8.40 8.18 8.16 7.93 7.93 7.92 7.91 7.91 7.70 7.69 7.68 7.68 7.67 7.62 7.61 7.61 7.60 7.60 7.59 7.26 CDCl3 6.05
0.84 1.56 1.69 0.79 1.66 0.93 1.00
f1 (ppm)

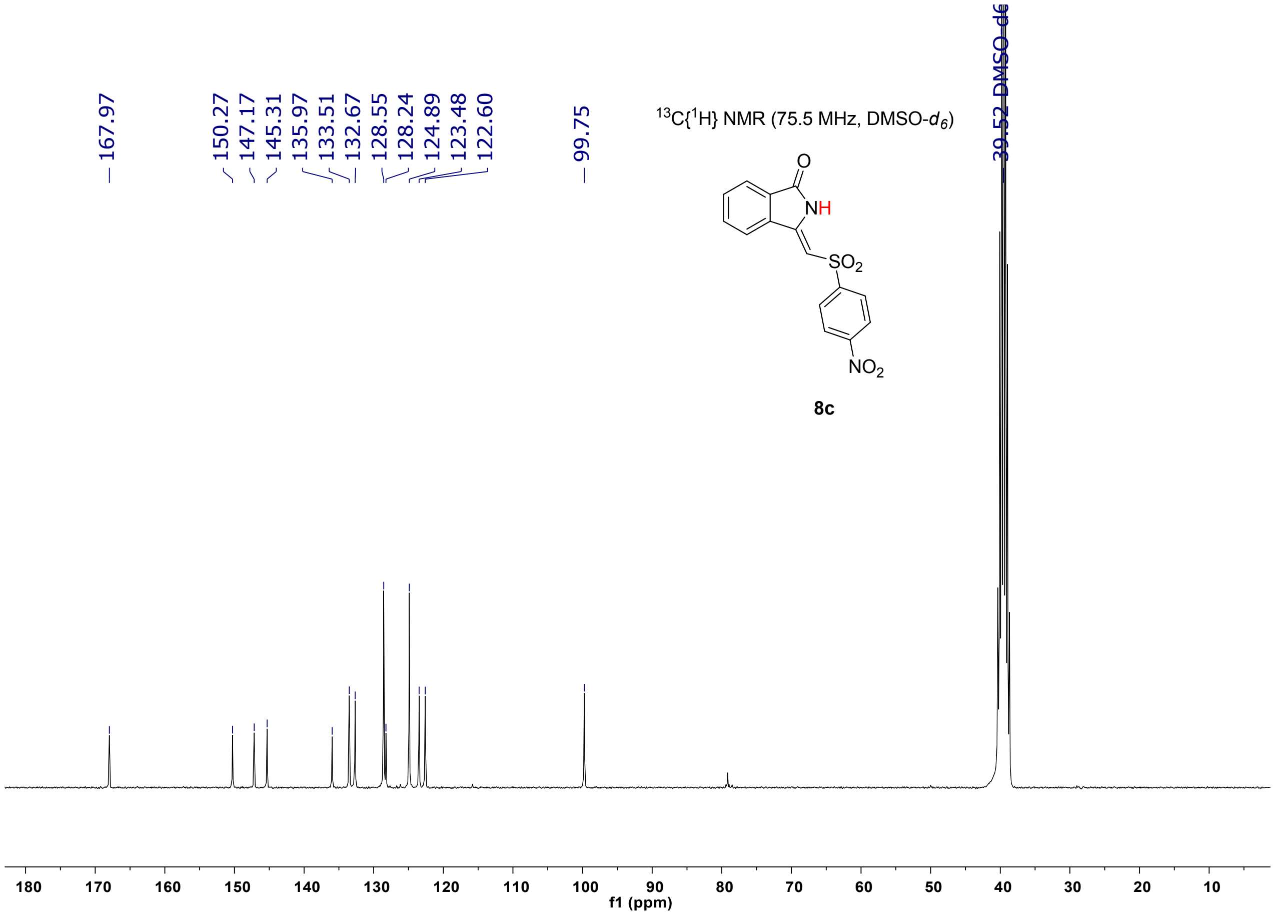

^{13}C{^{1}H} NMR (75.5 MHz, DMSO-d_6)
NH
SO2
NO2
8c
167.97 150.27 147.17 145.31 135.97 133.51 132.67 128.55 128.24 124.89 123.48 122.60 99.75 39.52 DMSO-d6
f1 (ppm)

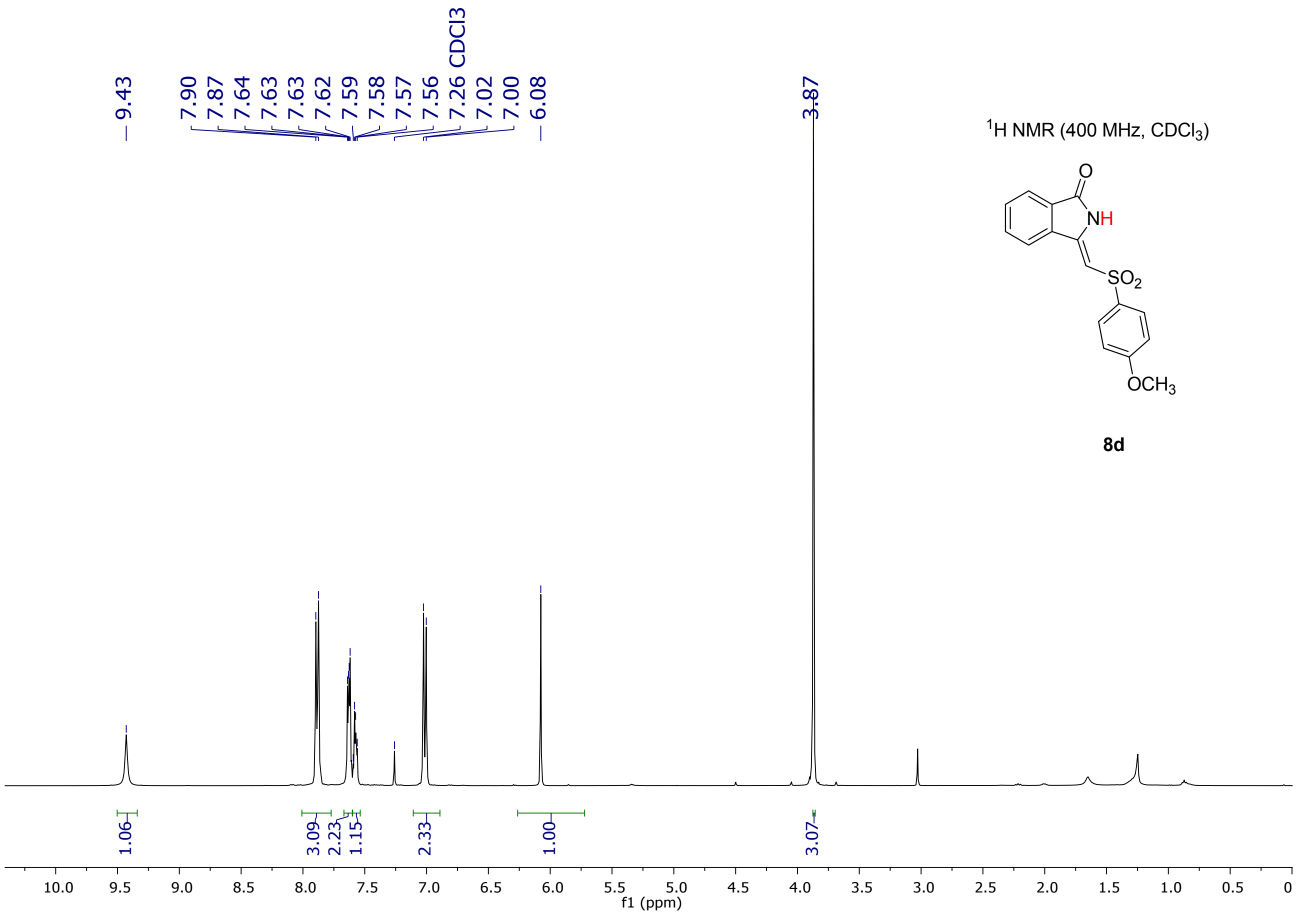

9.43
7.90
7.87
7.64
7.63
7.63
7.62
7.59
7.58
7.57
7.56
7.26 CDCl3
7.02
7.00
6.08
3.87
1H NMR (400 MHz, CDCl3)
O
NH
SO2
OCH3
8d
1.06
3.09
2.23
1.15
2.33
1.00
3.07
f1 (ppm)

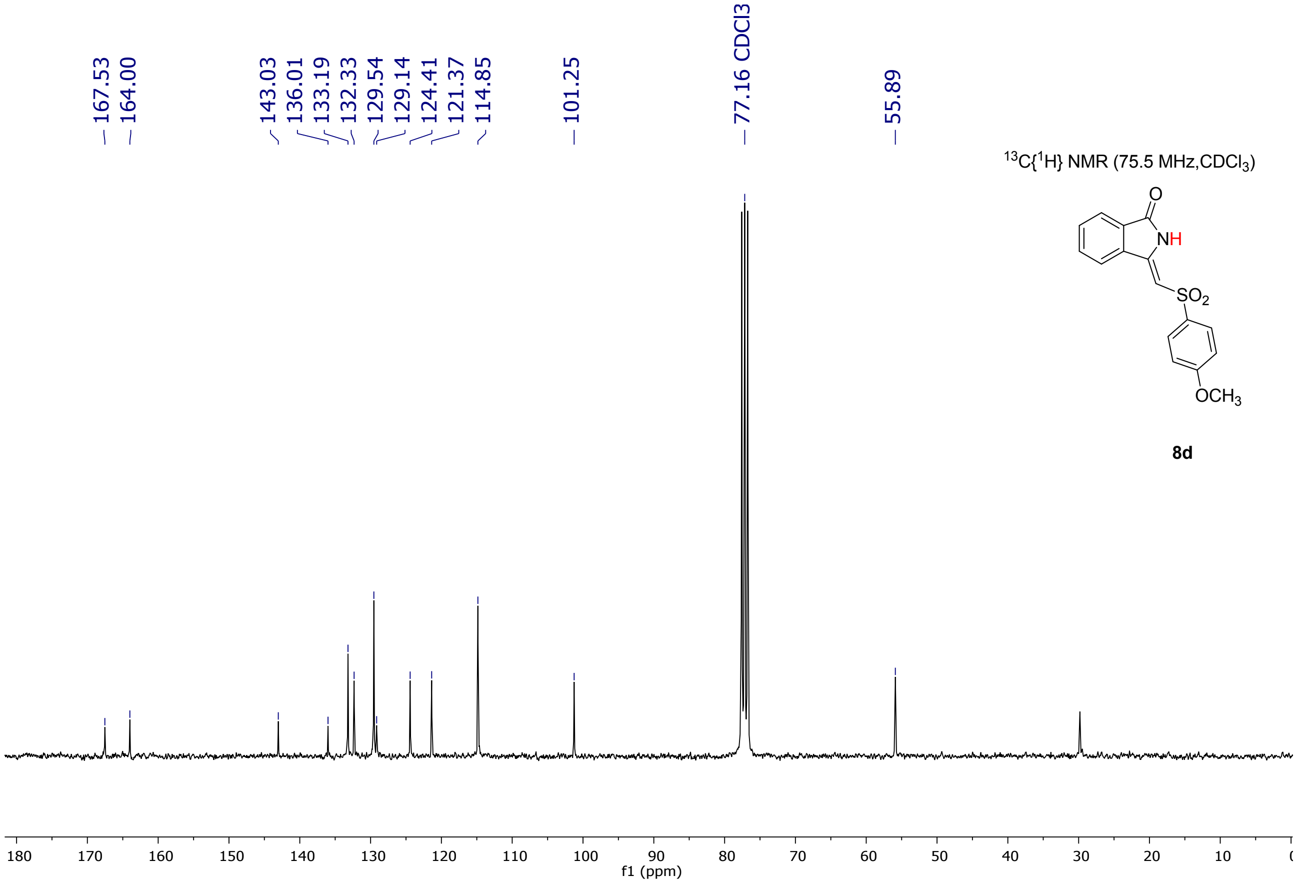

167.53
164.00
143.03
136.01
133.19
132.33
129.54
129.14
124.41
121.37
114.85
101.25
77.16 CDCl3
55.89
13C{1H} NMR (75.5 MHz,CDCl3)
O
NH
SO2
OCH3
8d
f1 (ppm)

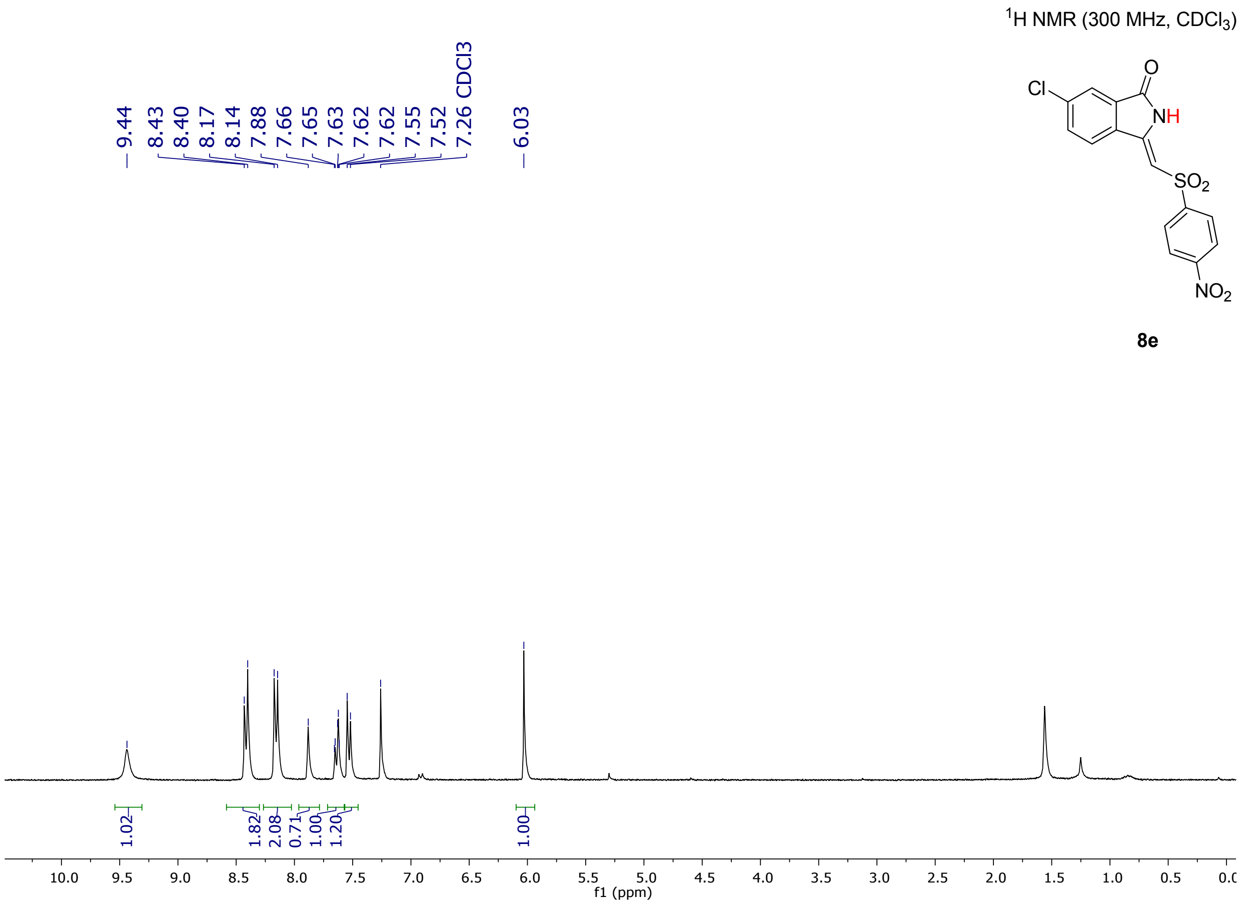

^{1}H NMR (300 MHz, $CDCl_3$)
9.44
8.43
8.40
8.17
8.14
7.88
7.66
7.65
7.63
7.62
7.62
7.55
7.52
7.26 CDCl3
6.03
Cl
O
NH
SO_2
NO_2
1.02
1.82
2.08
0.71
1.00
1.20
1.00
f1 (ppm)

8e

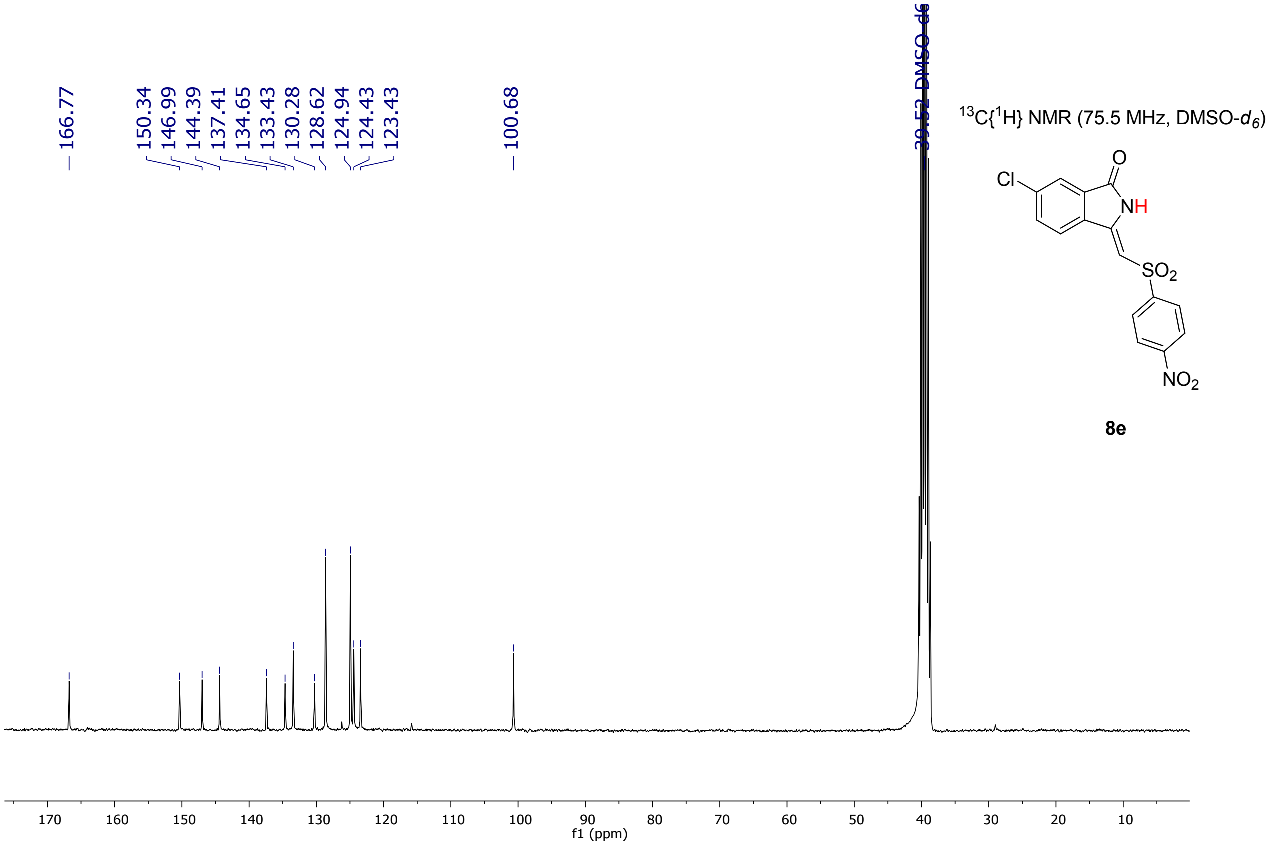

^{13}C{^{1}H} NMR (75.5 MHz, DMSO-d_6)
166.77
150.34
146.99
144.39
137.41
134.65
133.43
130.28
128.62
124.94
124.43
123.43
100.68
39.52 DMSO-d6
Cl
O
NH
SO_2
NO_2
f1 (ppm)

8e

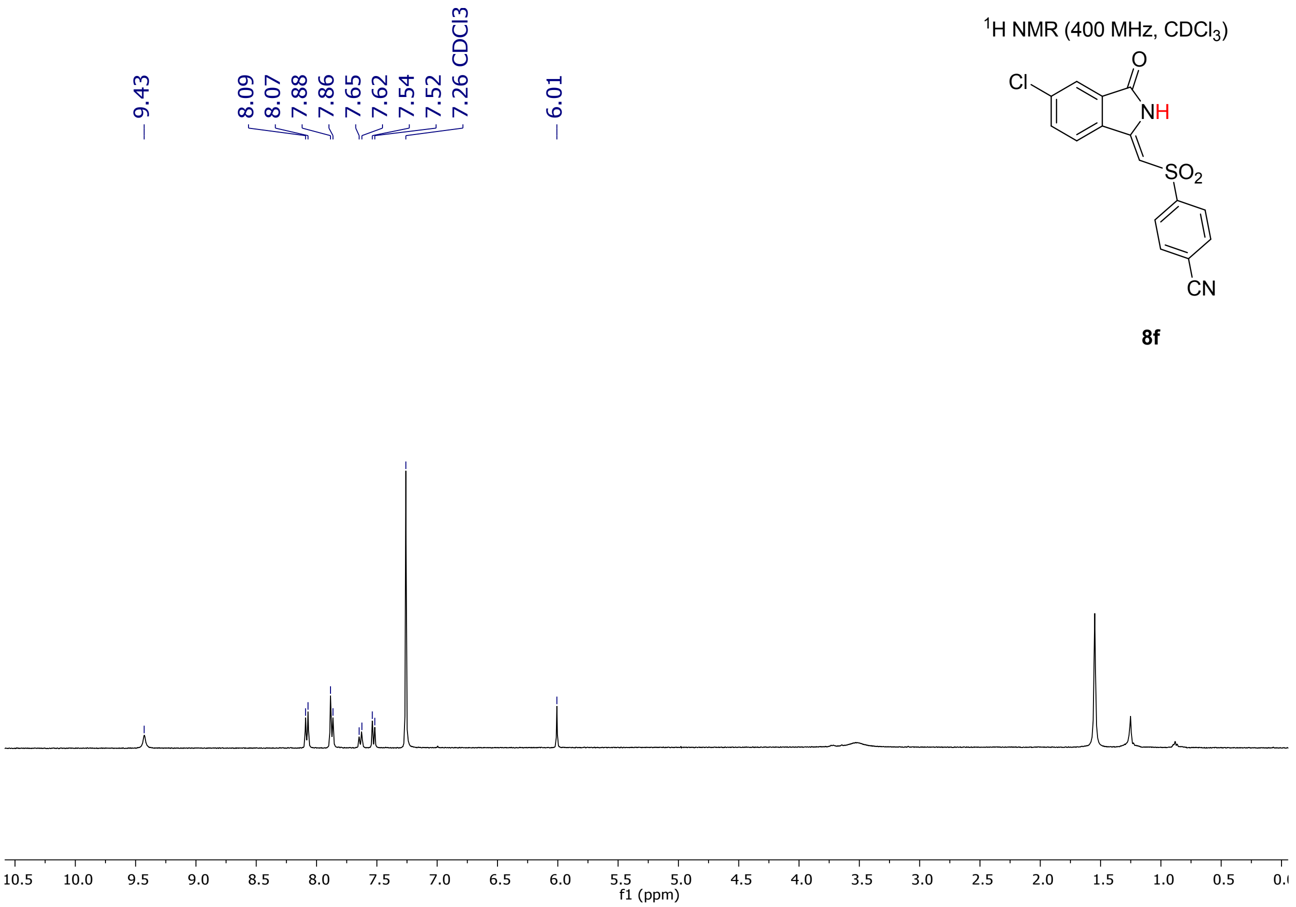

9.43
8.09
8.07
7.88
7.86
7.65
7.62
7.54
7.52
7.26 CDCl3
6.01
^{1}H NMR (400 MHz, $CDCl_3$)
Cl
O
NH
SO2
CN
8f
f1 (ppm)

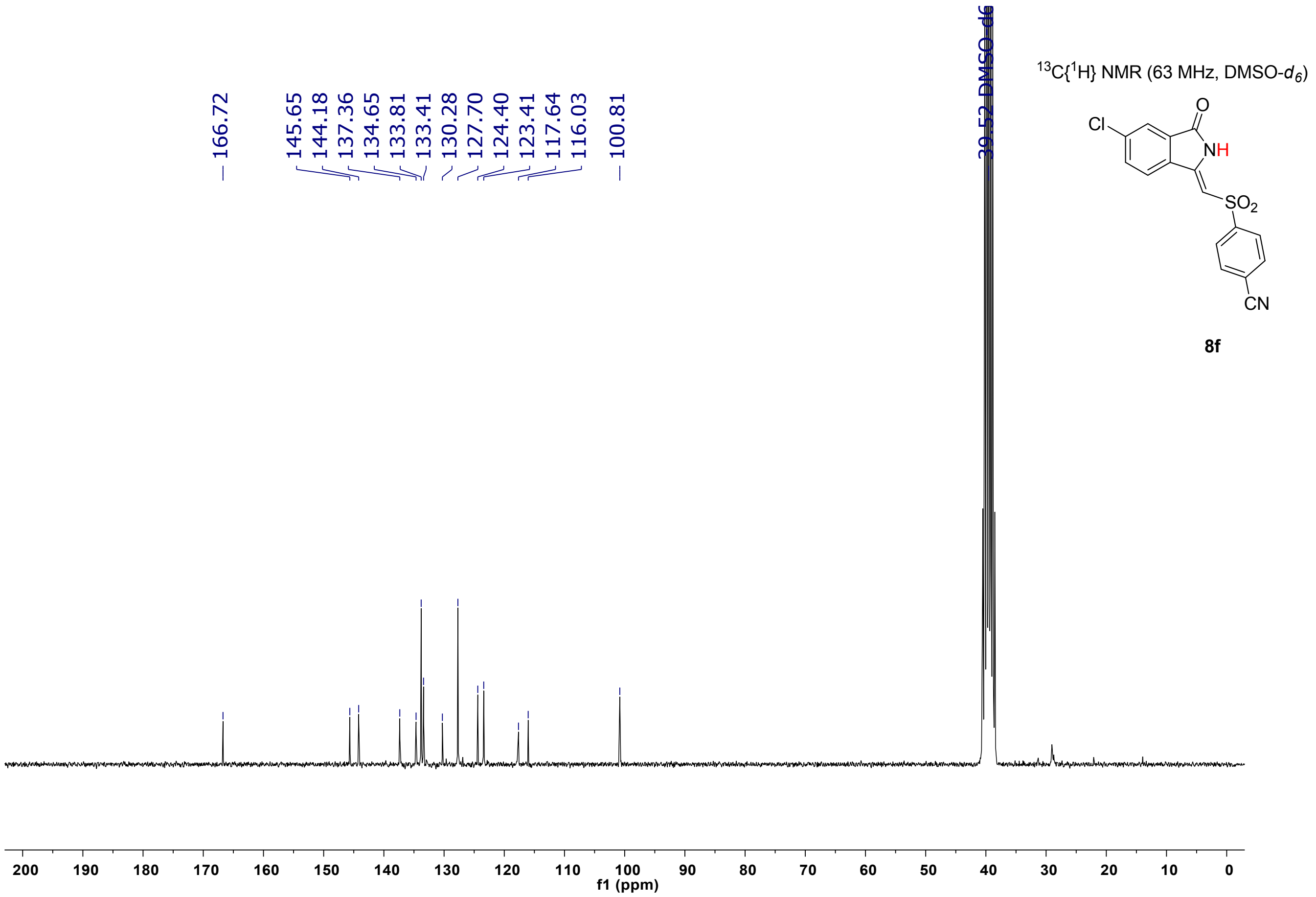

166.72
145.65
144.18
137.36
134.65
133.81
133.41
130.28
127.70
124.40
123.41
117.64
116.03
100.81
39.52 DMSO-d6
$^{13}C\{^{1}H\}$ NMR (63 MHz, DMSO-d_6)
Cl
O
NH
SO2
CN
8f
f1 (ppm)

^{1}H NMR (300 MHz, DMSO-d_6)

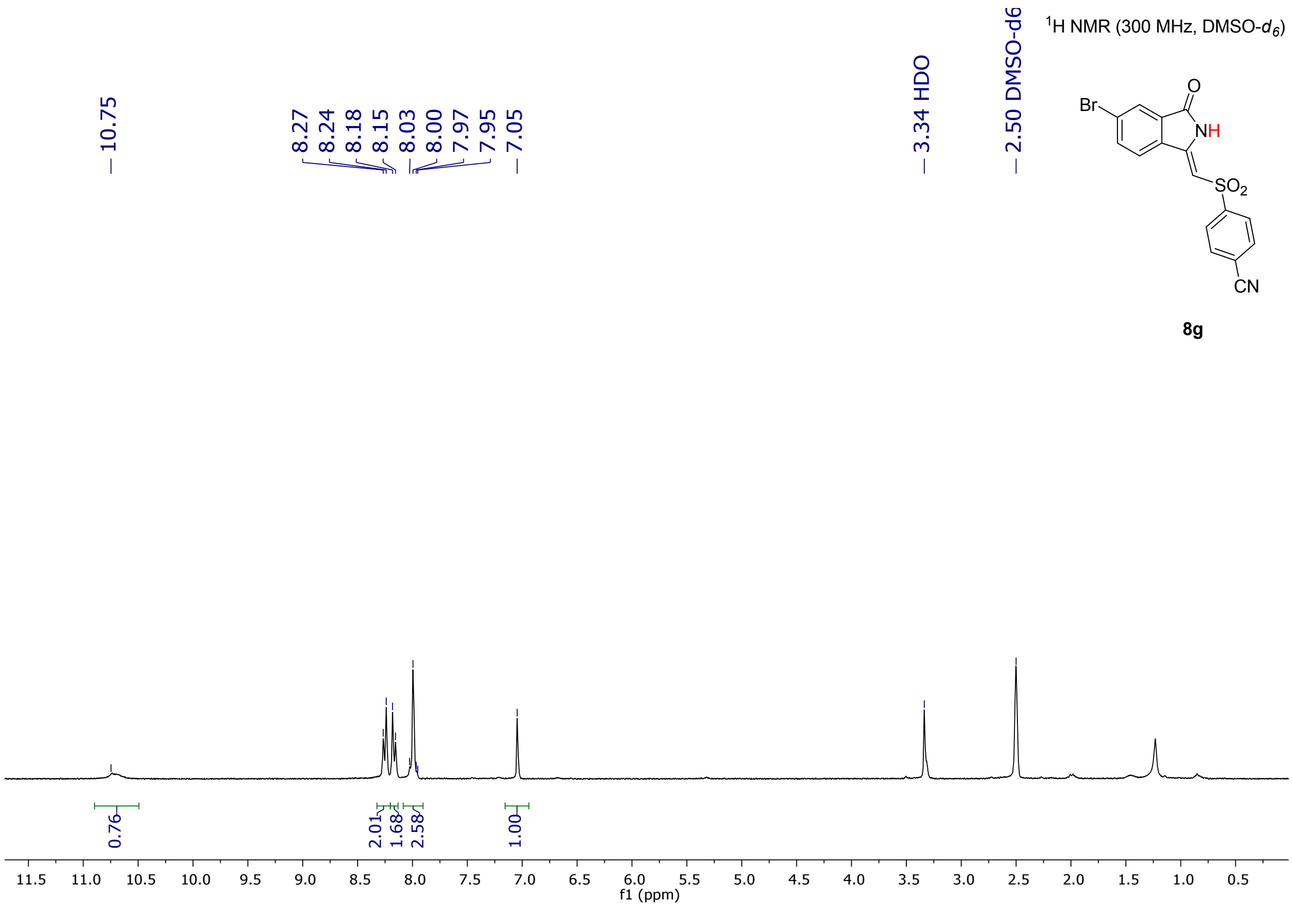

8g

$^{13}C\{^{1}H\}$ NMR (100.6 MHz, DMSO-d_6)

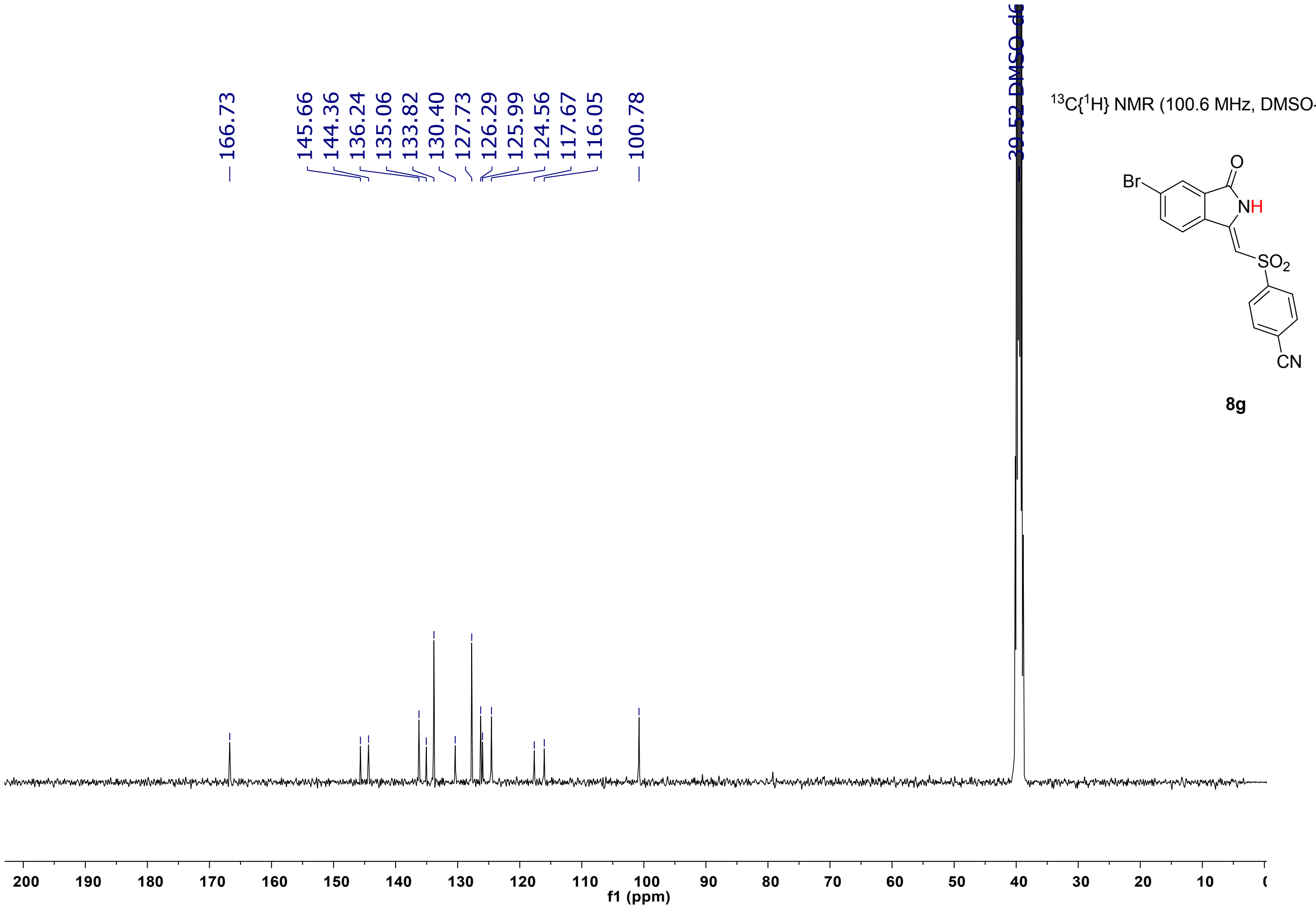

8g

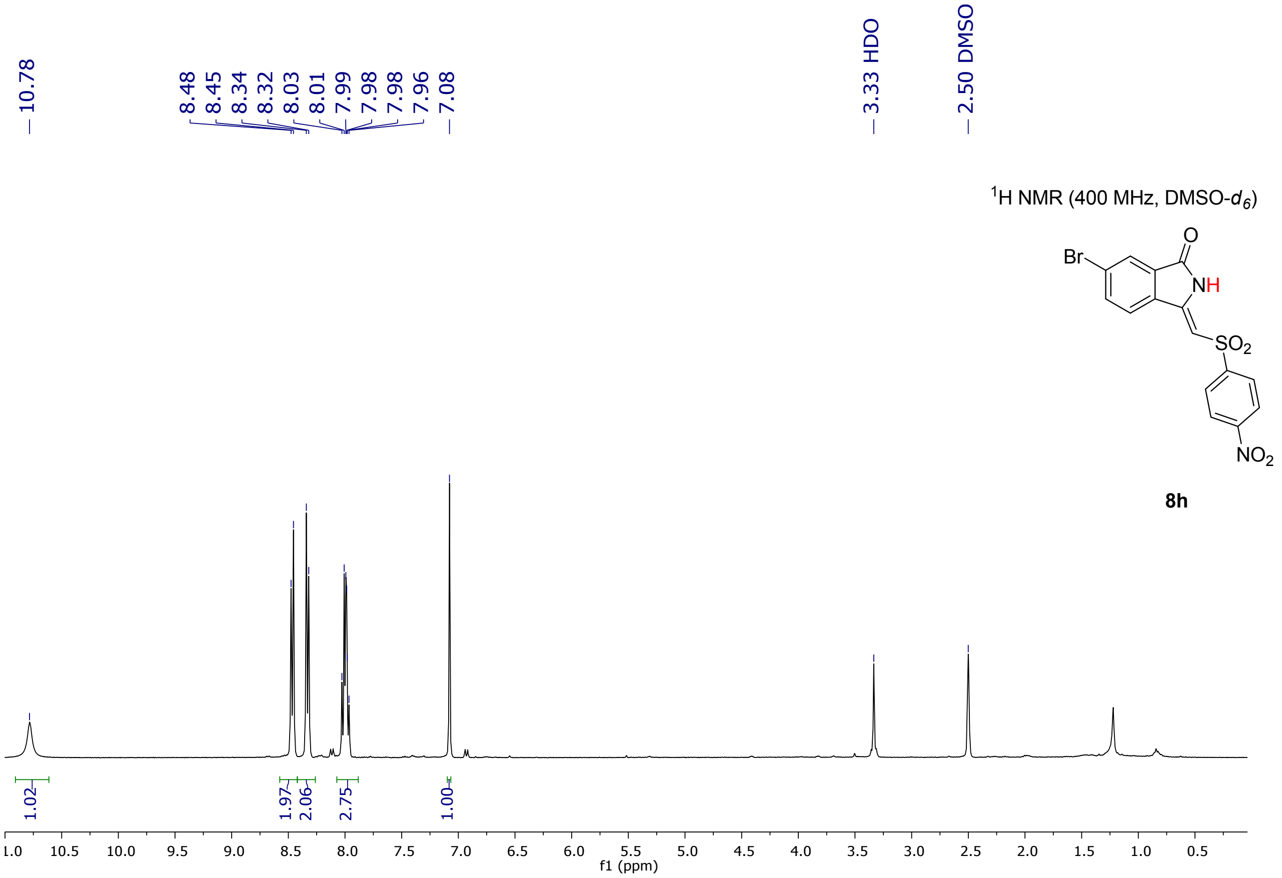

^{1}H NMR (400 MHz, DMSO-d_6)

8h

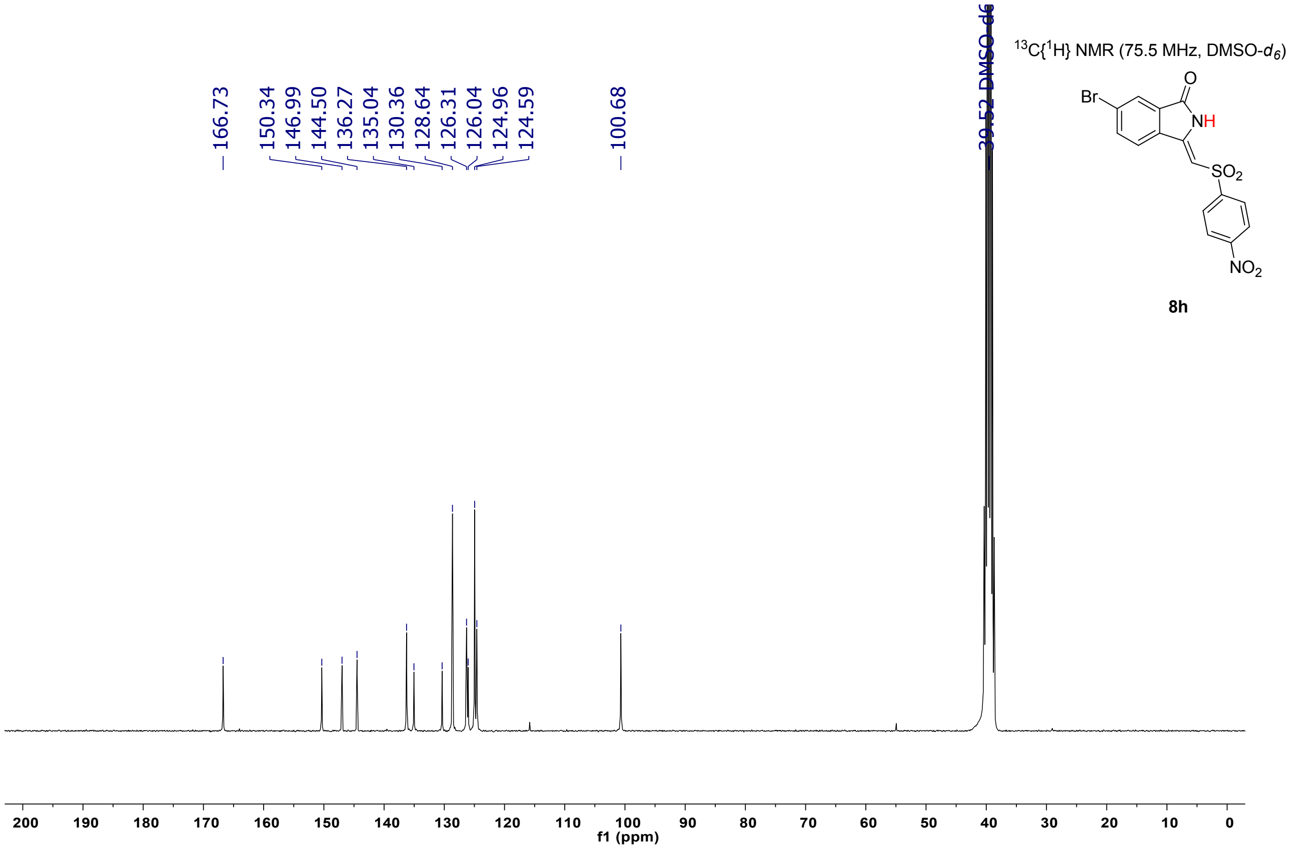

$^{13}C\{^{1}H\}$ NMR (75.5 MHz, DMSO-d_6)

8h

1.3. Copies of ^{1}H and $^{13}C\{^{1}H\}$ NMR of N-Alkylated (*Z, E*)-3 ((Phenylsulfonyl) methylene)isoindolin-1-ones

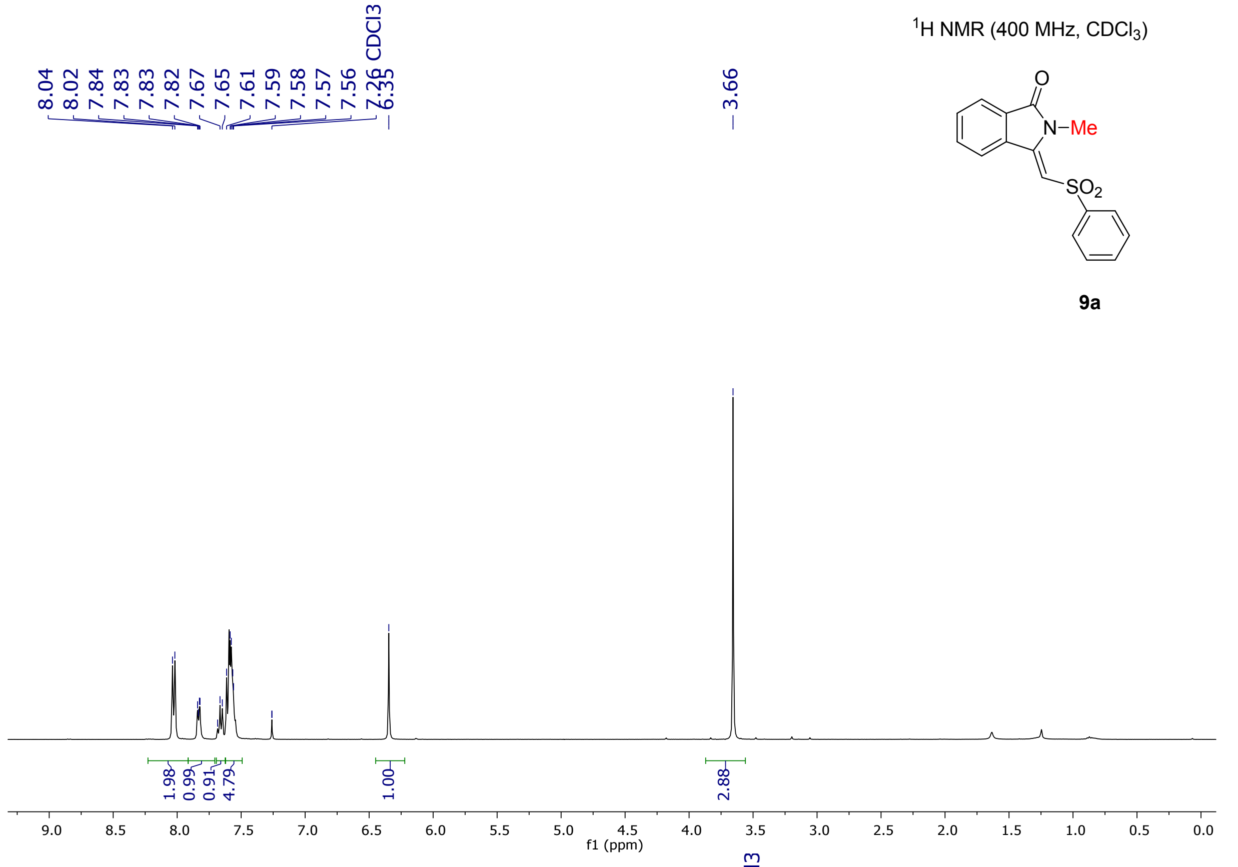

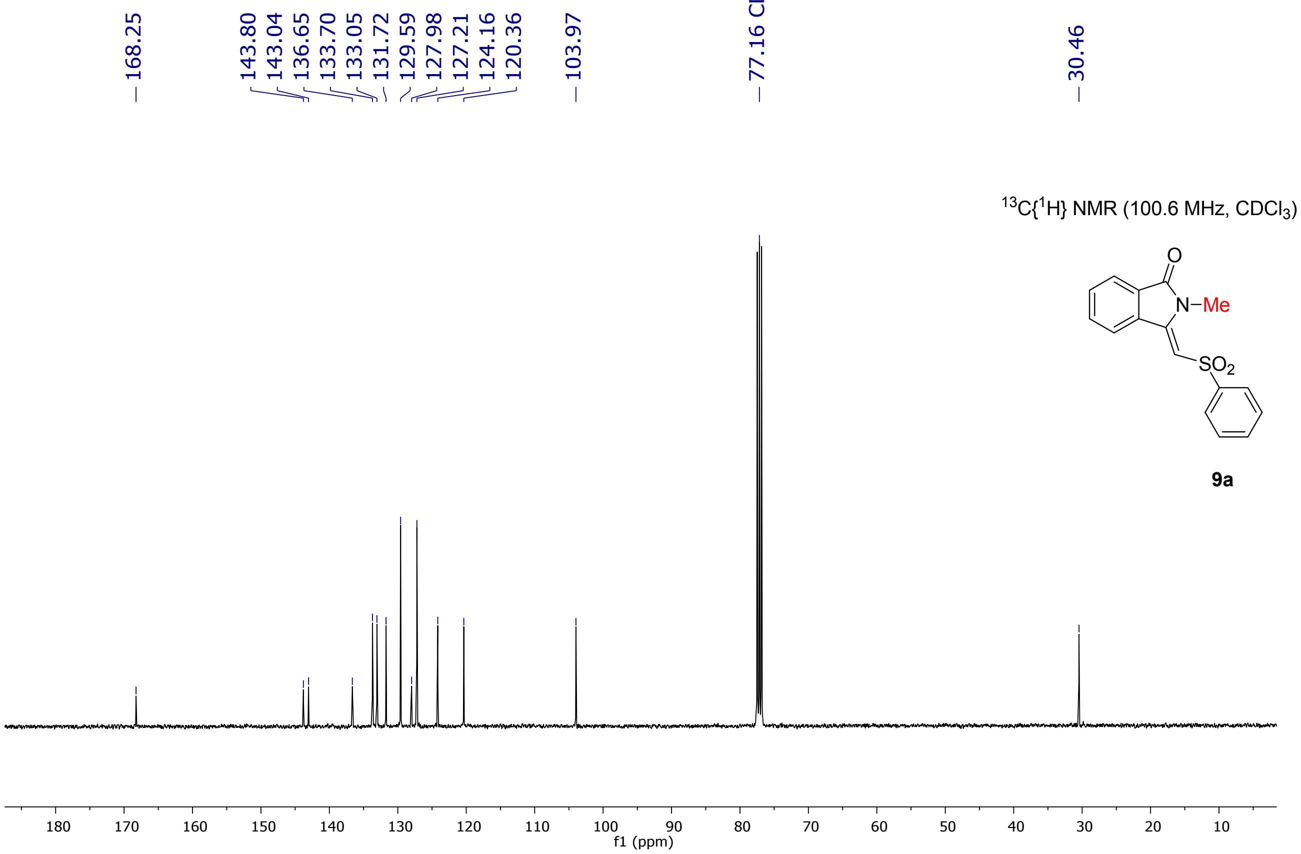

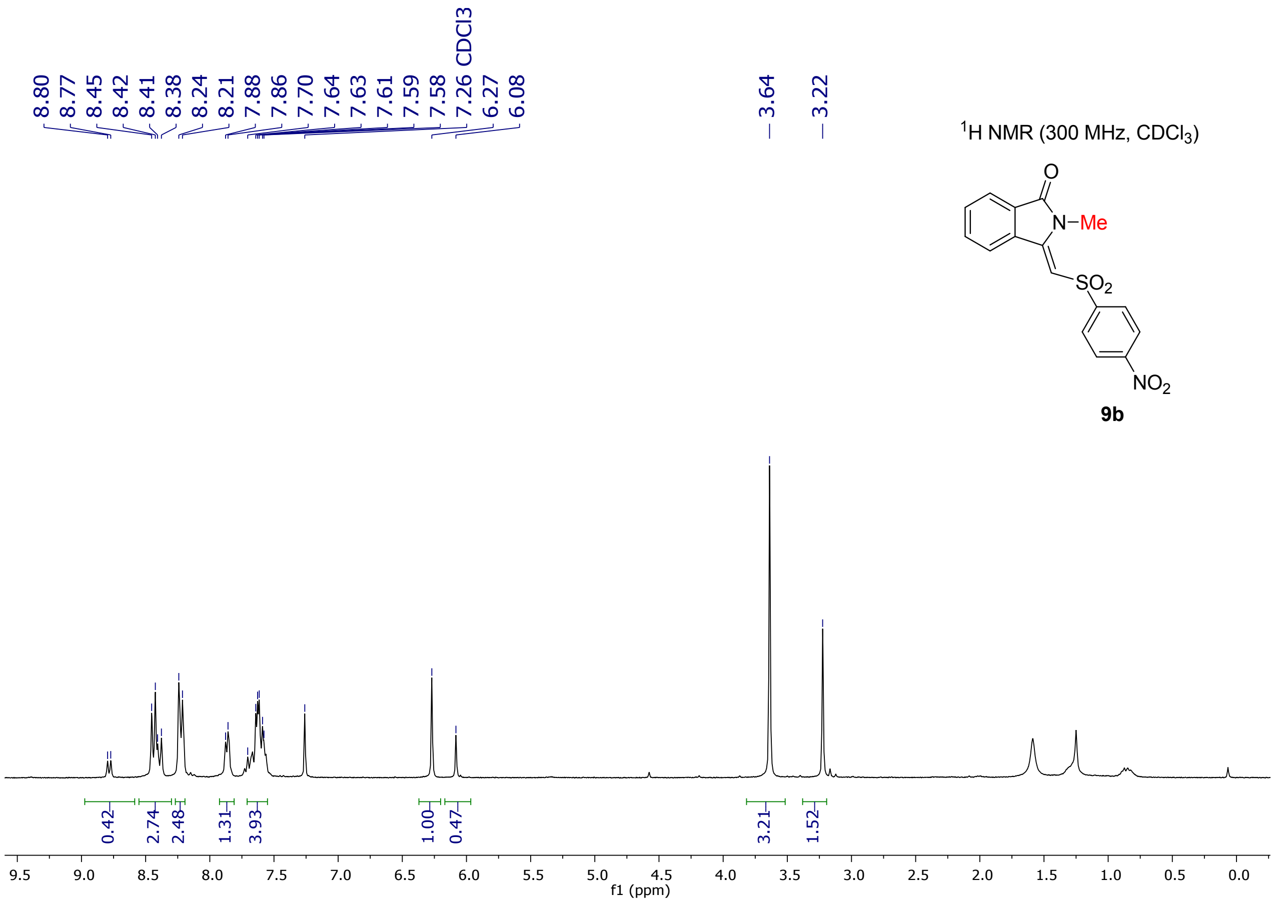
8.80
8.77
8.45
8.42
8.41
8.38
8.24
8.21
7.88
7.86
7.70
7.64
7.63
7.61
7.59
7.58
7.26 CDCl3
6.27
6.08
3.64
3.22
^{1}H NMR (300 MHz, $CDCl_3$)
O
N–Me
SO_2
NO_2
9b
0.42
2.74
2.48
1.31
3.93
1.00
0.47
3.21
1.52
9.5
9.0
8.5
8.0
7.5
7.0
6.5
6.0
5.5
5.0
4.5
4.0
3.5
3.0
2.5
2.0
1.5
1.0
0.5
0.0
f1 (ppm)

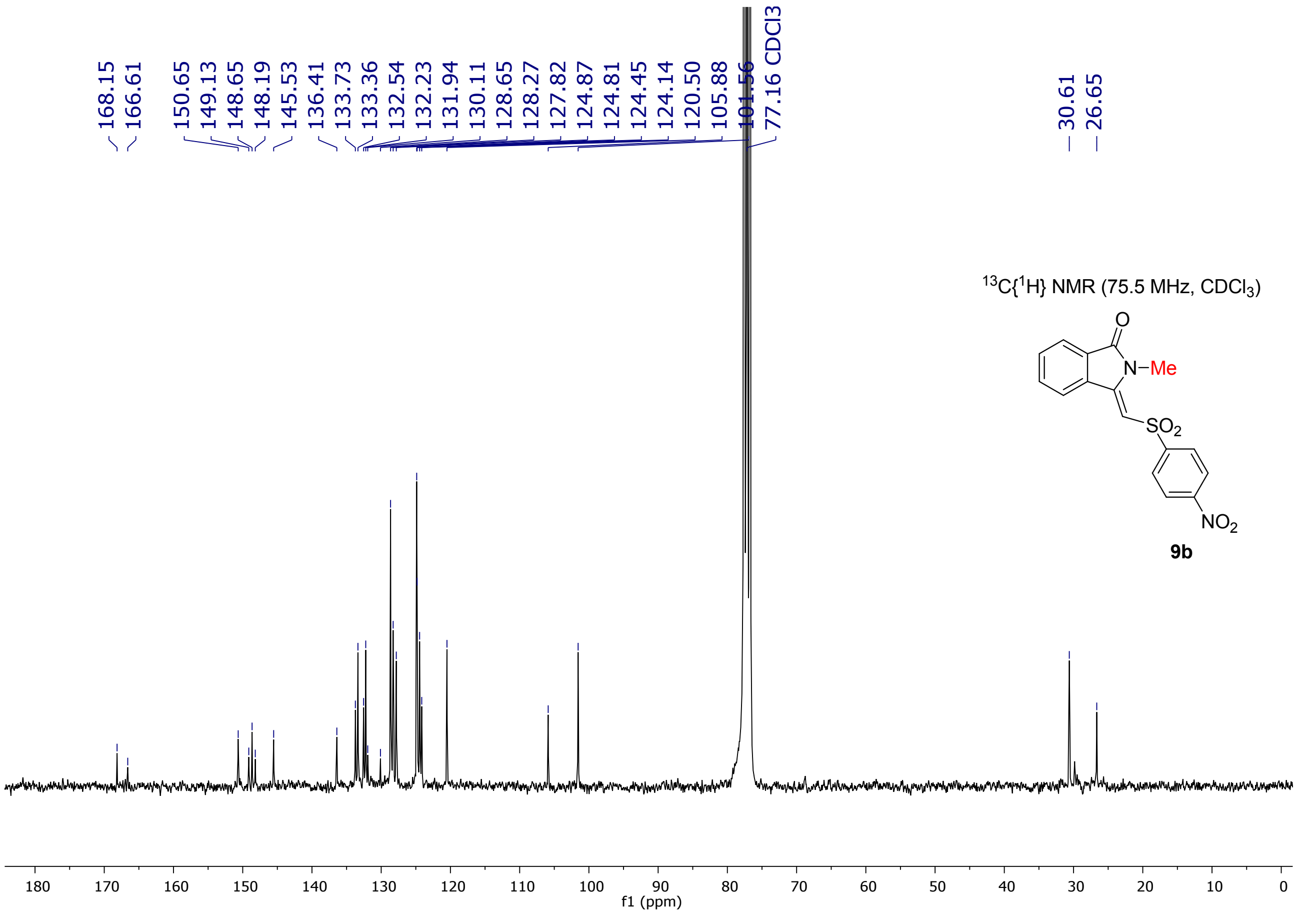
168.15
166.61
150.65
149.13
148.65
148.19
145.53
136.41
133.73
133.36
132.54
132.23
131.94
130.11
128.65
128.27
127.82
124.87
124.81
124.45
124.14
120.50
105.88
101.56
77.16 CDCl3
30.61
26.65
$^{13}C\{^{1}H\}$ NMR (75.5 MHz, $CDCl_3$)
O
N–Me
SO_2
NO_2
9b
180
170
160
150
140
130
120
110
100
90
80
70
60
50
40
30
20
10
0
f1 (ppm)

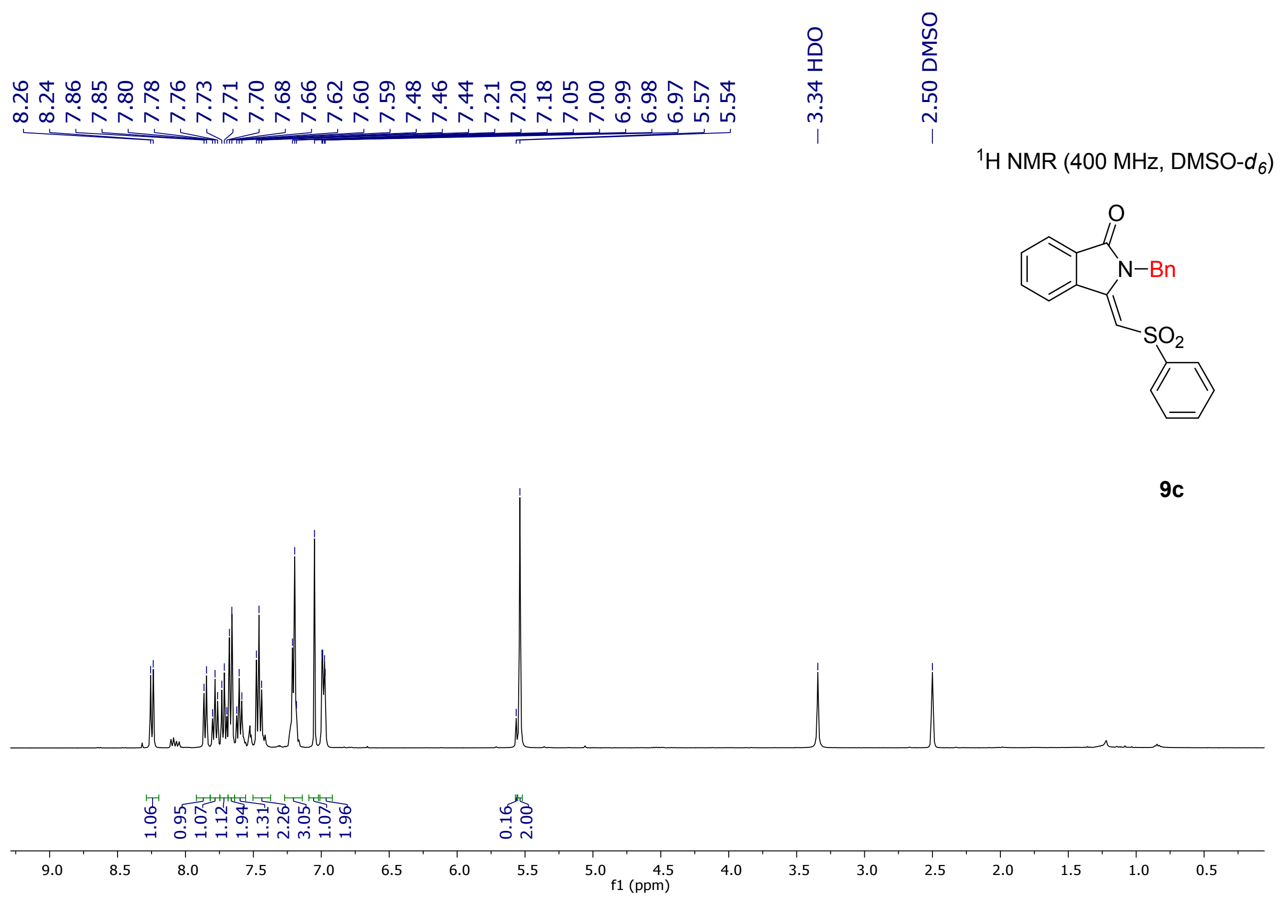
8.26 8.24 7.86 7.85 7.80 7.78 7.76 7.73 7.71 7.70 7.68 7.66 7.62 7.60 7.59 7.48 7.46 7.44 7.21 7.20 7.18 7.05 7.00 6.99 6.98 6.97 5.57 5.54
3.34 HDO
2.50 DMSO
1H NMR (400 MHz, DMSO-d6)
O
N–Bn
SO2
9c
1.06 0.95 1.07 1.12 1.94 1.31 2.26 3.05 1.07 1.96
0.16 2.00
f1 (ppm)

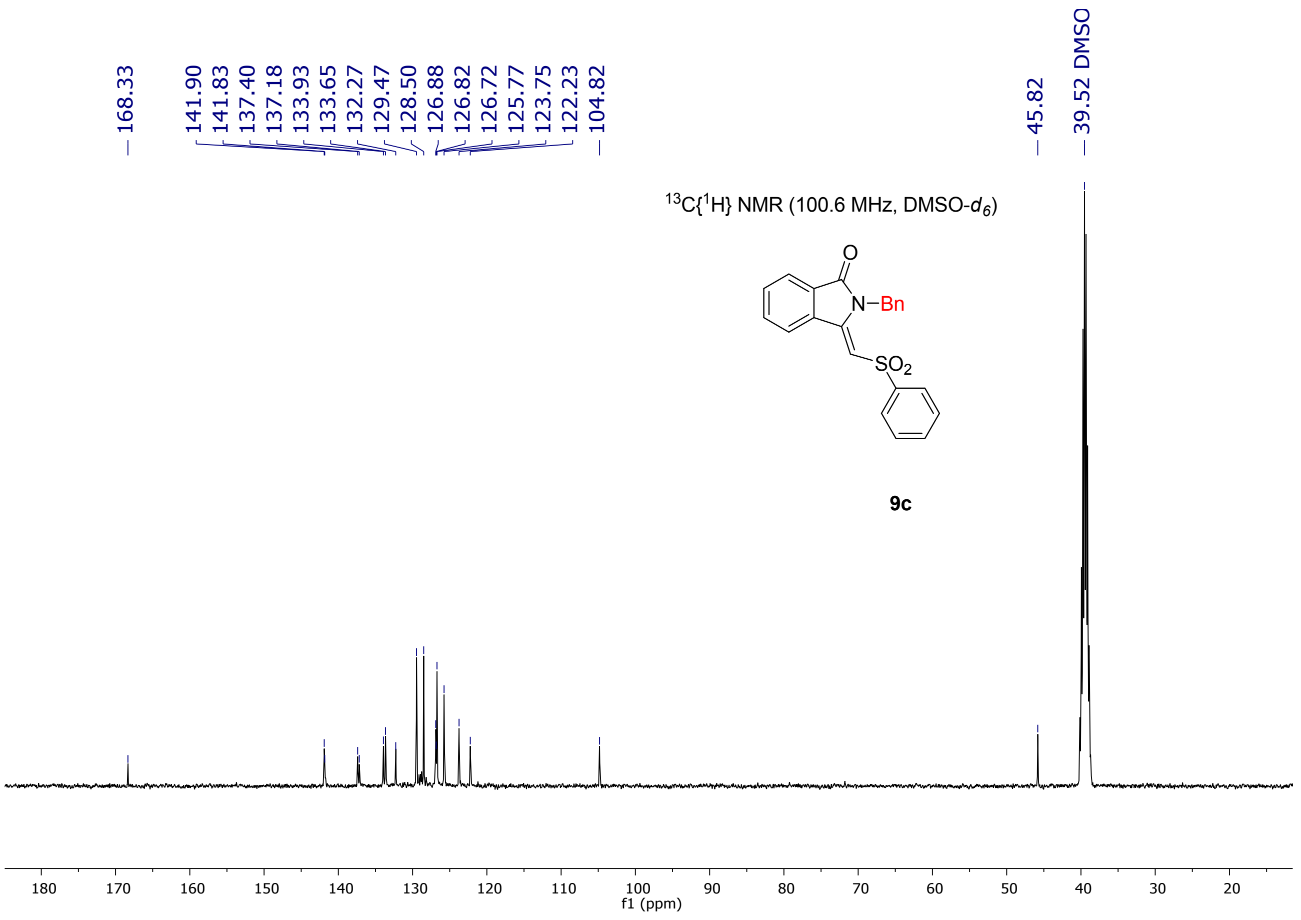
168.33
141.90 141.83 137.40 137.18 133.93 133.65 132.27 129.47 128.50 126.88 126.82 126.72 125.77 123.75 122.23 104.82
45.82
39.52 DMSO
13C{1H} NMR (100.6 MHz, DMSO-d6)
O
N–Bn
SO2
9c
f1 (ppm)

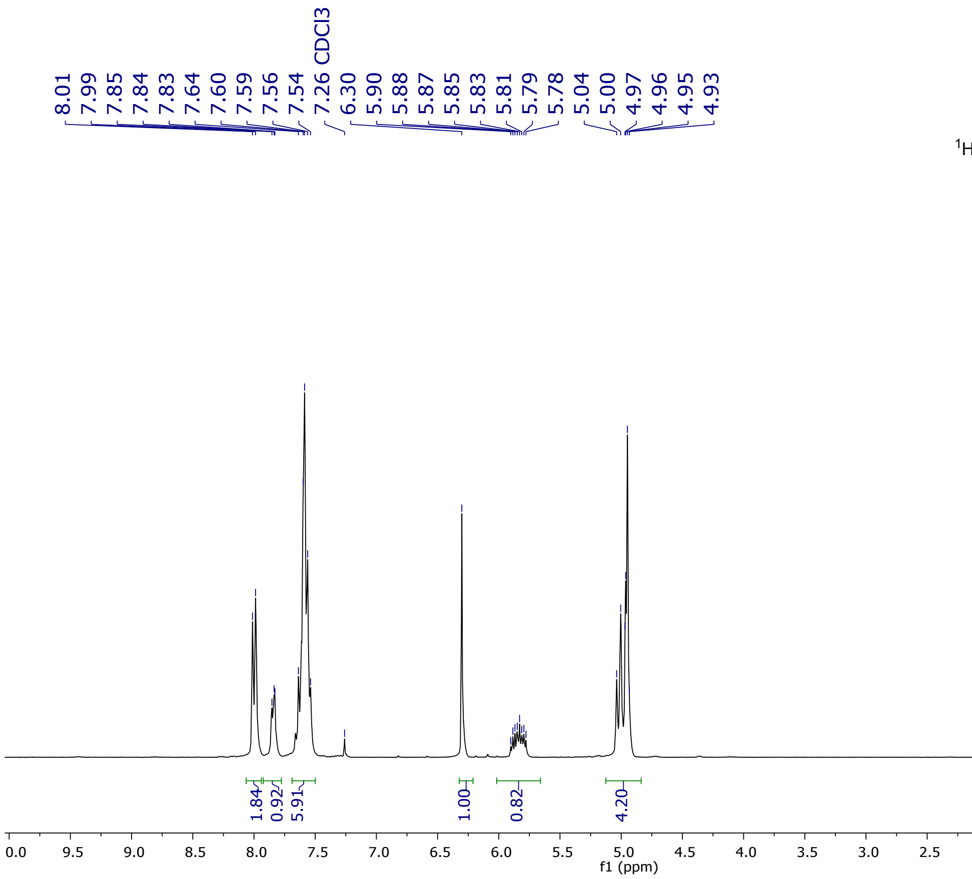

^{1}H NMR (300 MHz, $CDCl_3$)

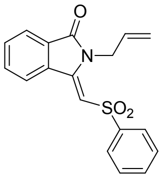

9d

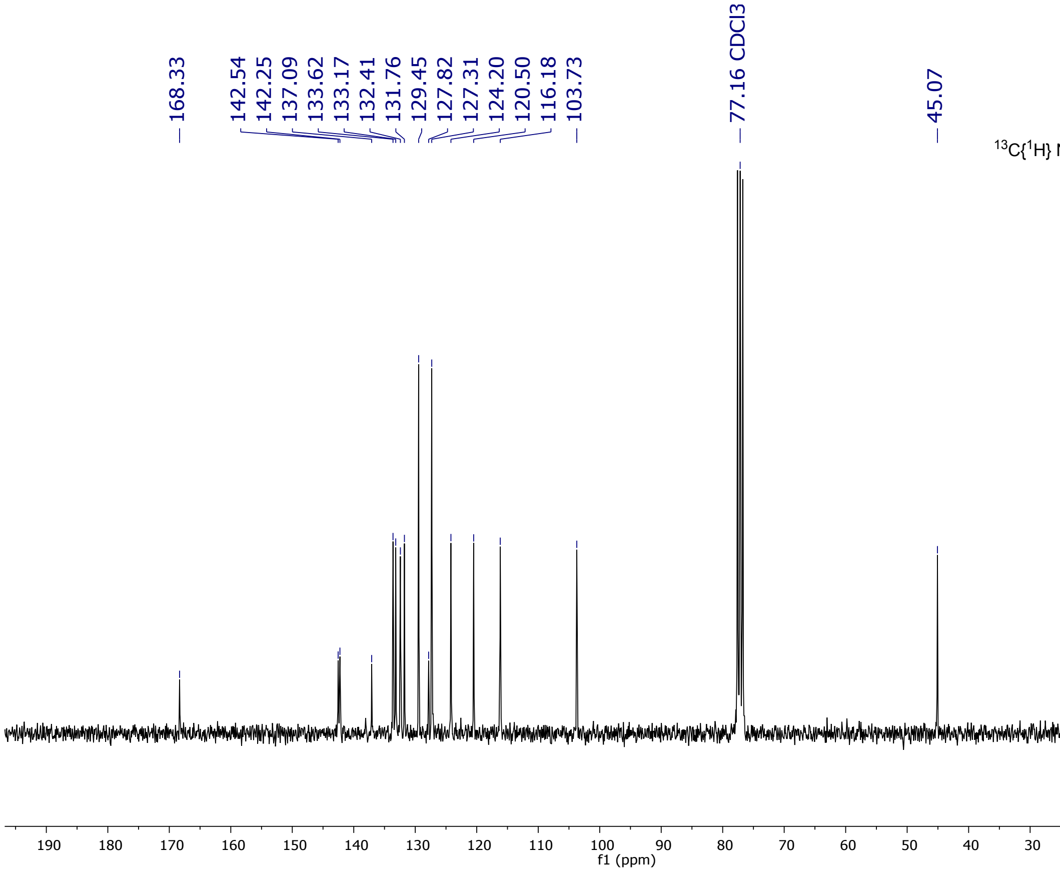

$^{13}C\{^1H\}$ NMR (75.5 MHz, $CDCl_3$)

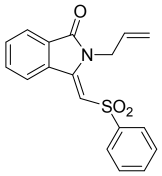

9d

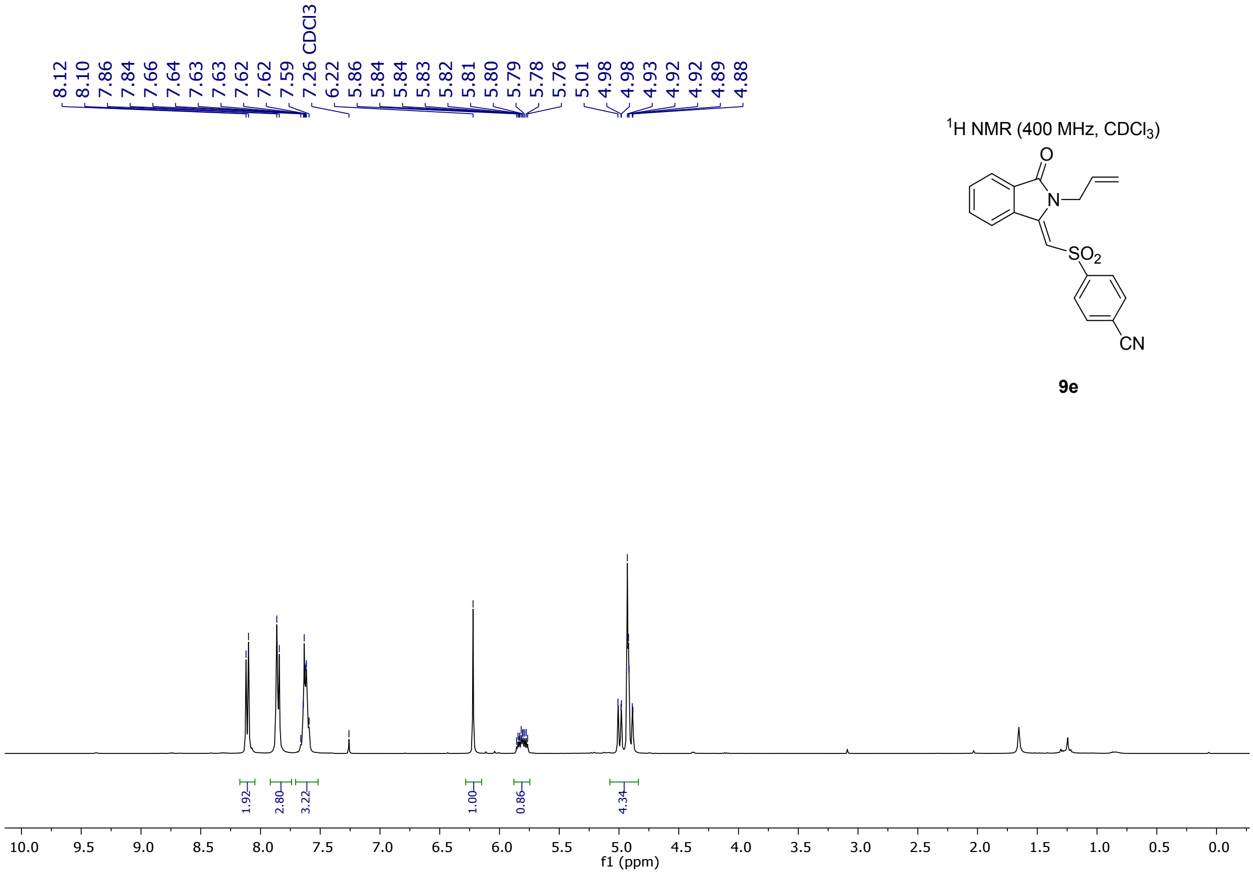
8.12 8.10 7.86 7.84 7.66 7.64 7.63 7.63 7.62 7.62 7.59 7.26 CDCl3 6.22 5.86 5.84 5.84 5.83 5.82 5.81 5.80 5.79 5.78 5.76 5.01 4.98 4.98 4.93 4.92 4.92 4.89 4.88
1H NMR (400 MHz, CDCl3)
O
N
SO2
CN
9e
1.92 2.80 3.22 1.00 0.86 4.34
f1 (ppm)

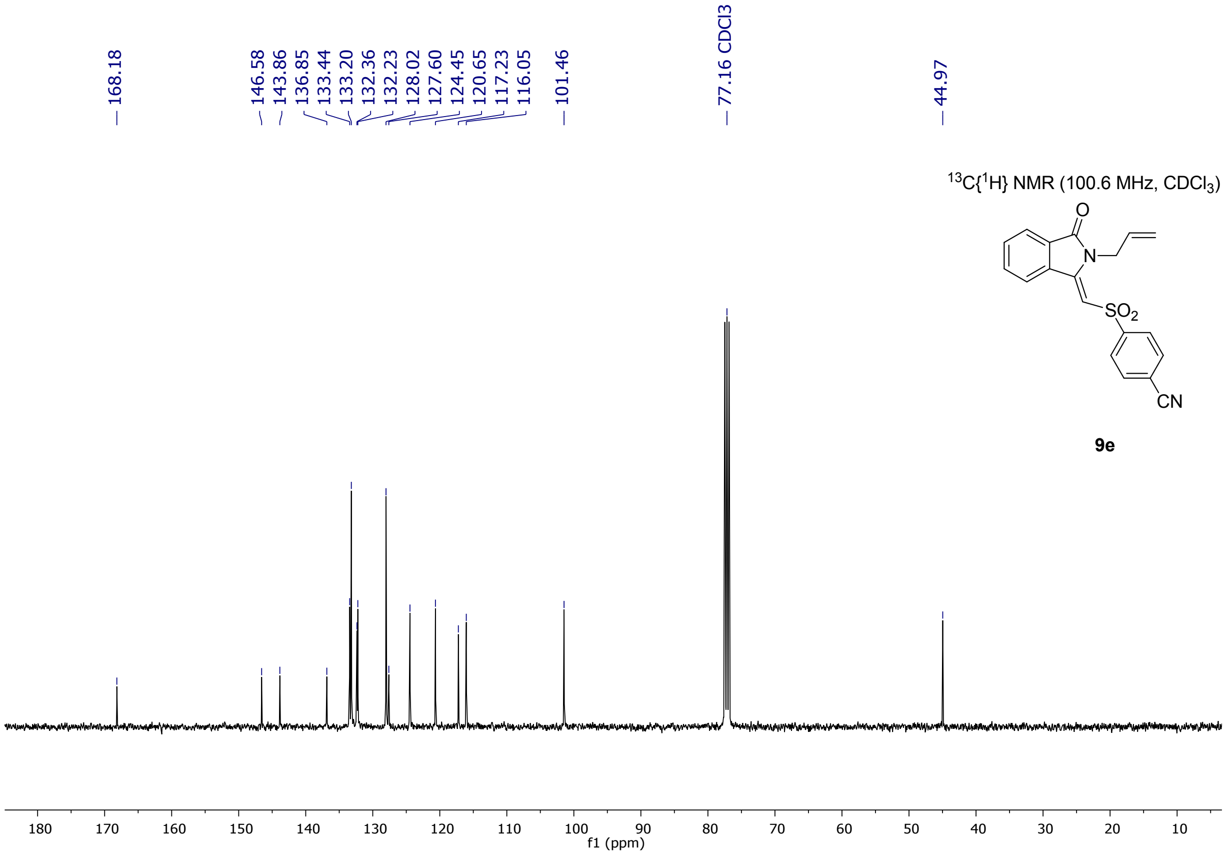
168.18 146.58 143.86 136.85 133.44 133.20 132.36 132.23 128.02 127.60 124.45 120.65 117.23 116.05 101.46 77.16 CDCl3 44.97
13C{1H} NMR (100.6 MHz, CDCl3)
O
N
SO2
CN
9e
f1 (ppm)

^{1}H NMR (400 MHz, $CDCl_3$)

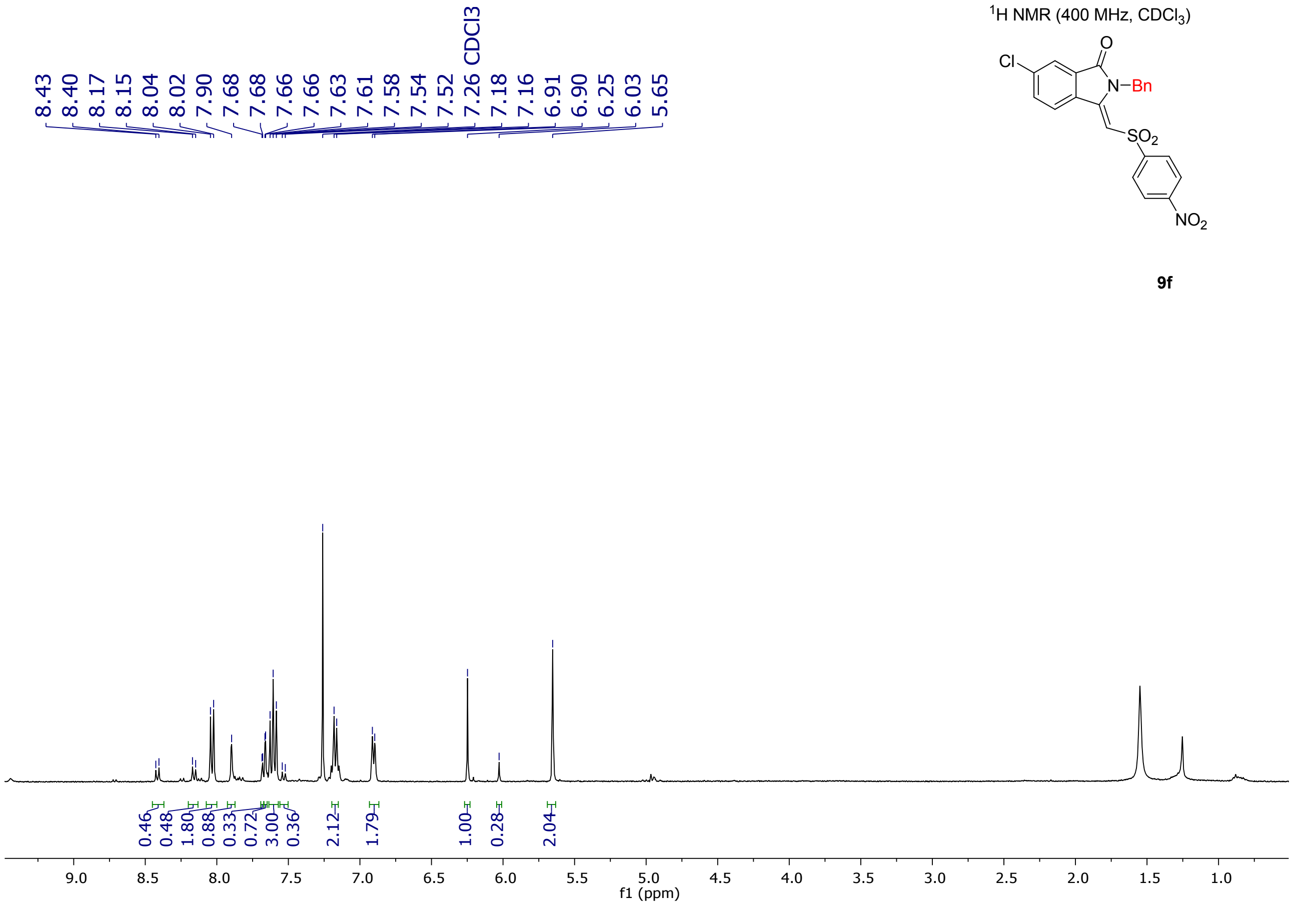

9f

^{13}C{^{1}H} NMR (100.6 MHz, DMSO-d_6)

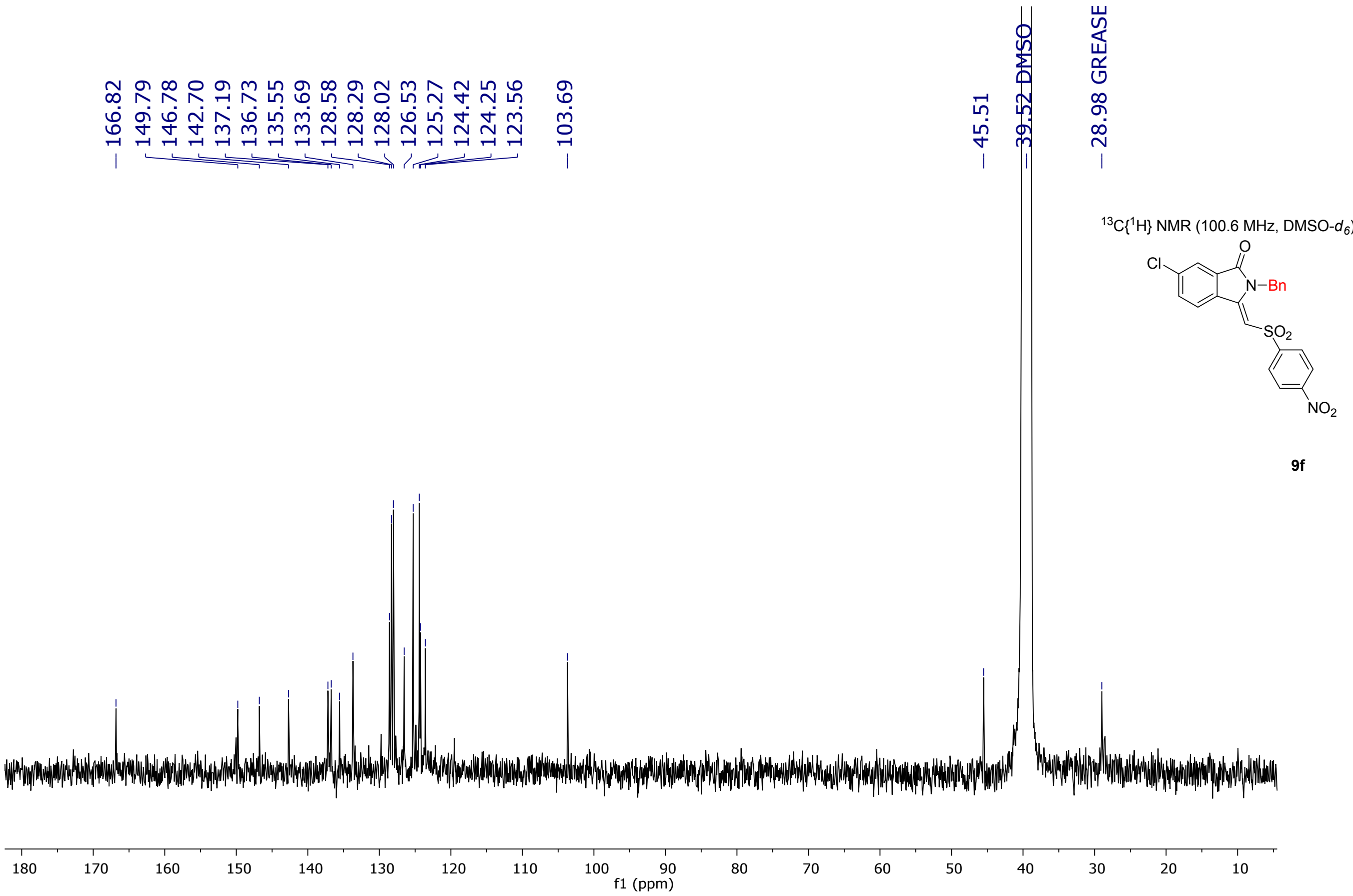

9f

2. X-ray crystallography

Colorless prismatic single crystals of compound **AMA277 (7a)** suitable for X-ray diffraction analysis were obtained by slow evaporation of a solution of **7a** by dissolving 5 mg of the compound in 0.6 ml of hexane/AcOEt (2/1).

A crystal of 0.57 mm x 0.23 mm x 0.11 mm was selected and mounted on a nylon loop with paratone oil and measured at room temperature with a Bruker D8 QUEST diffractometer equipped with a PHOTON II detector using Cu*Kα* radiation (λ= 1.54178 Å).

Data Indexing was performed using APEX3 software.[1] Data integration and reduction were performed using SAINT.[2] Absorption correction was performed by multi-scan method in SADABS.[2] The structure was solved using SHELXS-97[3] and refined by means of full matrix least-squares based on F^3 using the program SHELXL.[4] Non-hydrogen atoms were refined anisotropically, while hydrogen atoms were positioned geometrically and included in structure factors calculations but not refined. ORTEP diagram (**Figure S1**) was drawn using OLEX2.[5]

Crystallographic data are reported in **Table S1**.

CCDC- 2087404 contains the supplementary crystallographic data for this paper. These data can be obtained free of charge from The Cambridge Crystallographic Data Centre via www.ccdc.cam.ac.uk/structures

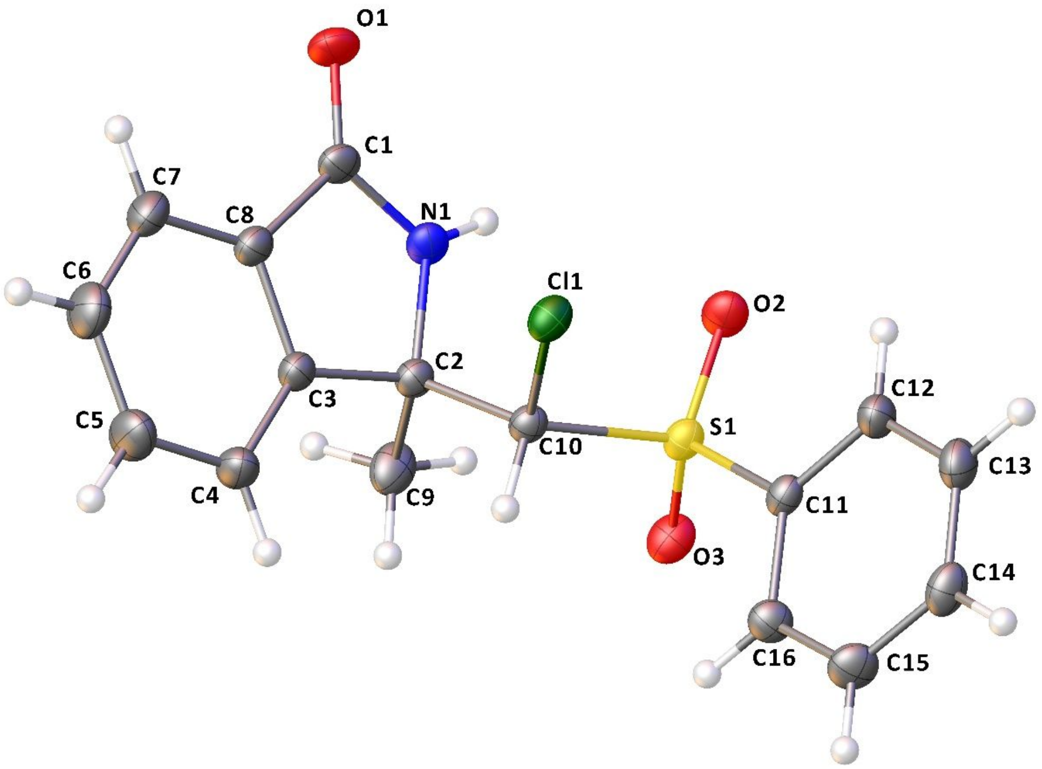

Figure.S1 ORTEP diagram for compound **ama277 (7a)**. Atom types: C grey, O red, H white, N blue, S yellow, Cl green. Ellipsoids are drawn at 20% probability level.

Table S1. Crystallographic data for compounds **ama277 (7a)**

	**ama277**
**T (K)**	296
**Formula**	$C_{16}H_{14}ClNO_3S$
**Formula weight**	335.79
**System**	Monoclinic
**Space group**	*C*2/c
a **(Å)**	18.123(4)
b **(Å)**	15.625(3)
c **(Å)**	13.992(4)
β **(°)**	128.262(12)
V **(Å^3)**	3111.1(13)
**Z**	8
Dx **(g cm^{-3})**	1.434
**λ (Å)**	1.54178
μ **(mm^{-1})**	3.534
**F_{000}**	1392
**R1 (I > 2σI)**	0.0345(2922)
**$_wR_2$**	0.0978(3058)
**N. of param.**	205
**GooF**	1.054
**ρ_{min}, ρ_{max} (eÅ^{-3})**	-0.337, 0.271

3. DFT Investigation

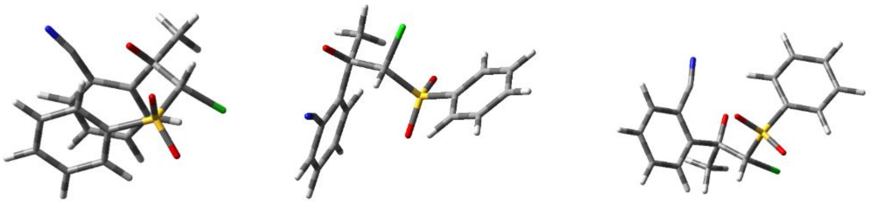

(R,S)-**4a**-CTC (R,S)-**4a**-TG$^-$C (R,R)-**4a**-G$^-$G$^-$C

ΔE=0.0, ΔG=0.0 ΔE=11.8, ΔG=5.7 ΔE=5.8, ΔG=3.0

Figure S1. Minimum energy conformers of (R,S)- and (R,R)-configured **4a**.

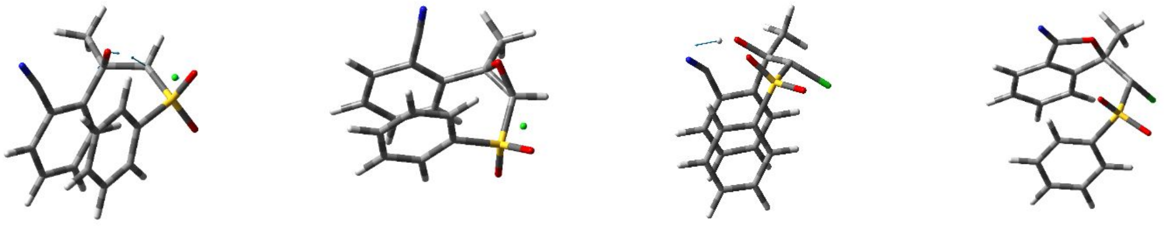

[(R,S)-**4a**-CTC]‡ **5**·Cl$^-$ (R,*)-**4b**‡-CTC (R,*)-**6ab**-CTC

$\Delta E^‡$=28.8, $\Delta G^‡$=25.4 ΔE=-94.0, ΔG=-100.7 $\Delta E^‡$=243.9 $\Delta G^‡$=229.0

Figure S2. Routes to epoxide

Conformer	Starting Structure	Ending Structure	Energy Scan
(R,R)-**4a**-G$^-$G$^-$C	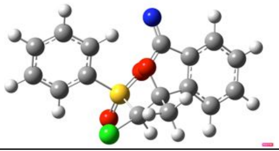	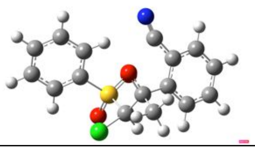	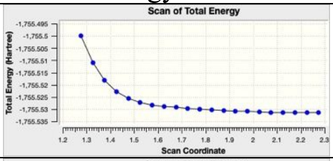
(R,S)-**4a**-CTC	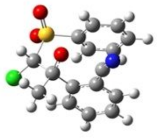	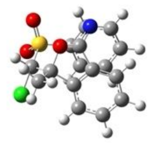	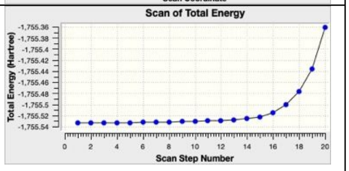

Table S2. Energy relaxed scans of two representative conformers of **4a** for the 5-membered cyclization. Energy has been computed at the PCM/APFD/cc-pVDZ.

Table S3. Results of the conformational analysis on halohydrinate **4a** performed in gas phase at the APFD/aug-cc-pVDZ level with a single point correction at the PCM level (free energy corrections coming from gas-phase). Energies in kJ mol^{-1}. The dihedral angles θ_1, θ_2 and θ_3 are defined in the text.

Configuration	Conformation	θ_1	θ_2	θ_3	ΔE	ΔG
(R,S)-**4a**	CTC	-5.50	-161.60	25.90	0.00	6.18
	G-TC	-80.10	-180.00	4.00	2.95	7.25
	TG-C	172.20	-67.20	-27.00	3.00	0.00
	TG+C	164.10	41.20	-4.50	10.37	8.29
	TG-C	172.20	-67.20	-26.90	10.38	7.45
	G+G+T	74.30	73.10	-178.30	14.60	13.75
	G-TT	-64.40	170.80	168.00	15.49	15.68
	TG-A	-174.80	-59.60	147.00	21.92	18.19
	G-G+C	-83.60	45.00	-3.20	23.01	22.44
	TTT	158.00	170.30	169.80	29.32	28.84
	CG-C	-22.50	-51.20	-2.30	51.40	36.83
(R,R)-**4a**	G-G-C	-74.60	-72.80	3.30	2.99	7.40
	G+TC	76.70	-170.00	-3.60	9.54	10.05
	G-TC	-82.60	-171.50	-11.70	11.26	12.87
	G+G-T	45.20	-63.50	178.90	11.99	12.99
	TTC	-179.40	-172.10	-6.00	14.61	12.19
	TG-T	159.30	-58.70	-178.50	15.65	14.24
	T+G+C	166.90	72.00	-2.40	15.86	12.35
	CTC	-27.90	-152.60	-17.60	16.18	17.59
	T-G+C	-159.20	67.30	-16.30	19.71	17.53

The further deprotonation of **4b** can occur in the first stages of the reaction in case of stoichiometric amount of *tert*-butoxide (Reaction 1 in Table S2), it would be poorly or not at all efficient in case of the carbonate or the hydrogenocarbonate ions, reactions 3 and 4 in Scheme S1, respectively. However, the small endoergicity of the proton abstraction by the carbonate can be better circumvented at equilibrium by an increase of temperature and with more acidic ((chloromethyl)sulfonyl)benzenes substituted with EWGs, allowing the reactions to go to completion in the other cases (Scheme 2). The *ter*-butoxide would have a catalytic role as it would be regenerated by protonation of (R,*)-**6ab**-CTC to yield (R,*)-**6b**-CTC (See also Figure 3, of main manuscript).]

**Table S4.** Reactions promoted by KO*t*Bu vs K_2CO_3	ΔE	ΔG
1. (R,*)-**4b**-CTC + **3** → (R,*)-**6ab**-CTC + **3H**	-16.12	-12.75
2. (R,*)-**6ab**-CTC + **3H** → (R,*)-**6b**-CTC + **3**	-39.9	-36.8
3. (R,*)-**4b**-CTC + CO_3^{2-} → (R,*)-**6ab**-CTC + HCO_3^-	1.77	1.39
4. (R,*)-**4b**-CTC + HCO_3^- → (R,*)-**6ab**-CTC + H_2CO_3	139.67	141.65

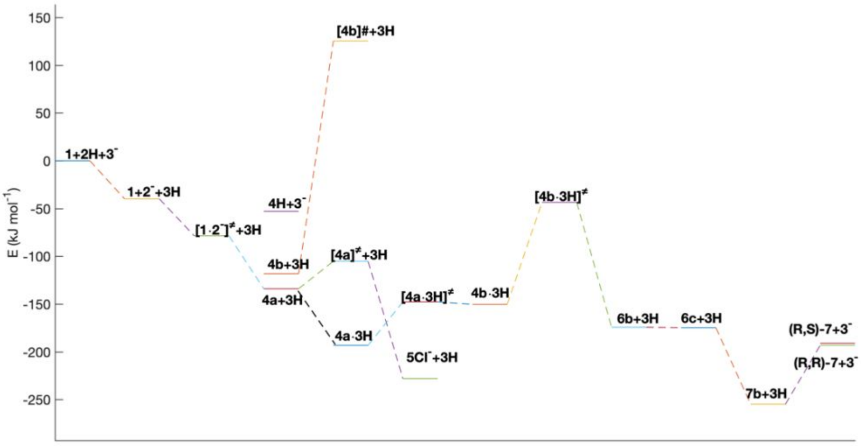

Figure S3. Profile of energies of relevant species in the studied system computed at the APFD/aug-cc-pVDZ using the PCM to describe the acetonitrile.

[4b]$^{\neq}$

C	0.24168200	1.63947400	0.05590800
C	-0.77944800	1.49835900	1.00467400
C	-2.09557300	1.87909200	0.74304300
C	-2.38138800	2.45924100	-0.49083700
C	-1.36331900	2.64001000	-1.43420800
C	-0.05306400	2.23259400	-1.17028700
C	1.58998200	1.06965900	0.51109000
H	-2.87263600	1.72984900	1.49230200
H	-3.39945300	2.77520700	-0.72027400
H	-1.59682800	3.10401600	-2.39387300
H	0.72321900	2.37594000	-1.91910600
C	2.03179600	-0.24112900	-0.16602700
O	1.16161400	-2.01082300	1.56517600
O	1.56942800	-2.74762900	-0.84590400
O	1.35236700	0.86164800	1.91747400
C	-0.31366600	0.81588700	2.19835000
N	-0.43588900	0.03925400	3.12903500
C	2.69091600	2.12121800	0.38479900
H	2.40726100	3.00994900	0.96259800
H	3.63271800	1.71573400	0.77359900
H	2.84011400	2.41726900	-0.65943900
S	1.08132200	-1.68502600	0.09815400
C	-0.69278600	-1.53190600	-0.24635600
C	-1.09436400	-1.07687000	-1.50462400
C	-1.62286700	-1.82963000	0.74721300
C	-2.45383300	-0.91535800	-1.76446100
H	-0.35420100	-0.83909000	-2.26676200

C	-2.98296100	-1.67380100	0.47278800
H	-1.27658500	-2.15566400	1.72553400
C	-3.39866300	-1.21555200	-0.77852300
H	-2.77788900	-0.54892200	-2.73948700
H	-3.71981000	-1.90457400	1.24381400
H	-4.46193500	-1.08566900	-0.98622500
Cl	2.30077500	0.00597500	-1.94538800
H	0.89099300	-0.06326200	2.61598500

1 imaginary frequency=	-2014.1344	cm^{-1}
Zero-point correction=	0.249419 (Hartree/Particle)	
Sum of electronic and zero-point Energies=	-1755.260115	
Sum of electronic and thermal Free Energies=	-1755.306734	

[4a]≠

C	2.48683900	-1.83341300	-0.54215100
C	1.50529600	-1.47989400	0.39787000
C	0.14837900	-1.42853700	0.03415300
C	-0.20033200	-1.76710000	-1.27353000
C	0.77307800	-2.11740400	-2.20897800
C	2.12115200	-2.14601600	-1.84694200
H	3.53323000	-1.85330700	-0.23887700
H	-1.25063200	-1.73158400	-1.56002600
H	0.47764300	-2.36186600	-3.23038000
H	2.88617800	-2.40988400	-2.57739700
C	1.96230400	-1.20241000	1.72919900
N	2.45330600	-1.05946400	2.77477000
C	-0.90244600	-1.05247800	1.06525200
O	-0.50678000	-0.07526000	1.95903900
C	-1.48699400	-2.31965600	1.68849300
H	-2.27452900	-2.05862700	2.40630200
H	-1.90193700	-2.98294400	0.91992500
H	-0.68506000	-2.84995800	2.21996200
C	-1.92249500	-0.04638900	0.67878400
H	-2.67837600	0.16648900	1.42880300
Cl	-3.50898800	-0.80498100	-0.68332400
S	-1.40112500	1.55351800	-0.09731800
O	-1.90751500	1.64810900	-1.49471000
O	-1.85811600	2.60143300	0.86480300
C	0.38730900	1.62416800	-0.21829800
C	0.96694300	1.34560000	-1.45335200
C	1.13628500	2.02233400	0.88573200

C	2.35207700	1.44870900	-1.57774200
H	0.34516300	1.04665700	-2.29419400
C	2.51803000	2.13098900	0.74379400
H	0.64512100	2.22483600	1.83369500
C	3.12455500	1.83679700	-0.48125300
H	2.82625100	1.22081300	-2.53250900
H	3.12570500	2.43534600	1.59647300
H	4.20831100	1.91261300	-0.58082200

1 imaginary frequency= -449.1012 cm^{-1}

Zero-point correction= 0.254780 (Hartree/Particle)

Sum of electronic and zero-point Energies= -1755.342607

Sum of electronic and thermal Free Energies= -1755.390600

4a•3H

C	0.91648100	1.36916500	0.89261000
C	1.11418700	2.33847600	-0.10281000
C	2.40077800	2.85256800	-0.35664000
C	3.49541800	2.39989900	0.36767700
C	3.30880700	1.43017400	1.35616200
C	2.03488500	0.93275700	1.61483500
C	-0.50042300	0.85967800	1.16947000
H	2.52391200	3.60775800	-1.13565200
H	4.49078700	2.79872500	0.16080200
H	4.16218600	1.05983800	1.92920200
H	1.90619100	0.17765700	2.39098100
C	-0.62154700	-0.68790300	0.90460400
O	-1.40055300	1.43684300	0.34714100
C	0.06633700	2.87532400	-0.93530300
N	-0.52254100	3.48976400	-1.73595600
C	-0.83947100	1.11721300	2.65981100
H	-1.82829700	0.68727900	2.88170400
H	-0.10787300	0.72081300	3.37838800
H	-0.90309600	2.20891200	2.78425100
H	-1.70325600	-0.89511200	0.88074400
H	-2.75615700	0.79936800	0.58226100
O	-3.60953400	0.28543300	0.83150500
C	-4.30278600	-0.11585300	-0.34390500
C	-4.10007700	-1.62029500	-0.57849400
C	-5.78724900	0.16219700	-0.10392400
C	-3.80849900	0.66357900	-1.56554200
H	-3.04754400	-1.85423000	-0.79883700
H	-4.39284500	-2.18392100	0.32239100

H	-4.70991900	-1.98228700	-1.42267800
H	-5.94869800	1.23864500	0.06627900
H	-6.40622200	-0.15120600	-0.96030800
H	-6.13783200	-0.37866400	0.79028400
H	-4.34042900	0.34233400	-2.47583300
H	-3.98340200	1.74353000	-1.42938100
H	-2.72879300	0.50796600	-1.70011200
S	-0.08565500	-1.30856400	-0.76124400
O	-0.56887500	-2.71668500	-0.82583900
O	-0.52210300	-0.35718200	-1.81199800
C	1.70481500	-1.35248300	-0.79812900
C	2.38572500	-2.40379600	-0.18298400
C	2.36787500	-0.36762100	-1.52648200
C	3.77755100	-2.42948700	-0.25756600
H	1.83463800	-3.18899400	0.33360100
C	3.75849200	-0.41141700	-1.60088200
H	1.79580700	0.41701000	-2.02013400
C	4.46167200	-1.43241500	-0.95850900
H	4.32983800	-3.23893800	0.22487900
H	4.29365700	0.35870200	-2.15997700
H	5.55252800	-1.45933800	-1.01361300
Cl	0.05551600	-1.82899900	2.14540600

0 imaginary frequency

Zero-point correction= 0.392654 (Hartree/Particle)

Sum of electronic and zero-point Energies= -1988.759278

Sum of electronic and thermal Free Energies= -1988.817209

[4a•3H]$^{\neq}$

C	1.02749700	1.36160100	0.83813900
C	1.46225200	2.28174700	-0.13358200
C	2.83462800	2.57028400	-0.28466500
C	3.78246200	1.94308700	0.50987300
C	3.36318600	1.00833600	1.45915000
C	2.00984300	0.73341900	1.61653900
C	-0.43150800	1.02840200	1.09996700
H	3.13919300	3.29150200	-1.04274100
H	4.84038400	2.17166700	0.38131400
H	4.09480400	0.48964100	2.07944700
H	1.70544900	-0.00327000	2.35722400
C	-0.83539600	-0.43819600	0.73131800
O	-1.23932400	1.87064900	0.30994700
C	0.61313800	2.98741100	-1.05239700
N	0.10227400	3.64232400	-1.86784200
C	-0.76123100	1.30771700	2.57787300
H	-1.80891700	1.04545700	2.76782900
H	-0.12703800	0.76245700	3.28314400
H	-0.62857800	2.38374400	2.74317600
H	-2.15476200	-0.27156500	0.59504800
H	-2.15563900	1.46627100	0.33480100
O	-3.28843800	0.31751800	0.65726000
C	-4.22751800	-0.11120700	-0.30864600
C	-4.28812800	-1.64645100	-0.33630600
C	-5.60120600	0.44635400	0.07717000
C	-3.84344800	0.40736600	-1.70295400
H	-3.31141800	-2.06329400	-0.61510100
H	-4.55875200	-2.03110000	0.65762800

H	-5.03706300	-2.00325900	-1.05850000
H	-5.56954000	1.54479500	0.11444200
H	-6.37423700	0.14877900	-0.64709300
H	-5.89824100	0.07781500	1.06939700
H	-4.57321600	0.09032300	-2.46231200
H	-3.80828400	1.50660600	-1.70045700
H	-2.85218400	0.03727200	-1.99109300
S	-0.19976300	-1.09299700	-0.82377000
O	-0.86421100	-2.41686200	-1.03823900
O	-0.39333700	-0.03777500	-1.86175900
C	1.56735400	-1.43034600	-0.76070400
C	2.04015000	-2.51678000	-0.02279900
C	2.42431200	-0.60443200	-1.48349000
C	3.41396900	-2.75379200	0.01341300
H	1.34871500	-3.16997600	0.50454400
C	3.79430900	-0.86084400	-1.45018700
H	2.01764500	0.22527200	-2.05718800
C	4.28949900	-1.92587200	-0.69542400
H	3.80152400	-3.59511700	0.58956100
H	4.47694100	-0.21950400	-2.00848800
H	5.36295300	-2.11827400	-0.66429500
Cl	-0.52776300	-1.66880100	2.01962000

1 imaginary frequency= -1076.3191 cm^{-1}

Zero-point correction= 0.387934 (Hartree/Particle)

Sum of electronic and zero-point Energies= -1988.746672

Sum of electronic and thermal Free Energies= -1988.803966

4b•3H

C	1.54246600	0.47873600	-1.48638400
C	0.77215300	-0.67674300	-1.73508300
C	1.37871300	-1.90137500	-2.07610000
C	2.75667000	-1.99354200	-2.20158400
C	3.53091700	-0.84968200	-1.99896600
C	2.92979100	0.35690800	-1.65197300
C	0.94554900	1.79823000	-0.98104200
H	0.74732500	-2.77369100	-2.24146500
H	3.22081700	-2.94459100	-2.46221400
H	4.61533000	-0.89663900	-2.11002000
H	3.56260900	1.22607400	-1.49554000
C	1.19050900	2.00377800	0.53548700
O	-1.01968900	0.85192300	1.21694100
O	0.76034300	1.23321400	3.03103100
O	-0.45630300	1.82795900	-1.22969500
C	-0.65857600	-0.73180100	-1.67986800
N	-1.79969400	-0.95372500	-1.68557700
C	1.51429300	2.98339900	-1.76070100
H	1.23234500	2.87635500	-2.81628900
H	1.07568800	3.90590700	-1.36269600
H	2.60341800	3.05797400	-1.69601700
S	0.44582900	0.86392400	1.61177300
C	0.94517900	-0.87398200	1.46618900
C	2.30370800	-1.19319000	1.51584100
C	-0.02435500	-1.85693200	1.27624900
C	2.69139000	-2.52349200	1.36396700
H	3.05023800	-0.41388100	1.65779500
C	0.37447400	-3.18703700	1.13723800

H	-1.07331200	-1.57412200	1.21826900
C	1.72947000	-3.52056300	1.17720200
H	3.75064900	-2.78303900	1.38719300
H	-0.37705200	-3.96362400	0.98755000
H	2.03926500	-4.55982400	1.05760600
Cl	2.91249600	2.28563600	0.98744700
H	-0.90164600	1.50388700	-0.42102700
O	-4.63347300	-0.88652300	-1.22383500
C	-4.89500000	-0.01281500	-0.11477300
C	-4.22806500	-0.55870600	1.15029700
C	-4.36797800	1.39172900	-0.42085700
C	-6.40924000	0.00722600	0.04460400
H	-4.56459700	-1.58570800	1.34552800
H	-3.13603200	-0.55282400	1.04383600
H	-4.48189000	0.06169700	2.02101700
H	-3.28072600	1.37133600	-0.56874700
H	-4.83667100	1.78715700	-1.33191600
H	-4.58504100	2.07847700	0.40872900
H	-6.70217000	0.66417000	0.87432500
H	-6.88795300	0.37430700	-0.87351500
H	-6.78765000	-1.00294900	0.25281700
H	-3.67021900	-0.91243100	-1.36515200

0 imaginary frequency

Zero-point correction= 0.393161 (Hartree/Particle)

Sum of electronic and zero-point Energies= -1988.742434

Sum of electronic and thermal Free Energies= -1988.802228

[4b•3H]$^{\neq}$

C	0.85235800	1.24232900	1.11124400
C	0.38424000	2.20074100	0.21469100
C	1.25258000	3.11630100	-0.38672300
C	2.60712000	3.06363900	-0.06767300
C	3.08191800	2.09854200	0.83141100
C	2.21024500	1.18570100	1.42485600
C	-0.24297800	0.34141800	1.65034600
H	0.86535100	3.85589700	-1.08847200
H	3.30176200	3.77072900	-0.52274500
H	4.14665100	2.05875800	1.06616400
H	2.58684100	0.43236300	2.11605100
C	-0.18459600	-1.13690100	1.21723300
O	-0.74194400	-0.57470300	-1.27491400
O	0.02932400	-2.94933300	-0.69060400
O	-1.41931600	0.95144000	1.12450100
C	-1.05204100	2.13620300	-0.03861200
N	-1.96133100	2.57011600	-0.70926600
C	-0.32133400	0.46056600	3.17503900
H	-0.56491600	1.50088800	3.42295400
H	-1.10977600	-0.19647400	3.56373700
H	0.62645100	0.19882700	3.65657400
S	0.15238000	-1.46752100	-0.46490700
C	1.83217000	-1.07696800	-1.01533300
C	2.89579300	-1.89070300	-0.61693300
C	2.04374500	0.05820900	-1.79529900
C	4.19287900	-1.54600600	-0.99876600
H	2.70974600	-2.78652600	-0.02777700
C	3.34295700	0.38865800	-2.17965800

H	1.19557500	0.67297000	-2.08829700
C	4.41807500	-0.40716000	-1.77824900
H	5.03125600	-2.17457900	-0.69362300
H	3.51609200	1.27701600	-2.78866400
H	5.43425500	-0.14382500	-2.07631200
Cl	0.97764500	-2.11225200	2.22327400
H	-2.49082900	0.52500800	0.67617300
O	-3.51656400	0.76975000	0.15889900
C	-4.22103000	-0.21352200	-0.66484300
C	-3.82752000	-0.02821700	-2.12590000
C	-3.82837600	-1.59446100	-0.15740400
C	-5.70830200	0.04841600	-0.46153100
H	-4.10190900	0.97470200	-2.47995200
H	-2.75009800	-0.17461000	-2.25124200
H	-4.36194300	-0.76212000	-2.74288800
H	-2.75190900	-1.75553200	-0.28171400
H	-4.08402900	-1.70240800	0.90524800
H	-4.37211100	-2.36341900	-0.72066900
H	-6.30192700	-0.66256800	-1.05137800
H	-5.98035400	-0.06489400	0.59593400
H	-5.96994100	1.06553300	-0.78278300
H	-3.08238900	1.59413100	-0.36903700

1 imaginary frequency= -1053.4032 cm^{-1}

Zero-point correction= 0.386826 (Hartree/Particle)

Sum of electronic and zero-point Energies= -1988.708035

Sum of electronic and thermal Free Energies= -1988.763520

6b

C	-0.95187800	0.76432200	0.86800200
C	-1.34608400	1.72527000	-0.05464500
C	-0.77740800	2.99769000	-0.08222600
C	0.21471800	3.28454300	0.85309000
C	0.61866900	2.31465700	1.78492800
C	0.03733500	1.04623200	1.80799300
C	-1.74495200	-0.49870900	0.64767900
H	-1.10391700	3.73783600	-0.81300300
H	0.68735400	4.26744800	0.86117200
H	1.40336800	2.55837200	2.50260900
H	0.35683300	0.29862400	2.53257100
C	-0.97416400	-1.74874100	0.19514700
O	-0.52953400	-0.86898800	-2.24430000
O	0.79655000	-2.89026100	-1.39359800
O	-2.63683200	-0.12217000	-0.45866400
C	-2.40058200	1.14194200	-0.88732900
N	-3.02301700	1.71478300	-1.84488100
C	-2.63947000	-0.80244300	1.84961100
H	-3.21871800	-1.71654300	1.67117600
H	-2.04130900	-0.92990500	2.75746600
H	-3.32495100	0.04098000	1.99885600
S	0.18251400	-1.54774900	-1.10982500
C	1.59031600	-0.47930800	-0.72276100
C	2.58676900	-0.94769500	0.13684500
C	1.63227100	0.81262900	-1.24397500
C	3.63550000	-0.09626900	0.48585100
H	2.54697500	-1.96625300	0.51767500
C	2.68922800	1.65261300	-0.89474800

H	0.84074000	1.14984700	-1.90980400
C	3.68692600	1.20313100	-0.02709900
H	4.42032500	-0.45168300	1.15566600
H	2.72824500	2.66610100	-1.29619900
H	4.50985800	1.86469400	0.24809900
Cl	-0.12013200	-2.52932100	1.59817900
H	-3.70629900	1.07160200	-2.24962800

0 imaginary frequency

Zero-point correction= 0.257781 (Hartree/Particle)

Sum of electronic and zero-point Energies= -1755.365932

Sum of electronic and thermal Free Energies= -1755.412829

6c

C	-0.10560700	1.08543500	0.92147800
C	-0.04206000	2.10927800	-0.03852900
C	1.16730100	2.80360400	-0.18009000
C	2.26712300	2.53405300	0.63008800
C	2.18099100	1.53985000	1.60769600
C	0.99793600	0.82155900	1.74606600
C	-1.32562700	0.26724800	1.16566000
H	1.21762900	3.57521000	-0.94848100
H	3.19302300	3.09711800	0.50012500
H	3.03577500	1.31151000	2.24535000
H	0.93195100	0.01907200	2.48182700
C	-1.59475400	-0.83449800	0.44429100
O	-0.84964500	-0.40901900	-2.12813700
O	-1.01901900	-2.79954400	-1.25095300
O	-0.93419100	3.06217500	-2.02460000
C	-1.21448100	2.53313100	-0.90152500
N	-2.39718300	2.34559600	-0.34926400
C	-2.11483900	0.67740200	2.37009200
H	-3.07703800	0.16444000	2.44992200
H	-1.52102800	0.46104900	3.27170000
H	-2.27777800	1.75940600	2.32229500
S	-0.64933300	-1.37435800	-1.01434800
C	1.07455400	-1.35018400	-0.54306100
C	1.50395000	-2.24183100	0.43846500
C	1.93836400	-0.48604900	-1.20581000
C	2.85643100	-2.25527000	0.77138100
H	0.79743300	-2.90741600	0.93382900

C	3.29127100	-0.51878700	-0.86747000
H	1.55185700	0.20546400	-1.95170500
C	3.74677200	-1.39644000	0.11797100
H	3.21816500	-2.93905300	1.54002800
H	3.98806200	0.15483500	-1.36658200
H	4.80446800	-1.41079300	0.38424100
Cl	-2.99639600	-1.83329900	0.71757300
H	-3.09527200	2.68588700	-1.01757800

0 imaginary frequency

Zero-point correction= 0.256280 (Hartree/Particle)

Sum of electronic and zero-point Energies= -1755.367605

Sum of electronic and thermal Free Energies= -1755.416961

7b

C	-0.94442400	0.83168000	0.86498300
C	-1.24264700	1.80318700	-0.08859500
C	-0.62397600	3.04980700	-0.09488300
C	0.32952100	3.30706700	0.88984800
C	0.64046900	2.33055700	1.84808100
C	0.00039000	1.08840300	1.85516400
C	-1.78072600	-0.40871000	0.61837500
H	-0.87686200	3.79304800	-0.85197600
H	0.84324900	4.26893800	0.91494100
H	1.39787300	2.54656000	2.60333800
H	0.24721100	0.34132300	2.60790800
C	-1.04645400	-1.71374000	0.24992900
O	-0.55568000	-1.06043600	-2.25972900
O	0.71007700	-3.02636000	-1.22251300
O	-2.71088500	1.77762700	-2.04191000
C	-2.24755700	1.24979100	-1.02740900
N	-2.56899100	0.02484900	-0.53672300
C	-2.72570100	-0.65324800	1.80498700
H	-3.35846100	-1.52804400	1.60698700
H	-2.16681300	-0.82549800	2.73050900
H	-3.36242200	0.23034000	1.93772500
S	0.12636900	-1.65133900	-1.05841500
C	1.54424700	-0.58166700	-0.72569600
C	2.54118000	-1.02378300	0.14691800
C	1.58465700	0.69513400	-1.28414300
C	3.58983800	-0.16174600	0.46997700
H	2.50174500	-2.03093300	0.55701700
C	2.63927000	1.54679100	-0.95760300

H	0.79621900	1.01097000	-1.96403500
C	3.63817900	1.12299600	-0.07812000
H	4.37581900	-0.49722900	1.14866700
H	2.67708000	2.54839800	-1.38767600
H	4.45964700	1.79377300	0.17854900
Cl	-0.20315700	-2.42163600	1.70360100
H	-3.04526400	-0.65812700	-1.10912500

0 imaginary frequency

Zero-point correction= 0.258621 (Hartree/Particle)

Sum of electronic and zero-point Energies= -1755.395741

Sum of electronic and thermal Free Energies= -1755.442704

(R,R)-7+3^{-}

C	-1.31992000	0.60833400	0.45010900
C	-2.65164000	0.75577000	0.06441700
C	-3.17633900	1.97801100	-0.33908400
C	-2.32304700	3.08286000	-0.34126700
C	-0.99311700	2.94907400	0.07771100
C	-0.47943100	1.71383100	0.48539400
C	-1.05908200	-0.83746900	0.83407600
H	-4.22280100	2.06297400	-0.63292600
H	-2.69569100	4.05936200	-0.65312300
H	-0.34387300	3.82555900	0.09285200
H	0.54844600	1.64550800	0.82965800
C	-0.19552400	-1.63547000	-0.19629800
O	2.14819300	-2.34599900	-1.29204700
O	1.95855200	-2.29969100	1.26090900
O	-4.52180400	-0.81627500	-0.00459200
C	-3.34639100	-0.54384100	0.22032700
N	-2.40051500	-1.39782500	0.70973100
C	-0.53983900	-0.97077100	2.26425900
H	-1.28021700	-0.52244300	2.93587500
H	-0.39077700	-2.02030100	2.53926100
H	0.40990600	-0.44204000	2.38917200
Cl	-0.65090700	-1.26051900	-1.87971900
S	1.67113700	-1.67202400	-0.05650300
C	2.27337700	0.00067000	-0.04848300
C	2.76195200	0.52599300	1.14706000
C	2.22736000	0.73598300	-1.23354200
C	3.20182500	1.84985300	1.15554500
H	2.79686900	-0.09042700	2.04380700

C	2.65980600	2.06051400	-1.20286700
H	1.86140400	0.28624500	-2.15432600
C	3.13893000	2.61525600	-0.01234000
H	3.58963200	2.28458800	2.07650100
H	2.62304200	2.66128200	-2.11152400
H	3.47259300	3.65327100	0.00387000
H	-2.60923600	-2.36511600	0.91788700
H	-0.38151200	-2.70833600	-0.05009600

0 imaginary frequency

Zero-point correction= 0.272950 (Hartree/Particle)

Sum of electronic and zero-point Energies= -1755.876134

Sum of electronic and thermal Free Energies= -1755.923012

(R,S)-7+3^{-}

C	-0.42343600	1.20827800	0.71631000
C	-0.56796900	2.34107800	-0.08115200
C	0.43075100	3.30454600	-0.17502500
C	1.59035900	3.11023700	0.57707500
C	1.71531100	1.99838900	1.42159100
C	0.70340000	1.03887300	1.51021800
C	-1.65357000	0.33345900	0.58780300
H	0.29853300	4.18496400	-0.80413000
H	2.39949700	3.83970500	0.52667400
H	2.61898600	1.87885900	2.01982900
H	0.80614900	0.18128800	2.17289100
C	-1.36405800	-0.86830800	-0.36206300
O	-0.12688900	-3.20332300	-0.87297100
O	-0.01470000	-2.33762200	1.53284000
O	-2.43760000	3.15930700	-1.42687400
C	-1.92687000	2.33525600	-0.67509500
N	-2.53315800	1.21490800	-0.17358100
C	-2.24691200	-0.05885400	1.93386900
H	-2.40850500	0.84953700	2.52422800
H	-3.20615900	-0.57240200	1.80817500
H	-1.56204800	-0.72297800	2.47064500
Cl	-2.82303900	-1.87278900	-0.61343700
S	0.01568700	-2.06289200	0.07096100
C	1.53811800	-1.20741300	-0.32279700
C	2.58624700	-1.34229700	0.58480000
C	1.66395600	-0.48684700	-1.50932800
C	3.79721500	-0.71477700	0.29879900
H	2.44590500	-1.90716300	1.50516900

C	2.87326400	0.15626300	-1.76701300
H	0.84815000	-0.41509500	-2.22689700
C	3.93519300	0.04362000	-0.86563600
H	4.62974100	-0.80664900	0.99653400
H	2.98632900	0.74333900	-2.67842900
H	4.87785800	0.55053100	-1.07467600
H	-3.45070900	0.92230900	-0.48249800
H	-1.08343900	-0.48281800	-1.34750000

0 imaginary frequency

Zero-point correction= 0.272242 (Hartree/Particle)

Sum of electronic and zero-point Energies= -1755.876070

Sum of electronic and thermal Free Energies= -1755.923691

4. References

(1) Austin, A.; Petersson, G. A.; Frisch, M. J.; Dobek, F. J.; Scalmani, G.; Throssell, K. A Density Functional with Spherical Atom Dispersion Terms. *J. Chem. Theory Comput.* **2012**, *8* (12), 4989–5007. https://doi.org/10.1021/ct300778e.

(2) Bruker. APEX3, SAINT and SADABS. Bruker AXS Inc, Madison, Wisconsin, USA, **2015**.

(3) Sheldrick, G. M. *Acta Cryst.* **2008***, A64*, 112.

(4) Sheldrick, G. M. *Acta Cryst.* **2015***, C71*, 3.

(5) Dolomanov O. V., Bourhis L. J., Gildea R. J., Howard J. A. K, Puschmann H. *J. Appl. Cryst.*, **2009**, 339.
